# A comprehensive and quantitative review of dark fermentative biohydrogen production

**DOI:** 10.1186/1475-2859-11-115

**Published:** 2012-08-27

**Authors:** Simon Rittmann, Christoph Herwig

**Affiliations:** 1 Institute of Chemical Engineering, Research Area Biochemical Engineering, Gumpendorferstraße 1a, Vienna University of Technology, Vienna, 1060, Austria

**Keywords:** Hydrogen yield, Volumetric hydrogen productivity, Specific hydrogen productivity, Closed batch, Batch, Chemostat culture, Fed-batch, Bioprocess, Result normalization, Scalable physiological parameters

## Abstract

Biohydrogen production (BHP) can be achieved by direct or indirect biophotolysis, photo-fermentation and dark fermentation, whereof only the latter does not require the input of light energy. Our motivation to compile this review was to quantify and comprehensively report strains and process performance of dark fermentative BHP. This review summarizes the work done on pure and defined co-culture dark fermentative BHP since the year 1901. Qualitative growth characteristics and quantitative normalized results of H_2_ production for more than 2000 conditions are presented in a normalized and therefore comparable format to the scientific community.

Statistically based evidence shows that thermophilic strains comprise high substrate conversion efficiency, but mesophilic strains achieve high volumetric productivity. Moreover, microbes of *Thermoanaerobacterales* (Family III) have to be preferred when aiming to achieve high substrate conversion efficiency in comparison to the families *Clostridiaceae* and *Enterobacteriaceae*.

The limited number of results available on dark fermentative BHP from fed-batch cultivations indicates the yet underestimated potential of this bioprocessing application. A Design of Experiments strategy should be preferred for efficient bioprocess development and optimization of BHP aiming at improving medium, cultivation conditions and revealing inhibitory effects. This will enable comparing and optimizing strains and processes independent of initial conditions and scale.

## Introduction

A possibility to circumvent the production of non-carbon neutral greenhouse gasses, such as carbon dioxide (CO_2_), is the development and continuous investigation of alternative biofuels. One promising alternative fuel is biologically generated hydrogen (H_2_), which is referred to as biohydrogen. Biohydrogen production (BHP) has initially been described in the 19^th^ century [1,2], but elucidation of dark fermentative biohydrogen production was only done since 1901 [3,4]. Dark fermentative BHP is a carbon neutral process for production of H_2_ and CO_2_ from biomass by facultative and obligate anaerobic microorganisms.

H_2_ offers many beneficial features, such as being harmless to mammals and the environment [5-7]. Moreover, H_2_ is regarded as a non-polluting fuel, because its combustion product is water (H_2_O). When comparing volumetric energy densities at normal conditions for H_2_ and methane (CH_4_) a drawback of H_2_ is emerging, since H_2_ comprises 3.00 kWh m^-3^ in comparison to 9.97 kWh m^-3^ for CH_4_[8]. Moreover, H_2_ storage and transmission problems have been addressed [6,9-12], but research for optimization is ongoing [13].

H_2_ may be produced by a number of different processes including electrolysis of H_2_O, thermocatalytic reformation of hydrogen rich substrates and various biological processes [14-18]. To date, H_2_ is mainly produced by electrolysis of H_2_O and steam reformation of CH_4_. These processes are very energy intensive, but can be simply performed. Biological processes can be carried out at ambient temperatures, but they are more sophisticated in design and performance [18,19]. BHP can be achieved by direct or indirect biophotolysis, photo-fermentation and dark fermentation [15,16,18,20-25].

The main advantage of dark fermentative BHP is that the hydrogen evolution rate (HER) [mmol L^-1^ h^-1^ is higher in contrast to other BHP processes [11,12,17,26]. Major known drawbacks of dark fermentative BHP are the low yield of H_2_ per substrate consumed (Y_(H2/S)_) [mol mol^-1^, which is due to metabolic fundamentals [27]. Moreover, concomitant production of carbon rich metabolites (*i.e.* organic acids, alcohols) and CO_2_ is shown [28] and must be individually evaluated for each strain. CO_2_ can be removed or separated from H_2_, sequentially stored in biomass [29], or converted to other substances, such as CH_4_[30,31]. Basic microbiological investigations and bioprocess engineering research was performed to increase the overall strain performance of BHP during fermentation of pure microorganisms [32-34].

For a structured approach, we classified the vast amount of information available in literature in respect to the process modes, such as closed batch, batch, chemostat culture and fed-batch conditions and quantified the main performance attributes. We reviewed the exciting work published on microbial strains capable of dark fermentative BHP with the aim to demonstrate the versatile portfolio of H_2_ producing genera and the wide range of possible substrates for this purpose. In the present contribution the Y_(H2/S),_ HER and specific hydrogen productivity (qH_2_) [mmol g^-1^ h^-1^] of the families *Clostridiaceae*, *Enterobacteriaceae* and *Thermoanaerobacterales* (Family III), as well as mesophilic and thermophilic cultivation conditions have been statistically compared. We analyse the microbiological and biochemical engineering approaches for optimization of H_2_ production and provide a comprehensive summary of the current status of dark fermentative BHP from more than 2000 different conditions. Herewith we want to stress that more quantitative work is urgently needed to turn this natural capacity into economic processes, based on physiological scalable parameters, which allow comparison and targeted optimization. Therefore, as a significant contribution to future work we propose a set of physiological scalable parameters for normalized results.

### Classification and quantification tasks

In order to show a complete picture of each strain’s H_2_ production potential the results of dark fermentative BHP are presented in Additional files 1, 2, 3, 4, 5. Qualitative and quantitative characteristics are summarized as follows: taxonomic classification (genus, species, strain), quantitative performance attributes of BHP (Y_(H2/S)_ [mol mol^-1^] (substrate conversion efficiency), the HER [mmol L^-1^ h^-1^] (volumetric productivity) and the qH_2_ [mmol g^-1^ h^-1^] (biological production capacity)), and qualitative attributes (*i.e.* pH, temperature and substrate). Moreover, we introduce a new categorization system in order to subclassify results according to the experimental set-up. This was required since many experiments have been conducted in sealed vials. We denote this cultivation technique as “closed batch”. This is a very prominent microbiological cultivation technique, which has to be distinguished from batch cultivation in open systems ( *i.e.* bioreactors). Thereafter, the following categorization was used: batch, chemostat culture and fed-batch.

Many authors stress for the importance to uniformly present yield and rates of BHP [11,12,35]. Result comparison is most suitably to be achieved by using culture dependent Y_(H2/S)_ and qH_2_, as well as the non-culture dependent HER, because these units completely describe the strains H_2_ production characteristics. Moreover, the yield and rates are independent of scale and initial process conditions. We are certainly aware of the fact that presentation of results is even more advantageous based on a C-molar basis of the substrate [36]. Therefore, experiments have to be performed on defined media rather than on complex media, because the calculation of C-molar yield and productivities is not possible when analysing the performance in complex media. Thus, sophisticated analysis methods need to be considered for the evaluation of Y_(H2/S)_, HER and qH_2_ based on C-molar mass balance. In this respect, we want to generally stress the importance of result presentation using mass balances, which is very important for quality assurance and must not be omitted [37]. Complete quantitative comparison of dark fermentative BHP would become possible if a C-molar basis of result presentation is used throughout the scientific community.

## Review

### Pure and defined co-culture experiments

A summarization the distribution of quantitative results obtained from different experimental set-ups of dark fermentative BHP is shown in Table 1. It becomes obvious that Y_(H2/S)_ is most often presented, which is followed by the HER, whereas qH_2_ is described in less extent. Most studies on Y_(H2/S)_ or HER were performed by either closed batch or batch fermentation (Additional files 1,2). A special case represents the fed-batch fermentation, whereof only five results for Y_(H2/S)_ can be found in literature [38]. Results of BHP from defined co-culture examinations are also presented within the Additional files 1,2,3,4.

**Table 1:** Overview of dark fermentative BHP in respect to the cultivation technique

**Culture parameter**	**Closed batch**	**Batch**	**Chemostat culture**	**Fed-batch**
Y_(H2/S)_	441	425	253	5
HER	329	333	171	0
qH_2_	68	78	99	0

### Non-quantitative H_2_ production

Many dark fermentative BHP strains were isolated and characterized, but quantitative information on H_2_ production is missing. These strains and their corresponding growth requirements can serve as a pool to extend microbiological and bioprocess engineering examinations to new taxa. Furthermore, we are often confronted with the fact that quantitative results are assessed, but cannot be normalized by using the units Y_(H2/S)_, HER or qH_2_, because of missing or undefined entities for recalculation of presented results. Consequently, these results and corresponding conditions are assigned to Additional file 1, because we have not been able to normalize these results for comparison purposes.

### Closed batch H_2_ production

Dark fermentative BHP is found to be most often performed by strain cultivation in closed vessels (Additional file 2). Closed batch technique offers the main advantage that highly sophisticated bioprocess cultivation set-up for research can be omitted. Moreover, simple incubation conditions may be easily accomplished, because only incubation in H_2_O or air bath is necessary. In our opinion a closed batch investigation is highly advantageous in order to examine physical factors affecting BHP. For instance the elucidation of optimum temperature values, the effect of gas pressure, the influence of illumination or the investigation of agitation can be investigated. In this respect the inhibition of CO_2_ and H_2_ was described [39,40]. Additionally, by using closed batch technique, the substrate utilization spectrum can be investigated. This mode has the advantage to screen fast, determine optimal physical parameters, and describe their relationship to the physiological performance. Hence, the application closed batch is indeed of great value. The elucidation of chemical factors on BHP, such as the pH value seems to be rather difficult, because balanced growth at a certain pH value by means of base addition cannot be simply achieved. The investigation of the initial substrate concentration and medium amendments in order to optimize medium composition has been conducted, and the results led to an optimized medium composition [41,42].

A disadvantage of the closed batch technique is the discontinuous monitoring of culture parameters and the occurrence of unstable culture conditions due to sample removal and/or inhibition of BHP by build up of liquid and gaseous metabolic end products, because these excreted cellular end products cannot be continuously removed from the closed culture vessel. Manipulation to the culture vessel or to the culture itself requires at least the disruption of one physical factor. This unavoidably results in non-continuous cultivation conditions, making the utilization of closed batch technique rather unattractive, if sampling occurs more than once, because the culture response to changes in environmental conditions occurs rapidly [43]. Considering advantages and disadvantages of closed batch investigation the most urgent question to be addressed is: how quantitative is closed batch? Although, balanced growth may not be achieved, H_2_ production and growth kinetics were successfully investigated using closed batch technique [41,42]. End product inhibition occurring during closed batch investigation resulting from the production of solvents, organic acids, alcohols, CO_2_ or H_2_ partial pressure build-up certainly influences the results [39,40]. Hence, closed batch systems can be used for fast screening, but open cultivation systems need to be used for subsequent examination of the physiological potential of the strain and for quantitative bioprocess development.

### Batch, chemostat culture and fed-batch H_2_ production

We provide an overview of dark fermentative BHP in bioreactors and similar set-ups, such as modified Erlenmeyer-flasks and refer to these examination techniques as open systems, because gas sparging, offgas composition determination, pH titration and medium supplementation can be performed. By using a highly controlled and automated set-up it is possible to quantitatively describe the strains inherent H_2_ production capacity and growth kinetics. Usually, this is performed by using fully automated and controlled bioreactor set-ups [34,44]. We compare the strains based on their BHP potential on glucose, and do not distinguish between growth on complex or defined medium. Furthermore, the pH value and temperature was not taken into account for comparison purposes.

#### Batch H_2_ production

Many quantitative investigations related to biohydrogen production were conducted by using batch type fermentations. Hereof, the genera *BacillusCaldicellulosiruptorClostridiumEnterobacter* and *Escherichia* were most widely studied (Additional file 3). Based on Y_(H2/S)_ we identified *Caldicellulosiruptor owensensis* DSM 13100 [44] and *Enterobacter cloacae* DM 11 [33] showing highest Y_(H2/S)_ of 4.0 and 3.9 mol mol^-1^, respectively. The highest HER of 32 mmol L^-1^ h^-1^ is shown for *Enterobacter cloacae* II BT-08 [32], which is followed by *Clostridium* sp. strain no. 2 showing a HER of 27 mmol L^-1^ h^-1^[45]. When analysing results for the highest qH_2_ we reveal that *Caldicellulosiruptor saccharolyticus* DSM 8903 produces 23 mmol g^-1^ h^-1^[28].

#### Chemostat culture H_2_ production

Dark fermentative BHP has often been investigated in chemostat culture. The results are summarized in Additional file 4. Thereof *Caldicellulosiruptor saccharolyticus* DSM 8903 is identified to comprise the highest Y_(H2/S)_ of 4.0 mol mol^-1^[46]. Highest HER of 77 mmol L^-1^ h^-1^ is reported for *Enterobacter cloacae* II BT-08 [47]. The highest qH_2_ of 35 mmol g^-1^ h^-1^ is identified for *Caldicellulosiruptor kristjanssonii* DSM 12137 [34].

#### Fed-batch H_2_ production

The literature survey of dark fermentative BHP revealed only five conditions which have been operated in fed-batch mode (Additional file 5). The quantitative presentation of these results is restricted to the Y_(H2/S)_. The maximum Y_(H2/S)_ is identified for a recombinant strain of ATCC 25755 comprising 2.15 mol mol^-1^[38]. The limited number of results available for dark fermentative BHP from fed-batch fermentation is due to the fact that usually the application of this technique leads to massive accumulation of organic acids and other reduced end products (*i.e.* alcohols) in the culture broth, strongly inhibiting the growth and H_2_ production kinetics. Consequently, fed-batch investigation can be applied only by using broth exchange or cell separation systems [38]. Based on such experimental set-ups high cell densities and feed flow rates above the maximum specific growth rate can be reached [36]. The potential of fed-batch cultivation for H_2_ production is yet underestimated in terms of quantity and quality (Additional file 5). Thus, the quantitative potential of fed-batch cultivation has to be exploited in more detail for dark fermentative BHP by using biochemical engineering principles.

## Discussion

### Comparison of H_2_ production performance of strains related to *Clostridiaceae*, *Enterobacteriaceae* and *Thermoanaerobacterales* (Family III)

**Figure 1:**
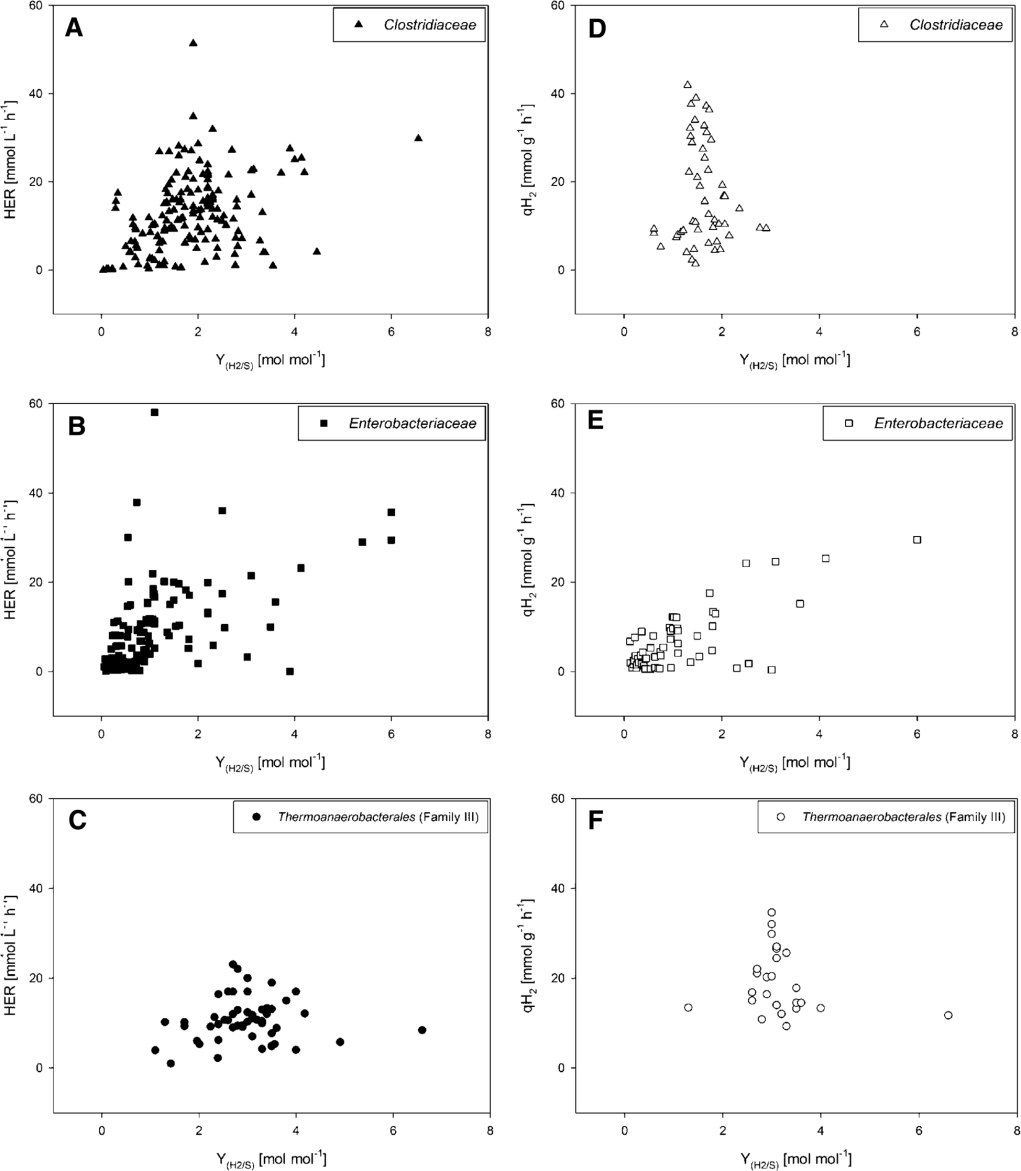
**A graphical overview is shown irrespective of the utilization of the carbon substrate, medium composition and cultivation conditions of the HER of*Clostridiaceae*(A),*Enterobacteriaceae*(B) and*Thermoanaerobacterales*(Family III) (C) plotted against the Y**_**(H2/S)**_**. The qH**_**2**_**of*Clostridiaceae*(D),*Enterobacteriaceae*(E) and*Thermoanaerobacterales*(Family III) (F) is shown in relation to the Y**_**(H2/S)**_**.** It is indicated that *Clostridiaceae* and *Enterobacteriaceae* perform better than *Thermoanaerobacterales* (Family III) in respect to the volumetric productivity. The qH_2_ for *Clostridiaceae* and *Thermoanaerobacterales* (Family III) is shown to be higher than for *Enterobacteriaceae*. Regarding the substrate conversion efficiency (Y_(H2/S)_) the following ranking is indicated: *Thermoanaerobacterales* (Family III) >  *Clostridiaceae* >  *Enterobacteriaceae.*

**Figure 2:**
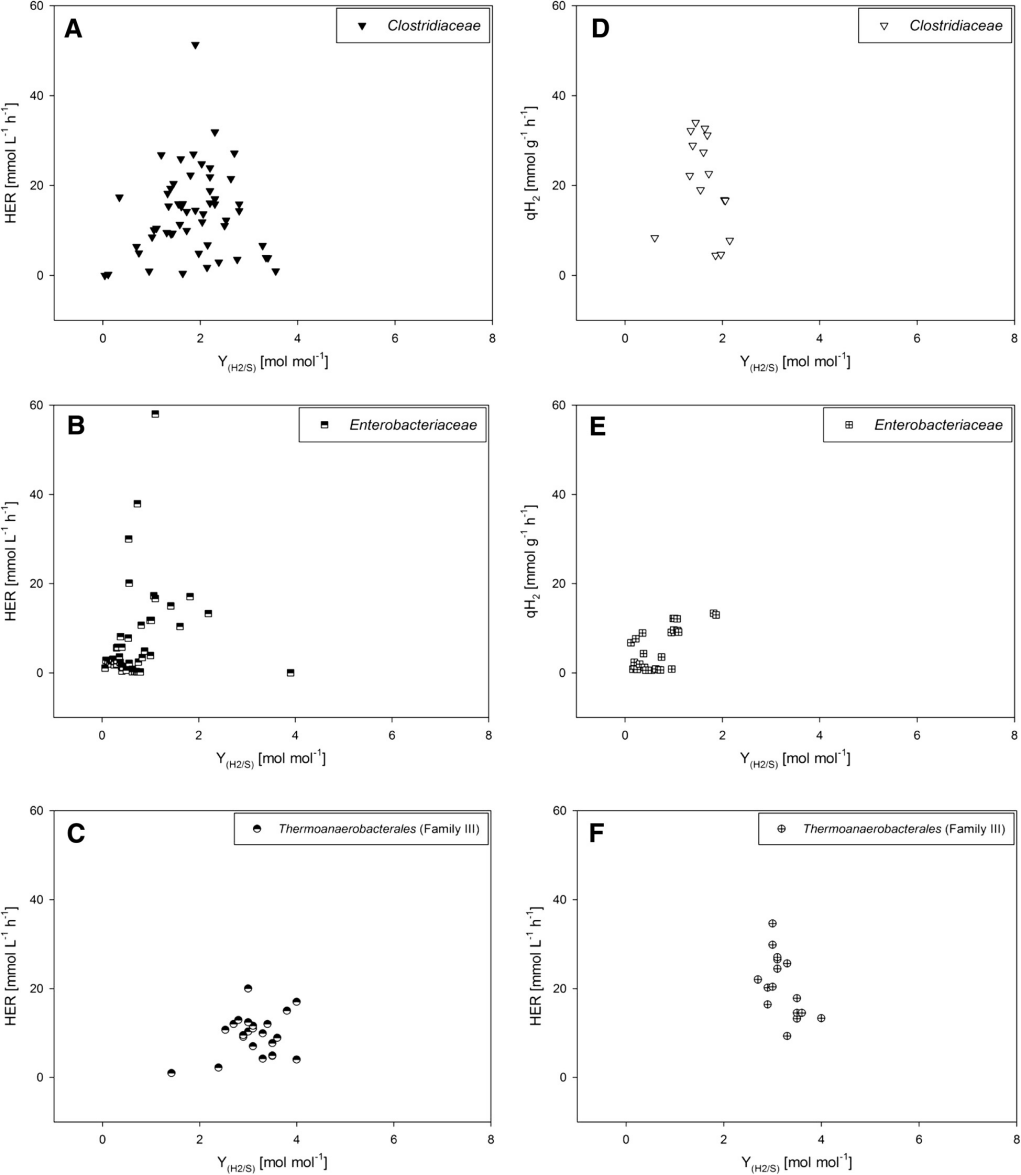
**A graphical overview of the utilization of glucose is presented. HER of*Clostridiaceae*(A),*Enterobacteriaceae*(B) and*Thermoanaerobacterales*(Family III) (C) is shown in relation to the Y**_**(H2/S)**_**. The qH**_**2**_**of*Clostridiaceae*(D),*Enterobacteriaceae*(E) and*Thermoanaerobacterales*(Family III) (F) in relation to the Y**_**(H2/S)**_**is also presented.** These results are depicted irrespective of the medium composition, cultivation conditions or genetic modification. These graphs offer an indication that *Clostridiaceae* and *Enterobacteriaceae* show a higher volumetric productivity than *Thermoanaerobacterales* (Family III). In respect to the qH_2_*Clostridiaceae* and *Thermoanaerobacterales* (Family III) show higher productivity than *Enterobacteriaceae*. Based on the Y_(H2/S)_ following ranking is presented: *Thermoanaerobacterales* (Family III) >  *Clostridiaceae* >  *Enterobacteriaceae.*

As summarized in Additional files 2,3,4,5 quantitative examination of dark fermentative BHP is largely performed on strains phylogenetically related to either the family *Clostridiaceae* or *Enterobacteriaceae*, but also strains belonging to the family *Thermoanaerobacterales* (Family III) receive increasing scientific attention, because they comprise certain beneficial metabolic features [46,48-51]. According to Table 2 less results for qH_2_ than for HER compared to the Y_(H2/S)_ are described. This discrepancy in the number of results available in literature is interesting, because during research the determination of biomass concentration and H_2_ offgas content could be easily performed. We analysed *ClostridiaceaeEnterobacteriaceae* and *Thermoanaerobacterales* (Family III) in order to elucidate differences of the performance of these families concerning HER and qH_2_ in respect to Y_(H2/S)_. The basis for comparison is either any carbon substrate (Figure 1) or glucose (Figure 2), but irrespectively of growth conditions and metabolic modifications.

**Table 2:** **Results of the statistical analysis for*Clostridiaceae*,*Enterobacteriaceae*and*Thermoanaerobacterales*(Family III)**

**Y**_**(H2/S)**_	***Clostridiaceae*** (n = 464)	***Enterobacteriaceae*** (n = 295)	***Thermoanaerobacterales*** (Family III) (n = 73)
[mol mol^-1^]			
Median	1.785	0.82	2.9
Mean	1.87 ± 1.10^*^	1.15 ± 1.34^*^	2.92 ± 1.18^*^
**HER**	***Clostridiaceae*** (n = 317)	***Enterobacteriaceae*** (n = 318)	***Thermoanaerobacterales*** (Family III) (n = 38)
[mmol L^-1^ h^-1^]			
Median	8.67	4.915	9.6
Mean	9.75 ± 8.41	11.37 ± 17.71	8.98 ± 3.40
**qH**_**2**_	***Clostridiaceae*** (n = 70)	***Enterobacteriaceae*** (n = 102)	***Thermoanaerobacterales*** (Family III) (n = 20)
[mmol g^-1^ h^-1^]			
Median	10.05	3.75	16.615
Mean	14.49 ± 11.24	12.90 ± 26.02	18.61 ± 7.14

These three culture parameters are plotted against each other on any carbon substrate (Figures 1A-1F). Based on the HER *Clostridiaceae* and *Enterobacteriaceae* comprise highest volumetric productivity, whereas in respect to the specific H_2_ productivity *Clostridiaceae* and *Thermoanaerobacterales* (Family III) are indicated to perform better. Nonetheless, a clear trend towards better substrate conversion efficiency is revealed: *Thermoanaerobacterales* (Family III) >  *Clostridiaceae* >  *Enterobacteriaceae*. Secondly, the HER, qH_2_ and Y_(H2/S)_ are graphically analysed for the growth of the three families on glucose, but independent of the utilization of complex or defined medium, cultivation conditions or metabolic modifications (Figures 2A-2F). In principle analogous trends for the HER, qH_2_ and Y_(H2/S)_ of the three families can be shown for the growth on glucose compared to the growth on any carbon substrate.

Above paragraph described the interrelations between the physiological parameters of *ClostridiaceaeEnterobacteriaceae* and *Thermoanaerobacterales* (Family III). Subsequently, we want to statistically evaluate their dark fermentative BHP potential. Firstly, a comparison has been done based on the median in order to identify a superior performance of one of the three families regarding the Y_(H2/S)_, HER and qH_2_[52]. Secondly, the mean between these families has been individually analysed by using the Welch-test, or where applicable by using the Student’s t-test, at a level of significance of p = 0.01 [52]. Normalized results available in the Additional files 2,3,4,5 have been used for comparison purposes. The results are summarized in Table 2.

By comparing the median in respect to the Y_(H2/S)_, HER and qH_2_ we can show that strains perform as follows: *Thermoanaerobacterales* (Family III) >  *Clostridiaceae > Enterobacteriaceae*. Statistical analysis of the mean only allows a most significant statement for the Y_(H2/S)_, but not for the HER and qH_2_. Herewith we present evidence that strains of *Thermoanaerobacterales* (Family III) perform most significantly better in respect to the Y_(H2/S)_ of *Clostridiaceae* and *Enterobacteriaceae*. Also strains from the family *Clostridiaceae* are found to comprise a most significantly higher Y_(H2/S)_ than strains of the family *Enterobacteriaceae*. Based on our statistical investigation we reveal that strains of the family *Thermoanaerobacterales* (Family III) have to be clearly preferred when aiming to achieve a high Y_(H2/S)_ in comparison to the families *Clostridiaceae* and *Enterobacteriaceae*.

### Comparison of mesophilic and thermophilic H_2_ production

In order to compare the dark fermentative BHP performance of mesophilic (20-44 °C) and thermophilic (45-80 °C) strains we used the results shown in Additional files 2, 3, 4, 5. As mentioned above statistical analysis has been carried out to evaluate the difference in terms of Y_(H2/S)_, qH_2_ and HER. The results are summarized in Table 3. We can clearly show that thermophilic strains are superior to mesophilic strains in respect to the Y_(H2/S)_. This result is also supported in the values for the median, which also shows the higher Y_(H2/S)_ of thermophilic strains. Herewith most significant evidence is presented to favour mesophilic over thermophilic strains in respect to the HER, which is also reflected in the value determined for the median. Unfortunately our statistical analysis of qH_2_ cannot present a beneficial result of one or the other group, but nevertheless the mean and the median show a trend to favour thermophilic over mesophilic strains. Thus, we are able to present statistical evidence demonstrating to use thermophilic strains when aiming on a high Y_(H2/S)_, but to use mesophilic strains to achieve a high HER.

**Table 3:** Statistical analysis of mesophilic and thermophilc dark fermentative BHP

**Y**_**(H2/S)**_	**Mesophilic**	**Thermophilic**
[mol mol^-1^]	(n = 695)	(n = 244)
Median	1.22	2.30
Mean	1.46 ± 1.18^*^	2.20 ± 1.42^*^
**HER**	**Mesophilic**	**Thermophilic**
[mmol L^-1^ h^-1^]	(n = 587)	(n = 128)
Median	6.29	3.89
Mean	9.92 ± 13.27^*^	5.62 ± 5.45^*^
**qH**_**2**_	**Mesophilic**	**Thermophilic**
[mmol g^-1^ h^-1^]	(n = 147)	(n = 50)
Median	7.65	9.70
Mean	11.53 ± 16.00	12.51 ± 10.79

### Microbiological potential for enhancing H_2_ production

#### Microbial potential of H_2_ production by strain isolation

The initial microbiological investigation of strains for biological H_2_ production offers the opportunity to characterize the microbes in full detail, for instance in respect to pH, temperature and substrate utilization spectrum. Moreover, phylogenetical information will eventually be retrieved during strain characterization. We suggest using the information on strains and conditions available in Additional files 1,2,3,4,5 to extend studies of dark fermentative BHP to broader substrate diversity. These strains offer promising experimental endeavours to microbiologists for physiological studies, because many of these strains are yet not characterized in detail [53-56]. Many wild-type strains were found to comprise a high Y_(H2/S)_ and high qH_2_[44,51,57]. Moreover, basic research efforts need to be increased to isolate novel dark fermentative BHP strains from the environment, because up to date only few of the estimated existing microbes have yet become cultivable [58,59]. Still the optimization of dark fermentative BHP by using wild-type microbes is an alternative.

#### *Evaluating the microbial H*_*2*_*production potential by application of* in silico *analysis*

Another highly noteworthy field related to dark fermentative BHP is the strain identification based on *in silico* analysis. The information gain is not restricted to phylogenetical knowledge, but also sequence information on enzymes is available [60,61]. Therefore, substantial information on the catalytic units for H_2_ production can be retrieved. Moreover, screening for specific enzymes in respect to substrate breakdown and utilization can also be done. Isolated information on certain microorganisms can be retrieved, but also whole genomes of several dark fermentative BHP strains are sequenced and provide full access for physiological and *in silico* analysis, offering putative modification possibilities towards metabolic engineering objectives.

#### Optimization of H_2_ production by application of metabolic engineering

Metabolic engineering is especially important for dark fermentative BHP strains that comprise high Y_(H2/S)_ and qH_2_, but whereof high volumetric production rates are either inherently limited by metabolic bottlenecks (*i.e.* organic acids, solvent and alcohol production) or, when concerning thermophilic strains, by their achievable cell densities. Usually *Escherichia coli* is the target for metabolic engineering [62-68]. Since *Clostridia* spp. show high Y_(H2/S)_ and high qH_2_ in comparison to *Escherichia* spp., metabolic strain engineering is an interesting option in order to increase HER. Nevertheless, *Escherichia* spp. can be genetically modified relatively easy in respect to their facultative anaerobic growth characteristic [64,65,67,69], hence, allowing unsophisticated achievable growth in a variety of culture vessels. *Clostridia* spp., *Caldicellulosiruptor* spp. and other strict anaerobic genera in turn require more sophisticated cultivation set-up, because they are obligate anaerobes. Nevertheless, strains of both genera have been genetically modified [64,65,67,69-72]. In this respect the application of directed evolution [73] towards optimization of cultivation conditions or the substrate utilization spectrum could be another favourable approach.

### Biochemical engineering potential for increasing H_2_ production

#### Optimization of H_2_ production by bioprocess engineering

For a robust and commercial usefully application of dark fermentative BHP several factors have to be addressed. Firstly, chemical (*i.e.* pH, ionic strength, CO_2_ solubility) and physical factors (*i.e.* temperature, partial pressure of H_2_ and CO_2_, agitation) influencing the Y_(H2/S)_ and qH_2_ need to be identified, which are usually already known and differ between various strains [28,40,74-77]. By using open cultivation systems removal of inhibitory gaseous compounds could be and is done by continuous stripping with inert gas. Secondly, factors for increasing the HER (cell retention, end product inhibition) have to be elucidated. In order to enhance the HER, an increase of the biomass concentration is required. This may be accomplished by using membrane filtration to separate unwanted metabolites and retain the biomass within the bioreactor. Hence, fed-batch cultivation for dark fermentative BHP can become a promissing approach.

The medium contains the carbon substrate for biomass and H_2_ production and is a very important starting point for optimization during bioprocess development. Many conditions, which are presented in Additional files 1, 2, 3, 4, 5, do not properly reflect the status of a pure carbon source for H_2_ production. Hence, in many experiments complex medium amendments are used. Since most of the undefined compounds undergo temporal fluctuations from lot to lot during the production process, its composition is not always consistent. Hence, the use of complex compounds does not easily allow conclusions on the influence of the carbon source on H_2_ production. In order to establish a robust bioprocess quantitative work on defined medium needs to be performed for strain characterization. This is an important consideration in order to elucidate the strains growth parameters and inherent potential of H_2_ production.

#### Use of Design of Experiments strategy for optimization of H_2_ production

Many articles have analysed the impact of the medium composition in order to increase Y_(H2/S)_, HER or qH_2_[35,78-82]. These investigations have been performed invariantly, thus by changing only one culture variable, but more and more examinations use the advantage of Design of Experiments (DoE), which has proven to be very successful [41,55,83-89]. This experimental strategy allows multivariate analysis by modification of several variables at one time, and moreover to optimize for the response(s) of interest. During the successive steps of a DoE application, optimization of BHP can be achieved by elucidation of medium components, but also on other products than H_2_, such as CO_2_, organic acids, solvents and alcohols or even other inhibitory compounds. Moreover, the influence of chemical and physical parameters on H_2_ production may be included in the investigation. Hence, a comprehensive DoE is much faster in identification of the optimal operation point, to be individually optimized for the bioprocess of interest. DoE screening and successive optimization results in an amended medium composition, identifies the corresponding culture parameters and concomitantly the optimum cultivation conditions for improved H_2_ production.

Our review shows the inherent potential and the need for quantitative investigation of pure culture dark fermentative BHP. Especially the elucidation of non-food substrates for H_2_ production is possibly of higher potential commercial applicability. From this point of view, the use of complex media for H_2_ production could rather represent a putative real case scenario. However, strain characterization is crucial and has to be performed in defined media for elucidation of the strain's full physiological potential. Herewith we propose a set of physiological scalable parameters for characterization and optimization of dark fermentative BHP strains by using bioprocessing. The first step should be a sound investigation by appication of DoE for elucidation of the following culture parameters: Y_(H2/S)_, MER and qH_2_. In a successive investigation the addition of complex or undefined medium componets should to be investigated and compared in respect to initial elucidated culture parameters. Hence, future investigations in this field of bioprocessing could be rapidly completed.

## Conclusions

This review summarizes the work done on pure and defined co-culture dark fermentative BHP since the year 1901. Qualitative growth characteristics and quantitative normalized results of H_2_ production for more than 2000 conditions are presented. Now these normalized and comparable results become available to the scientific community.

Statistically based evidence shows that thermophilic strains comprise high substrate conversion efficiency, but mesophilic strains achieve high volumetric productivity.

Microbes of *Thermoanaerobacterales* (Family III) have to be preferred when aiming to achieve a high Y_(H2/S)_ in comparison to the families *Clostridiaceae* and *Enterobacteriaceae*, based on a comprehensive statistical substantiation.

The limited number of results available on dark fermentative BHP from fed-batch cultivations indicates the yet underestimated potential of this bioprocessing application.

For an efficient bioprocess development the optimization of H_2_ production by using DoE strategy for medium modification, cultivation condition improvement and inhibitory compound analysis should be preferred and a set of physiological scalable parameters is suggested.

Comparability of key culture parameters of dark fermentative BHP is of utmost importance and thus the following entities should be used for the presentation of results: Y_(H2/S)_ [mol C-mol^-1^], HER [mmol L^-1^ h^-1^] and qH_2_ [mmol g^-1^ h^-1^].

## Abbreviations

BHP, Biological hydrogen production; CH4, Methane; C-mol, Moles of carbon; CO2, Carbon dioxide; H2, Molecular hydrogen; H2O, Water; HER, Volumetric hydrogen production rate; qH2, Specific hydrogen production rate; Y(H2/S), Moles of hydrogen produced per moles of substrate consumed.

## Competing interests

The authors declare that they have no competing interests.

## Authors’ contributions

SR reviewed the literature on dark fermentative biohydrogen production, prepared the tables, figures, additional files, performed the statistical analysis, drafted the manuscript and coordinated the review. SR and CH contributed to the conception and design of the manuscript. CH helped to draft the manuscript. All authors have read and approve the final version of the manuscript.

## Supplementary Material

Additional file 1 ** Strains reported to produce biohydrogen without the possibility to calculate or retrieve quantitative results**[3,4,6,7,48,80,82,90-204]. Click here for file

Additional file 2 ** Closed batch dark fermentative biohydrogen production**[4,29,35,39,42,53-55,64-67,69,78,82,83,85,86,98,120,130,131,137,142,166,180,182-184,192,194,198,205-314]. Click here for file

Additional file 3 ** Batch dark fermentative biohydrogen production**[28,32,33,45,49,56,57,62,63,65,66,72,76,78,79,81,84,90,96,110,115,315-369]. Click here for file

Additional file 4 ** Chemostat culture dark fermentative biohydrogen production**[29,34,46,47,56,75,77,96,118,122,124,125,228,257-260,279,286,291,292,331,353,356,370-400]. Click here for file

Additional file 5 ** Fed-batch dark fermentative biohydrogen production**[38]. Click here for file
