# Supplementary material for: A comprehensive and quantitative review of dark fermentative biohydrogen production
Source: Microb Cell Fact. 2012 Aug 27;11:115. doi: 10.1186/1475-2859-11-115 (PMC3443015; doi:10.1186/1475-2859-11-115)
Supplement: Additional file 1 — Strains reported to produce biohydrogen without the possibility to calculate or retrieve quantitative results[3,4,6,7,48,80,82,90-204]. [file 1475-2859-11-115-S1.pdf]

Additional file 1: Strains reported to produce biohydrogen without the possibility to calculate or retrieve quantitative results

Genus	Species	Strain	Temperature [°C]	pH	Substrate	Reference[+]
Alcaligenes	*eutrophus*	DSM 1348	30	6.0	gluconate	[90]
Alcaligenes	*eutrophus*	DSM 428	30	6.0	gluconate	[90]
Alkalibacter	*saccharofermentans*	DSM 14828	35	9.0	glucose	[91]
Aminobacterium	*colombiense*	DSM 12261	37	7.3	alanine	[92]
Anaerobranca	*horikoshii*	DSM 9786	57	8.5	yeast extract	[93]
Anaerobranca	*horikoshii*	DSM 9786	57	8.5	yeast extract/fumarate	[93]
Anaerosinus	*glycinii*	DSM 5192	30		glycerol	[94]
Anaerovibrio	*lipolytica*	L 1342			glycerol	[95]
Anaerovibrio	*lipolytica*	L 1641			glycerol	[95]
Anaerovibrio	*lipolytica*	L 1741			glycerol	[95]
Bacillus	*licheniformis*	JK1	38-40	6	wheat grain slurry	[96]
Bacillus	*licheniformis*	JK1	38-40	7	wheat grain slurry	[96]
Bacillus	*licheniformis*	JK1	38-40	8	wheat grain slurry	[96]
Bacillus	*subtilis*	VC2	37	7	wheat grains	[97]
Bacillus	*licheniformis*	JK1	37	7	wheat grains	[97]
Bacteroides	*eggerthii*	ATCC 27754	37	7.0	glucose	[98]
Bacteroides	*fragilis*	ATCC 25285	37	7.0	glucose	[98]
Caldicellulosiruptor	*acetigenus*	DSM 7040	65-68	7.0	glucose	[99]
Caldicellulosiruptor	*kristjanssonii*	DSM 12137	78	7.0	cellobiose	[100]
Caldicellulosiruptor	*owensensis*	ATCC 700167	75	7.5	glucose	[101]
Caldicellulosiruptor	*saccharolyticus*	DSM 8903	70		glucose	[48]
Caldicellulosiruptor	*saccharolyticus*	DSM 8903	70		glucose/filter paper	[48]
Caldicellulosiruptor	*saccharolyticus*	DSM 8903	70		fructose	[48]
Caldicellulosiruptor	*saccharolyticus*	DSM 8903	70		fructose/filter paper	[48]
Caldicellulosiruptor	*saccharolyticus*	DSM 8903	55		corn leaves	[48]
Caldicellulosiruptor	*saccharolyticus*	DSM 8903	70		sucrose	[102]

Caldicellulosiruptor	*saccharolyticus*	DSM 8903	70	7.0	barley straw	[103]
Caldicellulosiruptor	*saccharolyticus*	DSM 8903	70	7.0	corn stalk	[103]
Caldicellulosiruptor	*saccharolyticus*	DSM 8903	70	7.0	barley grains	[103]
Caldicellulosiruptor	*saccharolyticus*	DSM 8903	70	7.0	corn grains	[103]
Caldicellulosiruptor	*saccharolyticus*	DSM 8903	70	7.0	sugar beet roots	[103]
Caldicellulosiruptor	*saccharolyticus*	DSM 8903	70	7.2	paper sludge hydrolysate	[104]
Citrobacter	*freundii*	TIT0101	30	7	glucose	[105]
Clostridium	*acetobutylicum*	ATCC 39236	34-46		Maltrin	[106]
Clostridium	*acidtolerans*	DSM 17425		6.0		[107]
Clostridium	*aldrichii*	ATCC 49358	35	7	cellobiose	[108]
Clostridium	*algoriphilum*	DSM 16153	6	6.8-7.2	glucose	[109]
Clostridium	*amygdalinum*	C9	37	7.5	xylan	[110]
Clostridium	*amygdalinum*	C9	37	7.5	starch	[110]
Clostridium	*beijerinckii*	ATCC 51743	37	6.0	anthraquinone-2.6-disulfonate	[111]
Clostridium	*beijerinckii*	ATCC 51743	37	6.0	humic acids	[111]
Clostridium	*beijerinckii*	DSM 791		6.0		[107]
Clostridium	*beijerinckii*	Fanp3	36	6.5	CMC	[82]
Clostridium	*beijerinckii*	Fanp3	36	6.5	soluble starch	[82]
Clostridium	*beijerinckii*	Fanp3	36	6.5	cellulose	[82]
Clostridium	*bifermentans*		35	6.2	waste sludge	[112]
Clostridium	*bifermentans*		35	6.4	wastewater sludge	[113]
Clostridium	*butyricum*	AS1.209	35	7.2	steam exploded corn straw	[114]
Clostridium	*butyricum*	AS1.209	35	7.2	raw corn straw	[114]
Clostridium	*butyricum*	CGS2	37	7.5	cellulose	[80]
Clostridium	*butyricum*	CGS5	37	7.5	sucrose	[115]
Clostridium	*butyricum*	CGS5	37	7.2	sucrose	[116]
Clostridium	*butyricum*	CGS5	37	7.5	xylan	[117]
Clostridium	*butyricum*	CGS5	37	7.5	sugarcane bargasse hydrolysate	[117]
Clostridium	*butyricum*	DSM 10702		6.0		[107]

Clostridium	*butyricum*	DSM 5431	33	7.0	glycerol	[118]
Clostridium	*butyricum*	IFO 3847	30	7.9	glutmate/lactate	[119]
Clostridium	*butyricum*	IFO 3847	37	7.7	glucose	[120]
Clostridium	*butyricum*	IFO 3847	37	6.2	wastewater	[121]
Clostridium	*butyricum*	IFO 13949	36	6.5	starch	[122]
Clostridium	*butyricum*	LMD 77-11	30	6.0	glucose	[123]
Clostridium	*butyricum*	LMG 1212 t2	37	6.8	glycerol	[124]
Clostridium	*butyricum*	LMG 1212 t2	37	6.8	glycerol/acetate	[124]
Clostridium	*butyricum*	LMG 1213 t1	37		mannitol	[125]
Clostridium	*butyricum*	LMG 1213 t1	37		mannitol/acetate	[125]
Clostridium	*butyricum*	LMG 1213 t1	37		mannitol/butyrate	[125]
Clostridium	*butyricum*	LMG 1213 t1	37		mannitol/formate	[125]
Clostridium	*butyricum*	LMG 1213 t1	37		mannitol/fumarate	[125]
Clostridium	*butyricum*	LMG 1213 t1	37		mannitol/dihydroacetone	[125]
Clostridium	*butyricum*	LMG 1213 t1	37		mannitol/pyruvate	[125]
Clostridium	*butyricum*	LMG 1213 t1	37		mannitol/glucose	[125]
Clostridium	*butyricum*	NCIB 9576	37		starch	[126]
Clostridium	*butyricum*	NCIB 9576	37		algal biomass	[126]
Clostridium	*butyricum*	NCIB 9576	37		commercial *Chlorella* powder	[126]
Clostridium	*caminithermale*	DSM 15212	45	6.6	glucose	[127]
Clostridium	*cellobioparus*		38	7.4	glucose	[128]
Clostridium	*cellobioparus*		38	7.4	cellulose	[128]
Clostridium	*cellulolyticum*	ATCC 35319	34	7.8	cellulose	[129]
Clostridium	*diolis*	DSM 15410		6.0		[107]
Clostridium	*homoproprionicum*	LuHBu1	37	7.2	pyruvate	[130]
Clostridium	*hungatei*	B3B	30	7.2	cellulose	[131]
Clostridium	*kluyveri*		33	7.4-7.5	ethanol/acetate	[132]
Clostridium	*kluyveri*		33	7.4-7.5	crotonate	[132]
Clostridium	*longisporum*	B6405	35-45		alfalfa cell walls	[133]

Clostridium	*longisporum*	OC4	35-45		alfalfa cell walls	[133]
Clostridium	*lortetti*	ATCC 35059	37	6.5	glucose	[134]
Clostridium	*ludense*	DSM 17049		6.0		[107]
Clostridium	*methoxybenzovorans*	DSM 12182	37	7.4	sucrose	[135]
Clostridium	*methylpentosum*	ATCC 43829	37	6.5	glucose	[136]
Clostridium	*pasteurianum*	CH1	37	7.5	cellulose	[80]
Clostridium	*pasteurianum*	CH4	37	7.5	cellulose	[80]
Clostridium	*pasteurianum*	CH5	37	7.5	cellulose	[80]
Clostridium	*pasteurianum*	CH7	37	7.5	cellulose	[80]
Clostridium	*pasteurianum*	DSM 525		6.0		[107]
Clostridium	*pasteurianum*	LMG 8285	37	6.8	glycerol	[124]
Clostridium	*pasteurianum*	LMG 8285	37	6.8	glycerol/acetate	[124]
Clostridium	*peptidivorans*	DSM 12505	37	7	isoleucine	[137]
Clostridium	*peptidivorans*	DSM 12505	37	7	serine	[137]
Clostridium	*peptidivorans*	DSM 12505	37	7	casamino acid	[137]
Clostridium	*phytofermentans*	ATCC 700394	37	7	cellulose	[138]
Clostridium	*phytofermentans*	ATCC 700394	37	7	cellobiose	[138]
Clostridium	*puniceum*	DSM 2619		6.0		[107]
Clostridium	*saccharoperbutylacetonicum*	DSM 14923		6.0		[107]
Clostridium	sp.	A22	34	7.8	cellulose	[129]
Clostridium	sp.	BL-17		6.0		[107]
Clostridium	sp.	BL-20		6.0		[107]
Clostridium	sp.	BL-21		6.0		[107]
Clostridium	sp.	BL-22		6.0		[107]
Clostridium	sp.	BL-26		6.0		[107]
Clostridium	sp.	BL-28		6.0		[107]
Clostridium	sp.	BL-3		6.0		[107]
Clostridium	sp.	BL-3		7.2		[107]
Clostridium	sp.	BL-30		6.0		[107]

Clostridium	sp.	BL-8		6.0		[107]
Clostridium	sp.	EG3	37	6.8-7.2	glucose	[139]
Clostridium	*thermocellum*	AS-39	60	7.3	cellulose	[140]
Clostridium	*thermocellum*	AS-39	60	7.3	cellobiose	[140]
Clostridium	*thermocellum*	AS-39	60	7.3	cellulose	[140]
Clostridium	*thermocellum*	AS-39	60	7.3	cellobiose	[140]
Clostridium	*thermocellum*	DMS 123	55		tissue paper	[141]
Clostridium	*thermocellum*	DMS 123	55		tissue paper	[141]
Clostridium	*thermocellum*	DMS 123	55		tissue paper	[141]
Clostridium	*thermocellum*	JN4	60		corn stalk powder	[142]
Clostridium	*thermocellum*	JN4	60		corn cob powder	[142]
Clostridium	*thermocellum*	JN4	60		corn stalk powder	[142]
Clostridium	*thermocellum*	JN4	60		corn cob powder	[142]
Clostridium	*thermocellum*	JW20	60		cellobiose	[143]
Clostridium	*thermocellum*	LQRI	60		glucose	[144]
Clostridium	*thermocellum*	LQRI	60		cellobiose	[144]
Clostridium	*thermocellum*	LQRI	60	7.3	cellulose	[140, 144]
Clostridium	*thermocellum*	LQRI	60	7.3	cellobiose	[140]
Clostridium	*thermocellum*	YS	60	7.3	cellulose	[140]
Clostridium	*thermocellum*	YS	60	7.3	cellobiose	[140]
Clostridium	*thermohydrosulfuricum*	E100-69	70		glucose	[145]
Clostridium	*thermohydrosulfuricum*	E39	65		xylose	[144]
Clostridium	*thermohydrosulfuricum*	E39	65		glucose	[144]
Clostridium	*thermohydrosulfuricum*	E39	65		cellobiose	[144]
Clostridium	*thermohydrosulfuricum*	JW102	70		glucose	[145]
Clostridium	*thiosulfatireducens*	DSM 13105		7.2		[107]
Clostridium	*tryobutyricum*	ATCC 25755	37	5.3	xylose	[146]
Clostridium	*tryobutyricum*	ATCC 25755	37	6.3	xylose	[146]
Clostridium	*xylanovorans*	DSM 12503	37		glucose	[147]

Coprothermobacter	*platensis*	DSM 11748	55	7.0	gelatin	[148]
Coprothermobacter	*proteolyticus*	DSM 5265	63	7.5	glucose	[148]
Desulfovibrio	*vulgaris*	Madison	30		pyruvate	[149]
Desulfovibrio	*vulgaris*	Madison	30		lactate	[149]
Desulfovibrio	*vulgaris*	H801	28		lactate	[150]
Desulfovibrio	*vulgaris*	H801	28		pyruvate	[150]
Desulfovibrio	*vulgaris*	Hyd100	28		lactate	[150]
Desulfovibrio	*vulgaris*	NCIMB 8303	28		lactate	[150]
Dethiosulfovibrio	*marinus*	DSM 12537	28	6.5-7	glucose	[151]
Dethiosulfovibrio	*russensis*	DSM 12538	28	6.5-7	glucose	[151]
Escherichia	*coli*	AN346	37	7.4	glucose	[152]
Escherichia	*coli*	AN817	37	7.4	glucose	[152]
Escherichia	*coli*	B1LK0	30	7.3	glucose	[153]
Escherichia	*coli*	BL21(DE3) Δ*tonA*	37		glucose	[154]
Escherichia	*coli*	BL21(DE3)pSynhox ΔH_2ase	37		glucose	[155]
Escherichia	*coli*	C600	30	7	glucose	[105]
Escherichia	*coli*	C600	30	7	glucose	[105]
Escherichia	*coli*	CECT 99	25		glucose	[156]
Escherichia	*coli*	communis	37	6	formate	[157]
Escherichia	*coli*	communis			glucose	[3, 4]
Escherichia	*coli*	communis			mannitol	[3]
Escherichia	*coli*	communis			fructose	[3]
Escherichia	*coli*	communis			galactose	[3]
Escherichia	*coli*	communis			arabinose	[3]
Escherichia	*coli*	communis			glucose/formate	[4]
Escherichia	*coli*	DADE	30	7.3	glucose	[153]
Escherichia	*coli*	FM420	37	7.4	glucose	[152]
Escherichia	*coli*	FM911	37	7.5	glucose	[158]
Escherichia	*coli*	FM911	37	5.5	glucose/formate	[158]

Escherichia	*coli*	FRAG115	37	7.5	glucose	[158]
Escherichia	*coli*	FRAG115	37	7.5	glucose/formate	[158]
Escherichia	*coli*	FRAG115	37	5.5	glucose/formate	[158]
Escherichia	*coli*	FRAG90		6.5		[159]
Escherichia	*coli*	FRAG90		7.5		[159]
Escherichia	*coli*	FRAG90	37	7.4	glucose	[152]
Escherichia	*coli*	FTD701	30	7.3	glucose	[153]
Escherichia	*coli*	HD700	37	7.5	glucose	[158]
Escherichia	*coli*	HD700	37	5.5	glucose	[158]
Escherichia	*coli*	HD700	37	7.5	glucose/formate	[158]
Escherichia	*coli*	HD700	37	5.5	glucose/formate	[158]
Escherichia	*coli*	HD701	30	7.3	glucose	[153]
Escherichia	*coli*	HD705	37	7.5	glucose	[158]
Escherichia	*coli*	HD705	37	5.5	glucose	[158]
Escherichia	*coli*	HD705	37	7.5	glucose/formate	[158]
Escherichia	*coli*	HD705	37	5.5	glucose/formate	[158]
Escherichia	*coli*	HD706		6.5		[159]
Escherichia	*coli*	HD706		7.5		[159]
Escherichia	*coli*	HD707		6.5		[159]
Escherichia	*coli*	HD707		7.5		[159]
Escherichia	*coli*	HK-8(pCBH4)	30	7	glucose	[105]
Escherichia	*coli*	HK-8(pCBH4)	30	7	glucose	[105]
Escherichia	*coli*	JRG3615		6.5		[159]
Escherichia	*coli*	JRG3615		7.5		[159]
Escherichia	*coli*	JRG3615	37	7.5	glucose	[158]
Escherichia	*coli*	JRG3615	37	5.5	glucose	[158]
Escherichia	*coli*	JRG3615	37	7.5	glucose/formate	[158]
Escherichia	*coli*	JRG3615	37	5.5	glucose/formate	[158]
Escherichia	*coli*	JRG3618		6.5		[159]

Escherichia	*coli*	JRG3618		6.5		[159]
Escherichia	*coli*	JRG3618		7.5		[159]
Escherichia	*coli*	JRG3621		6.5		[159]
Escherichia	*coli*	JRG3621		6.5		[159]
Escherichia	*coli*	JRG3621		7.5		[159]
Escherichia	*coli*	JRG3933	37	7.5	glucose	[158]
Escherichia	*coli*	JRG3933	37	5.5	glucose	[158]
Escherichia	*coli*	JRG3933	37	7.5	glucose/formate	[158]
Escherichia	*coli*	JRG3933	37	5.5	glucose/formate	[158]
Escherichia	*coli*	K12 (lambda)	37	7.3-7.4	glucose	[160]
Escherichia	*coli*	K12 (lambda)	37	7.3-7.4	maltose	[160]
Escherichia	*coli*	MC4100		6.5		[159]
Escherichia	*coli*	MC4100		7.5		[159]
Escherichia	*coli*	MC4100	37	7.4	glucose	[152]
Escherichia	*coli*	MC4100	30	7.3	glucose	[153]
Escherichia	*coli*	MC4100	37	7.5	glucose	[158]
Escherichia	*coli*	MC4100	37	5.5	glucose	[158]
Escherichia	*coli*	MC4100	37	7.5	glucose/formate	[158]
Escherichia	*coli*	MC4100	37	5.5	glucose/formate	[158]
Escherichia	*coli*	S13	37	7.2	formate	[161]
Escherichia	*coli*	S13	37	7.2	formate	[161]
Escherichia	*coli*	S13	37	7.2	glucose	[161]
Escherichia	*coli*	TG1	37			[67]
Escherichia	*coli*	TK2242	37	7.4	glucose	[162]
Enterobacter	*aerogenes*	HO-39	36	6.5	starch	[122]
Ethanoligenens	*harbinense*	JCM12961	35	4.5-5.0	glucose	[163]
Ethanoligenens	*harbinense*	X-29	35	4.5-5.0	glucose	[163]
Fervidobacterium	*gondwanense*	ACM 5017	65-68	6.8	glucose	[164]
Fervidobacterium	*pennavorans*	DSM 9078	65			[165]

Geobacter	*hydrogenophilus*	H-2	30		acetate	[166]
Geobacter	*hydrogenophilus*	H-2	30		butyrate	[166]
Geobacter	*hydrogenophilus*	H-2	30		benzoate	[166]
Geobacter	*hydrogenophilus*	H-2	30		ethanol	[166]
Geobacter	*metallireducens*	GS15	30		acetate	[166]
Gluconacetobacter	*diazotrophicus*	ATCC 49037	30	7.4	glucose	[167]
Gluconacetobacter	*diazotrophicus*	T2	30	7.4	glucose	[167]
Hafnia	*alvei*	CECT 400	39		glucose	[156]
Haloanaerobium	*hydrogeniformans*		33	11		[168]
Haloanaerobium	*lacusroseus*	DSM 10165	40	7.0	glucose	[169]
Haloincola	*saccharolytica*	DSM 7379	40	7.0	glucose	[170]
Halonatronum	*saccharophilum*	DSM 13868			glucose	[91]
Halothermothrix	*orenii*	H168	60	6.5-7.0	glucose	[171]
Ilyobacter	*insuetus*	DSM 6831	30	6.5-8.0	quinic acid or shikimic acid	[172]
Klebsiella	*oxytoca*	HP1	37.5	7.0	hydrolyzed bagasse	[173]
Klebsiella	*oxytoca*	HP1 Δ*ahdE*	37.5	7.0	hydrolyzed bagasse	[173]
Klebsiella	*pneumoniae*	ECU-15			glucose	[174]
Pantoea	sp.	C9	30	7.4	glucose	[167]
Pelobacter	*carbinolicus*	DSM 2380	35	6.5-7.2	butanediol	[175]
Pelobacter	*carbinolicus*	DSM 2380	35	6.5-7.2	ethanol	[175]
Petrotoga	*miotherma*	DSM 10691	55		DSM 718	[165]
Pseudomonas	sp.	GZ1	35		wastewater sludge	[176]
Roseburia	*intestinalis*	XB6B4	37			[177]
Ruminococcus	*albus*	43-1	39		cellulose	[178]
Ruminococcus	*albus*	46-1	39		cellulose	[178]
Ruminococcus	*albus*	56-2	39		cellulose	[178]
Ruminococcus	*albus*	69	39		cellulose	[178]
Ruminococcus	*flavefaciens*	61-1	39		cellulose	[178]
Ruminococcus	*flavefaciens*	S1-2	39		cellulose	[178]

Ruminococcus	*flavefaciens*	S1-y	39		cellulose	[178]
Ruminococcus	sp.	19.08.6C	38		cellobiose	[179]
Ruminococcus	sp.	20	38		cellobiose	[179]
Ruminococcus	sp.	21.09.6A	38		cellobiose	[179]
Ruminococcus	sp.	21.09.6B	38		cellobiose	[179]
Ruminococcus	sp.	21.09.6E	38		cellobiose	[179]
Ruminococcus	sp.	21.09.6F	38		cellobiose	[179]
Ruminococcus	sp.	21.09.6G	38		cellobiose	[179]
Ruminococcus	sp.	22.08.6A	38		cellobiose	[179]
Ruminococcus	sp.	22.08.6B	38		cellobiose	[179]
Ruminococcus	sp.	22.08.6F	38		cellobiose	[179]
Ruminococcus	sp.	7	38		cellobiose	[179]
Ruminococcus	sp.	c94	38		cellobiose	[179]
Ruminococcus	sp.	FD1	38		cellobiose	[179]
Sarcina	*ventriculi*	JK		2-9	glucose	[180]
Selenomonas	*acidaminovorans*	DSM 6589	55	6.5-8.1	glutamate	[181]
Selenomonas	*acidaminovorans*	DSM 6589	55	6.5-8.1	ketoglutarate	[181]
Selenomonas	*acidaminovorans*	DSM 6589	55	6.5-8.1	histidine	[181]
Selenomonas	*acidaminovorans*	DSM 6589	55	6.5-8.1	arginine	[181]
Selenomonas	*acidaminovorans*	DSM 6589	55	6.5-8.1	ornithine	[181]
Selenomonas	*acidaminovorans*	DSM 6589	55	6.5-8.1	glucose	[181]
Selenomonas	*acidaminovorans*	DSM 6589	55	6.5-8.1	fructose	[181]
Selenomonas	*acidaminovorans*	DSM 6589	55	6.5-8.1	alanine	[181]
Selenomonas	*acidaminovorans*	DSM 6589	55	6.5-8.1	serine	[181]
Selenomonas	*acidaminovorans*	DSM 6589	55	6.5-8.1	pyruvate	[181]
Selenomonas	*acidaminovorans*	DSM 6589	55	6.5-8.1	glycerol	[181]
Selenomonas	*acidaminovorans*	DSM 6589	55	6.5-8.1	citrate	[181]
Selenomonas	*acidaminovorans*	DSM 6589	55	6.5-8.1	isoleucine	[181]
Selenomonas	*acidaminovorans*	DSM 6589	55	6.5-8.1	valine	[181]

Selenomonas	*acidaminovorans*	DSM 6589	55	6.5-8.1	leucine	[181]
Selenomonas	*ruminantium*	GA192	37		glucose	[182]
Selenomonas	*ruminantium*	HD4	37		glucose	[183]
Shewanella	*alga*	BRY	20		lactate	[166]
Shewanella	*palmitatis*	SDBY	37		lactate	[166]
Spirochaeta	*aurantia*	J1	30	7.5	maltose	[184]
Syntrophothermus	*lipocalidus*		55	6.2	butyrate	[92]
Tepidibacter	*thalassicus*	DSM 15285	50	6.5-6.8	glucose	[185]
Thermoanaerobacter	*brockii*	DSM 1457	70	7.5	glucose	[186, 187]
Thermoanaerobacter	*brockii*	HDT1	65		glucose	[186]
Thermoanaerobacter	*brockii*	HDT6	65		glucose	[186]
Thermoanaerobacter	*brockii*	DSM 9801	55-60	7.3	glucose	[188]
Thermoanaerobacter	*siderophilus*	DSM 12299	69-71	6.3-6.5	glucose	[189]
Thermoanaerobacter	*thermohydrosulfuricus*	DSM 567	67-69	6.9-7.5		[187]
Thermoanaerobacterium	*aciditolerans*	DSM 16487	55	5.7	sucrose	[190]
Thermoanaerobacterium	*polysaccharolyticum*	DSM 13641	65-68	6.8-7.0	glucose	[191]
Thermoanaerobacterium	*saccharolyticum*	DSM 7060	60	6.0	glucose	[187]
Thermoanaerobacterium	*saccharolyticum*	DSM 7060	60	6.0	xylan	[187]
Thermoanaerobacterium	*saccharolyticum*	DSM 7060	60	6.0	rhamnose	[187]
Thermoanaerobacterium	*thermosulfurigenes*	DSM 2229	60	5.5-6.5	glucose	[187]
Thermoanaerobacterium	*xylanolyticum*	DSM 7097	60	6.0		[187]
Thermoanaerobacterium	*zeae*	DSM 13642	65-70	3.9-7.9	glucose	[191]
Thermoanaerobium	*brockii*	HTD4	65		glucose	[192]
Thermoanaerobium	*brockii*	HTD4	65		starch	[192]
Thermoanaerobium	*brockii*	HTD4	65		acetone	[192]
Thermoanaerobium	*brockii*	HTD4	65		glucose/acetone	[192]
Thermoanaerobium	*brockii*	HTD4	65		pyruvate/acetone	[192]
Thermobrachium	*celere*	DSM 8682	67	8.2	glucose	[193]
Thermobrachium	*celere*	DSM 8682	67	8.2	yeast extract	[194]

Thermobrachium	*celere*	DSM 8682	67	8.2	tryptone	[194]
Thermobrachium	*celere*	DSM 8682	67	8.2	glucose/yeast extract	[194]
Thermobrachium	*celere*	DSM 8682	67	8.2	glucose/tryptone	[194]
Thermococcus	sp.	ES-1	81		yeast extract/tryptone	[195]
Thermodesulfobacterium	*commune*	YSRA	60		pyruvate	[149]
Thermodesulfobacterium	*commune*	YSRA	60		lactate	[149]
Thermosipho	*africanus*	DSM 5309	75			[165]
Thermotoga	*elfii*	DSM 9442	65	8	glucose	[165, 196]
Thermotoga	*elfii*	DSM 9442	65	8	xylose/glucose	[196]
Thermotoga	*elfii*	DSM 9442	65	7.2	paper sludge hydrolysate	[104]
Thermotoga	*lettingae*	DSM 14385	65	7.0	glucose	[197]
Thermotoga	*maritima*	DSM 3109	80	6.5-7.0	starch	[198]
Thermotoga	*maritima*	DSM 3109	80	6.5-7.0	cellulose	[198]
Thermotoga	*maritima*	DSM 3109	80	6.5-7.0	carboxymethyl cellulose	[198]
Thermotoga	*neapolitana*	ATCC 49049	70			[165]
Thermotoga	*neapolitana*	DSM 4359	75	6.5-7.0	starch	[198]
Thermotoga	*neapolitana*	DSM 4359	75	6.5-7.0	cellulose	[198]
Thermotoga	*neapolitana*	DSM 4359	75	6.5-7.0	carboxymethyl cellulose	[198]
Thermovenabulum	*ferriorganovorum*	DSM 14006	63-65	6.7-6.9	melibiose	[199]
Vibrio	*aerogenes*	ATCC 700797	30	7	glucose	[200]
		66-1	39		cellulose	[178]
		B2	37		microcrystalline cellulose	[201]
		C3	37		microcrystalline cellulose	[201]
		FMHBu1	28	7	pyruvate	[130]
		Mfc52	30	6.7	glucose	[202]
Escherichia<br>*Citrobacter*	*coli*<br>*freundii*	ATCC 6750	37	5.5	glucose	[203]
Escherichia<br>*Citrobacter*	*coli*<br>*freundii*	ATCC 6750	37	5.5	flter paper	[203]
Clostridium<br>*Thermoanaerobacterium*	*thermocellum*<br>*thermosaccharolyticum*	JN4<br>GD17	60		0.5% (w/v) corn stalk powder	[142]

Clostridium<br>*Thermoanaerobacterium*	*thermocellum*<br>*thermosaccharolyticum*	JN4<br>GD17	60		0.5% (w/v) corn cob powder	[142]
Clostridium<br>*Thermoanaerobacterium*	*thermocellum*<br>*thermosaccharolyticum*	JN4<br>GD17	60		1% (w/v) corn stalk powder	[142]
Clostridium<br>*Thermoanaerobacterium*	*thermocellum*<br>*thermosaccharolyticum*	JN4<br>GD17	60		1% (w/v) corn cob powder	[142]
Clostridium<br>*Clostridium*	thermocellum<br>thermopalmarium	DSM 1237<br>DSM 5974	55	7.0	cellulose	[204]
Clostridium<br>*Enterobacter*	*butyricum*<br>*aerogenes*	IFO 13949<br>HO-39	36	6.5	starch, argon gas	[122]
Clostridium<br>*Enterobacter*	*butyricum*<br>*aerogenes*	IFO 13949<br>HO-39	36	6.5	starch	[122]

[+] for reference please refer to manuscript
