# Supplementary material for: A comprehensive and quantitative review of dark fermentative biohydrogen production
Source: Microb Cell Fact. 2012 Aug 27;11:115. doi: 10.1186/1475-2859-11-115 (PMC3443015; doi:10.1186/1475-2859-11-115)
Supplement: Additional file 2 — Closed batch dark fermentative biohydrogen production[4,29,35,39,42,53-55,64-67,69,78,82,83,85,86,98,120,130,131,137,142,166,180,182-184,192,194,198,205-314]. [file 1475-2859-11-115-S2.pdf]

Additional file 2: Closed batch dark fermentative biohydrogen production

Genus	Species	Strain	$Y_{(H2/S)}$ [mol mol^{-1}]	qH_2 [mmol g^{-1} h^{-1}]	HER [mmol L^{-1} h^{-1}]	Temperature [°C]	pH	Main substrate and discriminative condition	Reference+
Acetobacterium	*woodii*		0.06					fructose (with Ni)	[205]
Acetobacterium	*woodii*		2.94					fructose (without Ni)	[205]
Acidaminococcus	*fermentans*	AO	0.6			39	6.7	trans-aconitate	[206]
Acidaminococcus	*fermentans*	AO	1			39	6.7	citrate	[206]
Acidaminococcus	*fermentans*	DSM 20731	0.1			37	7.5	glutamate	[207]
Acidaminococcus	*fermentans*	DSM 20731	0.89			37	7.5	trans-aconitate	[208]
Bacillus	*megaterium*	ATCC 15374			0.84	35		5 g/L glucose	[209]
Bacillus	*megaterium*	ATCC 15374			1.17	35		10 g/L glucose	[209]
Bacillus	*megaterium*	ATCC 15374			0.98	35		15 g/L glucose	[209]
Bacillus	*megaterium*	ATCC 15374			1.23	35		20 g/L glucose	[209]
Bacteroides	*fragilis*	ATCC 25285	0.01		5.56	37	7.0	glucose (with vitamin B12)	[98]
Bacteroides	*fragilis*	ATCC 25285	0.09		50	37	7.0	glucose (without vitamin B12)	[98]
Bacteroides	*splanchincus*	VPI 6842	0.10		55.6	37	7.0	glucose (with heme, without vitamin B12)	[98]
Bacteroides	*splanchincus*	VPI 6842	0.14			37	7.0	glucose (with heme)	[98]
Bacteroides	*splanchincus*	VPI 6842	0.14		77.8	37	7.0	glucose (with heme, with vitamin B12)	[98]
Bacteroides	*splanchincus*	VPI 6842	0.91			37	7.0	glucose (without heme)	[98]
Caldicellulosiruptor	*saccharolyticus*	DSM 8903	0.48			70	7.2	silphium leaves	[35]
Caldicellulosiruptor	*saccharolyticus*	DSM 8903	1.75			70	7.2	sweet shorgum plant	[35]
Caldicellulosiruptor	*saccharolyticus*	DSM 8903	1.80			70	7.2	untreated maize leaves	[35]
Caldicellulosiruptor	*saccharolyticus*	DSM 8903	2.30			70	7.2	bagasse	[35]
Caldicellulosiruptor	*saccharolyticus*	DSM 8903	2.63			70	7.2	vacuum evaporated sweet shorghum juice	[35]
Caldicellulosiruptor	*saccharolyticus*	DSM 8903	2.98			70	7.2	sweet shorgum juice	[35]
Caldicellulosiruptor	*saccharolyticus*	DSM 8903	3.67			70	7.2	pretrated maize leaves	[35]
Caldicellulosiruptor	*saccharolyticus*	DSM 8903	3.80			70	7.2	wheat straw	[35]

Caloramator	*viterbensis*	DSM 13723	0.2			60	6	glucose	[210]
Caloramator	*viterbensis*	DSM 13723	0.401			60	6	glycerol	[210]
Citrobacter	*amalonaticus*	Y19	1.12	11.26		30		5 g/L glucose	[211]
Citrobacter	*amalonaticus*	Y19	1.12	10.3		30		9 g/L glucose	[211]
Citrobacter	*amalonaticus*	Y19	1.24	15.71		30		1.5 g/L glucose	[211]
Citrobacter	*amalonaticus*	Y19		25.8		30		formate	[212]
Citrobacter	*amalonaticus*	Y19		6.6		30		pyruvate	[212]
Citrobacter	*amalonaticus*	Y19		19.5		30		methylvirologen	[212]
Citrobacter	*amalonaticus*	Y19		27		30		benzylvirologen	[212]
Citrobacter	sp.	Y19	1.05	32.3		36	6.0-7.0	10% (w/v) glucose	[213]
Citrobacter	sp.	Y19	2.49	32.3		36	6.0-7.0	1% (w/v) glucose	[213]
Clostridium	*acetobutylicum*	ATCC 4259	1.178			34	5.4	glucose (carbon monoxide in headspace)	[214]
Clostridium	*acetobutylicum*	ATCC 4259	1.391			34	5.4	glucose	[214]
Clostridium	*acetobutylicum*	ATCC 824	2.3			35	6.5	cellobiose	[215]
Clostridium	*acetobutylicum*	IAM 190011			0.83	37	7	glucose	[120]
Clostridium	*acetobutylicum*	IAM 190012			0.5	37	7	glucose	[120]
Clostridium	*acetobutylicum*	NCIMB 13357	3.28		6.64	30	7.0	glucose (no CO_2 scavenging)	[216]
Clostridium	*acetobutylicum*	NCIMB 13357	3.39		3.92	30	7.0	glucose (CO_2 scavenging)	[216]
Clostridium	*acetobutylicum*	X9		6.4		37	5.0	microcrystalline cellulose	[201]
Clostridium	*beijerinckii*	ATCC 25752			0.69	35		5 g/L glucose	[217]
Clostridium	*beijerinckii*	ATCC 25752			1.08	35		10 g/L glucose	[217]
Clostridium	*beijerinckii*	ATCC 25752			1.48	35		15 g/L glucose	[217]
Clostridium	*beijerinckii*	ATCC 25752			1.3	35		20 g/L glucose	[217]
Clostridium	*beijerinckii*	Fanp3	1.26		12.3	36	6.5	xylose	[82]
Clostridium	*beijerinckii*	Fanp3	1.27		6.4	36	6.5	galactose	[82]
Clostridium	*beijerinckii*	Fanp3	1.55		22	36	6.5	mannose	[82]
Clostridium	*beijerinckii*	Fanp3	1.72		27.3	36	6.5	fructose	[82]

Clostridium	*beijerinckii*	Fanp3	1.86		27	36	6.5	glucose	[82]
Clostridium	*beijerinckii*	Fanp3	2.03		24.8	35	6.47	glucose	[82]
Clostridium	*beijerinckii*	Fanp3	2.58		10.2	36	6.5	lactose	[82]
Clostridium	*beijerinckii*	Fanp3	3.72		22	36	6.5	maltose	[82]
Clostridium	*beijerinckii*	Fanp3	3.9		27.5	36	6.5	cellobiose	[82]
Clostridium	*beijerinckii*	Fanp3	4.20		22.1	36	6.5	sucrose	[82]
Clostridium	*beijerinckii*	Fanp3			34.3	36	6.5	dextrin	[82]
Clostridium	*beijerinckii*	RZF-1108	0.61	8.38		35	10.0	10 g/L glucose	[218]
Clostridium	*beijerinckii*	RZF-1108	1.86	4.46		35	7.0	10 g/L glucose	[218]
Clostridium	*beijerinckii*	RZF-1108	1.97	4.65	4.93	35	7.0	9 g/L glucose	[218]
Clostridium	*butyricum*	CGS2	0.45		0.67	37	7.5	xylose (static incubation)	[219]
Clostridium	*butyricum*	CGS2	0.58		4.02	37	7.5	xylose (incubation by shaking)	[219]
Clostridium	*butyricum*	CGS2	1.1		10.3	37	7.5	sucrose (incubation by shaking)	[219]
Clostridium	*butyricum*	CGS2	1.35		11.2	37	7.5	sucrose (static incubation)	[219]
Clostridium	*butyricum*	CGS2			5.53	37		cassava starch	[220]
Clostridium	*butyricum*	CGS2			5.27	37		starch	[220]
Clostridium	*butyricum*	CGS2			9.4	37		soluble starch	[221]
Clostridium	*butyricum*	CGS2			0.59	37		rice husk	[221]
Clostridium	*butyricum*	CGS2			0.46	37		*Arabidopsis* sp. starch (wildtype)	[221]
Clostridium	*butyricum*	CGS2			1.47	37		*Arabidopsis* sp. starch (*sex1-1* mutant)	[221]
Clostridium	*butyricum*	CGS5	0.7		2.79	37	7.5	xylan hydrolysate (static incubation)	[222]
Clostridium	*butyricum*	CGS5	0.76		1.2	37	7.5	pretreated rice straw hydrolysate	[222]
Clostridium	*butyricum*	CGS5			0.007	37	7.5	pretreated rice straw	[222]
Clostridium	*butyricum*	CGS5			1.58	37		xylan	[222]
Clostridium	*butyricum*	CGS5	0.5		5.35	37	7.5	10 g COD/L xylose	[219]
Clostridium	*butyricum*	CGS5	0.6		4.11	37	7.5	5 g COD/L xylose	[219]
Clostridium	*butyricum*	CGS5	0.65		10.3	37	7.5	30 g COD/L xylose	[219]

Clostridium	*butyricum*	CGS5	0.65		11.8	37	7.5	40 g COD/L xylose	[219]
Clostridium	*butyricum*	CGS5	0.7		9.59	37	7.5	20 g COD/L xylose	[219]
Clostridium	*butyricum*	CGS5	0.72		5.58	37	7.5	xylose (static incubation)	[219]
Clostridium	*butyricum*	CGS5	1.3		8.92	37	7.5	sucrose (static incubation)	[219]
Clostridium	*butyricum*	CGS5	0.98		11.87	37	7.5	sucrose (incubation by shaking)	[29]
Clostridium	*butyricum*	CGS5	1.01		8.54	37	7.5	glucose	[29]
Clostridium	*butyricum*	CGS5	1.05		2.57	37	7.5	xylose (incubation by shaking)	[29]
Clostridium	*butyricum*	CGS5	1.15		7.53	37	7.5	fructose	[29]
Clostridium	*butyricum*	CGS5			0.005	37	7.5	0.97 g/L carboxymethyl cellulose hydrolysate	[223]
Clostridium	*butyricum*	CGS5			0.044	37	7.5	1.27 g/L carboxymethyl cellulose hydrolysate	[223]
Clostridium	*butyricum*	CGS5			0.014	37	7.5	xylan hydrolysate (incubation by shaking)	[223]
Clostridium	*butyricum*	CGS5			9.2	37		soluble starchw	[221]
Clostridium	*butyricum*	DSM 10702			9.15	30	7.5	glucose	[224]
Clostridium	*butyricum*	IAM 19002			1.44	37	7	glucose	[120]
Clostridium	*butyricum*	IAM 19003			1.67	37	7	glucose	[120]
Clostridium	*butyricum*	IFO 3847			1.75	37	7	glucose	[120]
Clostridium	*butyricum*	IFO 3847	1.64		0.45	37	7.0	glucose (cells gel-immobilized)	[225]
Clostridium	*butyricum*	IFO 3847	3.55		0.975	37	7.0	glucose (cells gel-immobilized, washing and reutilization)	[225]
Clostridium	*butyricum*	IFO13949	1.8			30	7.0	starch, 15 mM glutamat acid	[226]
Clostridium	*butyricum*	IFO13949	1.9			30	7.0	starch, 5 mM glutamat acid	[226]
Clostridium	*butyricum*	IFO13949	1.9			30	7.0	starch, 10 mM glutamat acid	[226]
Clostridium	*butyricum*	M1	0.11		0.22	40		glucose	[227]
Clostridium	*butyricum*	M1			0.16	40		brewery yeast waste	[227]
Clostridium	*butyricum*	SC-E1	2.0			30	6.7	glucose and polypeptone	[228]
Clostridium	*butyricum*	TISTR 1032	0.75	5.26		37	8.0	sugarcane bargasse hydrolysate	[229]

Clostridium	*butyricum*	TISTR 1032	1.07	7.44		37	7.0	sugarcane bargasse hydrolysate	[229]
Clostridium	*butyricum*	TISTR 1032	1.09	7.93		37	6.5	sugarcane bargasse hydrolysate	[229]
Clostridium	*butyricum*	TISTR 1032	1.21	8.98		37	6.0	sugarcane bargasse hydrolysate	[229]
Clostridium	*butyricum*	TISTR 1032	1.39	2.32		37	5.5	2.59 g COD/L sugarcane bargasse hydrolysate	[229]
Clostridium	*butyricum*	TISTR 1032	1.41	11.01		37	5.5	40.74 g COD/L sugarcane bargasse hydrolysate	[229]
Clostridium	*butyricum*	TISTR 1032	1.43	4.70		37	5.5	5.33 g COD/L sugarcane bargasse hydrolysate	[229]
Clostridium	*butyricum*	TISTR 1032	1.46	1.40		37	5.5	1.49 g COD/L sugarcane bargasse hydrolysate	[229]
Clostridium	*butyricum*	TISTR 1032	1.51	9.12		37	5.5	10.55 g COD/L sugarcane bargasse hydrolysate	[229]
Clostridium	*butyricum*	TISTR 1032	1.73	12.65		37	5.5	20 g COD/L sugarcane bargasse hydrolysate	[229]
Clostridium	*butyricum*	TISTR 1032	1.73	12.63		37	5.5	20.66 g COD/L sugarcane bargasse hydrolysate	[229]
Clostridium	*butyricum*		2.25			30	6.0	cellobiose	[230]
Clostridium	*butyricum*		1.63			37	7.0	glucose	[231]
Clostridium	*celerecrescens*	DSM5628	1.25			35	6.5	cellulose (Avicel)	[215]
Clostridium	*celerecrescens*	DSM5628	1.5			35	6.5	cellulose (MN 301)	[215]
Clostridium	*celerecrescens*	DSM5628	1.6			35	6.5	cellobiose	[215]
Clostridium	*cellobioparum*	DSM 1351	1.1			35	6.5	cellulose (Avicel)	[215]
Clostridium	*cellobioparum*	DSM 1351	1.45			35	6.5	cellulose (MN 301)	[215]
Clostridium	*cellobioparum*	DSM 1351	1.9			35	6.5	cellobiose	[215]
Clostridium	*cellobioparum*		1.57			39	6.8	0.6% (w/v) glucose	[232]
Clostridium	*cellobioparum*		2.04			39	6.8	0.4% (w/v) glucose (heasdspace gas regularily released)	[232]
Clostridium	*cellobioparum*		2.11			39	6.8	0.2% (w/v) glucose	[232]
Clostridium	*cellobioparum*		2.11			39	6.8	0.6% (w/v) glucose (heasdspace gas regularily released)	[232]
Clostridium	*cellobioparum*		2.24			39	6.8	0.4% (w/v) glucose	[232]

Clostridium	*cellobioparum*		2.54			39	6.8	0.2% (w/v) glucose (heasdspace gas regularily released)	[232]
Clostridium	*cellulolyticum*	ATCC 35319	1.6			35	6.5	cellulose (Avicel)	[215]
Clostridium	*cellulolyticum*	ATCC 35319	1.7			35	6.5	cellulose (MN 301)	[215]
Clostridium	*cellulolyticum*	ATCC 35319	1.8			35	6.5	cellobiose	[215]
Clostridium	*chartatabidum*		1.26			39		cellobiose	[233]
Clostridium	*cochlearium*	DSM 1285	0.084			37	7.5	glutamate	[207]
Clostridium	*cochlearium*	DSM 1285	1.0			37	7.5	citrate	[207]
Clostridium	*diolis*	JPCC H-3			0.11	40	6.8	peptone and yeast extract	[234]
Clostridium	*diolis*	JPCC H-3			0.54	40	6.8	formic acid	[234]
Clostridium	*diolis*	JPCC H-3			0.11	40	6.8	acetic acid	[234]
Clostridium	*diolis*	JPCC H-3			0.1	40	6.8	propionic acid	[234]
Clostridium	*diolis*	JPCC H-3			0.4	40	6.8	pyvuric acid	[234]
Clostridium	*diolis*	JPCC H-3			0.15	40	6.8	lactic acid	[234]
Clostridium	*diolis*	JPCC H-3			0.13	40	6.8	succinic acid	[234]
Clostridium	*diolis*	JPCC H-3			0.31	40	6.8	malelic acid	[234]
Clostridium	*diolis*	JPCC H-3			0.11	40	6.8	citric acid	[234]
Clostridium	*diolis*	JPCC H-3			0.62	40	6.8	glucose	[234]
Clostridium	*diolis*	JPCC H-3			0.53	40	6.8	acetic acid, lactic acid	[234]
Clostridium	*diolis*	JPCC H-3			0.19	40	6.8	lactic acid, succinic acid	[234]
Clostridium	*diolis*	JPCC H-3			0.13	40	6.8	acetic acid, succinic acid	[234]
Clostridium	*diolis*	JPCC H-3			0.51	40	6.8	acetic acid, lactic acid, succinic acid	[234]
Clostridium	*homoproprionicum*	LuHBu1	1.35			37	7.2	fructose	[130]
Clostridium	*hungatei*	ATCC 700212	1.65			30	7.2	cellulose and NH_4Cl	[131]
Clostridium	*hungatei*	ATCC 700212	1.92			30	7.2	cellulose	[131]
Clostridium	*pasteurianum*	CH1	1.9		13.4	37	7.5	sucrose (static incubation)	[219]
Clostridium	*pasteurianum*	CH1	2.1		15.6	37	7.5	sucrose (incubation by shaking)	[219]
Clostridium	*pasteurianum*	CH4	2.25		15.4	37	7.5	sucrose (static incubation)	[219]

Clostridium	*pasteurianum*	CH4	2.4		4.91	37	7.5	5 g COD/L sucrose	[219]
Clostridium	*pasteurianum*	CH4	3.1		17.0	37	7.5	10 g COD/L sucrose	[219]
Clostridium	*pasteurianum*	CH4	3.1		22.5	37	7.5	20 g COD/L sucrose	[219]
Clostridium	*pasteurianum*	CH4	3.15		22.8	37	7.5	sucrose (incubation by shaking)	[219]
Clostridium	*pasteurianum*	CH4	4		25.0	37	7.5	30 g COD/L sucrose	[219]
Clostridium	*pasteurianum*	CH4	4.14		25.4	37	7.5	40 g COD/L sucrose	[219]
Clostridium	*pasteurianum*	CH4	3.80			32	7.0	sucrose	[235]
Clostridium	*pasteurianum*	CH4			2.81	37		cassava starch	[220]
Clostridium	*pasteurianum*	CH4			2.23	37		starch	[220]
Clostridium	*pasteurianum*	CH5	1.5		13.6	37	7.5	sucrose (static incubation)	[219]
Clostridium	*pasteurianum*	CH5	2		18.1	37	7.5	sucrose (incubation by shaking)	[219]
Clostridium	*pasteurianum*	CH7	1.45		9.82	37	7.5	sucrose (static incubation)	[219]
Clostridium	*pasteurianum*	CH7	2.2		18.5	37	7.5	sucrose (incubation by shaking)	[219]
Clostridium	*peptidivorans*	DSM 12505	1			37	7	cysteine	[137]
Clostridium	*perfringens*	strain A			0.11	37	7	glucose	[120]
Clostridium	*perfringens*	strain B			0.14	37	7	glucose	[120]
Clostridium	*perfringens*	strain C			0.5	37	7	glucose	[120]
Clostridium	*phytofermentans*	ATCC 700394	1			35	6.5	cellulose (Avicel)	[215]
Clostridium	*phytofermentans*	ATCC 700394	1.4			35	6.5	cellulose (MN 301)	[215]
Clostridium	*phytofermentans*	ATCC 700394	1.55			35	6.5	cellobiose	[215]
Clostridium	*populeti*	DSM 5832	1.45			35	6.5	cellulose (Avicel)	[215]
Clostridium	*populeti*	DSM 5832	1.6			35	6.5	cellulose (MN 301)	[215]
Clostridium	*populeti*	DSM 5832	1.85			35	6.5	cellobiose	[215]
Clostridium	*saccharoperbutylacetonicum*	ATCC 13564	1.40			37	6.0	glucose (incubation with light)	[236]
Clostridium	*saccharoperbutylacetonicum*	ATCC 13564	3.10			37	6.0	glucose (incubation without light)	[236]
Clostridium	*saccharoperbutylacetonicum*	ATCC 27021	0.74		5	30	6.0	glucose	[237]
Clostridium	*saccharoperbutylacetonicum*	ATCC 27021	1.20		6.2	30	7.5	fructose	[237]

Clostridium	*saccharoperbutylacetonicum*	ATCC 27021	1.31		9.55	30	6.5	glucose	[237]
Clostridium	*saccharoperbutylacetonicum*	ATCC 27021	1.40		9.25	30	8.0	glucose	[237]
Clostridium	*saccharoperbutylacetonicum*	ATCC 27021	1.43		9.4	30	7.5	glucose	[237]
Clostridium	*saccharoperbutylacetonicum*	ATCC 27021	1.58		11.35	30	7.0	glucose	[237]
Clostridium	*saccharoperbutylacetonicum*	ATCC 27021	1.72		10	30	8.5	glucose	[237]
Clostridium	*saccharoperbutylacetonicum*	ATCC 27021	2.77		1.1	30	7.5	maltose	[237]
Clostridium	*saccharoperbutylacetonicum*	ATCC 27021	2.82		5.4	30	7.5	lactose	[237]
Clostridium	*saccharoperbutylacetonicum*	ATCC 27021	2.83		8.75	30	7.5	sucrose	[237]
Clostridium	*saccharoperbutylacetonicum*	ATCC 27021			8.67	30	5.0	diluted cheese whey	[238]
Clostridium	*saccharoperbutylacetonicum*	ATCC 27021			10.5	30	6.0	diluted cheese whey	[238]
Clostridium	*saccharoperbutylacetonicum*	ATCC 27021			6.54	30	7.0	diluted cheese whey	[238]
Clostridium	*saccharoperbutylacetonicum*	ATCC 27021			6.45	30	8.0	diluted cheese whey	[238]
Clostridium	*saccharoperbutylacetonicum*	ATCC 27021			5.32	30	9.0	diluted cheese whey	[238]
Clostridium	*saccharoperbutylacetonicum*	ATCC 27021			11.78	30	10.0	diluted cheese whey	[238]
Clostridium	sp.	CB5	1.6				4.0	glucose	[180]
Clostridium	sp.	CB5	2.24				5.0	glucose	[180]
Clostridium	sp.	CB5	2.66				6.0	glucose	[180]
Clostridium	sp.	CB5	2.77				7.0	glucose	[180]
Clostridium	sp.	DMHC-10	0.04		0.006	37	4.0	glucose	[230]
Clostridium	sp.	DMHC-10	2.38		2.96	37	7.0	glucose	[230]
Clostridium	sp.	DMHC-10	2.76		3.569	37	6.0	glucose	[230]
Clostridium	sp.	DMHC-10	3.35		3.978	37	5.0	glucose	[230]
Clostridium	sp.	Fanp2	2.4			36	6.5	glucose	[239]
Clostridium	sp.	Fanp2	2.53		12.3	36	6.47	glucose	[55]
Clostridium	sp.	R1	0.63			30	6.0	xylose	[230]
Clostridium	sp.	R1	1.66			30	6.0	galactose	[230]
Clostridium	sp.	R1	1.68			30	6.0	mannose	[230]
Clostridium	sp.	R1	2.01			30	6.0	glucose	[230]
Clostridium	sp.	R1	3.09			30	6.0	sucrose	[230]

Clostridium	sp.	R1	3.13			30	6.0	maltose	[230]
Clostridium	sp.	R1	3.24			30	6.0	lactose	[230]
Clostridium	sp.	R1	2.11			30	6.0	cellobiose, 200 mg/L sulfide	[53]
Clostridium	sp.	R1	2.98			30	6.0	cellobiose, 5 mg/L nitrite	[53]
Clostridium	sp.	R1	3.2			30	6.0	cellobiose, 50 mg/L sulfide	[53]
Clostridium	sp.	R1	3.21			30	6.0	cellobiose, 1 mg/L nitrite	[53]
Clostridium	sp.	R1	3.3			30	6.0	cellobiose, 75 mg/L sulfide	[53]
Clostridium	sp.	R1	3.47			30	6.0	cellobiose, 0.1 mg/L nitrite	[53]
Clostridium	sp.	R1	3.54			30	6.0	cellobiose,0.01 mg/L nitrite	[53]
Clostridium	sp.	R1	3.52-3.57			30	6.0	cellobiose	[53, 230]
Clostridium	sp.	strain no. 2	0.29		14	36	6.8	xylose	[240]
Clostridium	sp.	strain no. 2	0.3		15.6	36	6.8	xylose (incubation with albazyme)	[240]
Clostridium	sp.	strain no. 2	2.42		17.9	36	6.8	xylose	[240]
Clostridium	sp.	strain no. 2	2.63		21.5	36	6.8	glucose	[240]
Clostridium	sp.	strain no. 2			18.9	36	6.8	avicel hydrolysate	[240]
Clostridium	sp.	strain no. 2			18.8	36	6.8	xylan hydrolysate	[240]
Clostridium	sp.	strain no. 2			5.87	36	6.8	xylan	[240]
Clostridium	sp.	strain no. 2			15.6	36	6.8	xylan (incubation with albazyme)	[240]
Clostridium	sp.	TCW1			0.63	60	7.0	Whatman filter paper (static incubation)	[54]
Clostridium	sp.	TCW1			2.6	60	7.0	Whatman filter paper (incubation by shaking)	[54]
Clostridium	sp.	TCW1			2.6	60	7.0	avicel	[54]
Clostridium	sp.	TCW1			2.5	60	7.0	cellulose	[54]
Clostridium	sp.	TCW1			0.9	60	7.0	rice straw	[54]
Clostridium	sp.	TCW1			0.9	60	7.0	bargasse	[54]
Clostridium	sp.	TCW1			0.7	60	7.0	napiergrass	[54]
Clostridium	sp.	TCW1			0.7	60	7.0	wheat straw	[54]
Clostridium	sp.	TCW1			0.7	60	7.0	corncob	[54]

Clostridium	sp.	X53			6.02	35	6.8	xylan	[241]
Clostridium	sp.	X53			7.36	40	6.8	xylan	[241]
Clostridium	sp.	X53			0.76	45	6.8	xylan	[241]
Clostridium	sp.	X53			7.14	40	5.0	xylan	[241]
Clostridium	sp.	X53			10.71	40	6.0	xylan	[241]
Clostridium	sp.	X53			2.5	40	7.0	xylan	[241]
Clostridium	sp.	X53			7.05	40	6.8	xylan (pH not controlled)	[241]
Clostridium	sp.	X53			3.02	36	6.5	xylose	[241]
Clostridium	*sporosphaeroides*	ATCC 25781	0.3			37	7.5	crotonate	[207]
Clostridium	*sporosphaeroides*	ATCC 25781	0.33			37	7.5	glutamate	[207]
Clostridium	*sporosphaeroides*	ATCC 25781	0.74			37	7.5	citrate	[207]
Clostridium	*symbiosum*	HB 25	0.06			37	7.5	glutamate	[207]
Clostridium	*tetanomorphum*	DSM 528	0.197			37	7.5	glutamate	[207]
Clostridium	*thermoalcaliphilum*	DSM 7309			0.09	50	10.5	yeast extract, tryptone	[242]
Clostridium	*thermoalcaliphilum*	DSM 7309			0.37	50	10.5	yeast extract, tryptone, glucose	[242]
Clostridium	*thermoalcaliphilum*	DSM 7309			0.14	50	7.5	yeast extract, tryptone	[242]
Clostridium	*thermoalcaliphilum*	DSM 7309			0.1	50	7.5	yeast extract, tryptone, glucose	[242]
Clostridium	*thermobutyricum*	DSM 4928	0.36			57	8.4-8.6	pyruvate	[243]
Clostridium	*thermobutyricum*	DSM 4928	1.76			57	8.4-8.6	glucose and 0.3% yeast extract	[243]
Clostridium	*thermobutyricum*	DSM 4928	2.4			57	8.4-8.6	glucose and 2% yeast extract	[243]
Clostridium	*thermocellum*	ATCC 27405	0.99			60	6.38	delignified wood fibers	[244]
Clostridium	*thermocellum*	ATCC 27405	1.13			60	6.04	cellobiose	[244]
Clostridium	*thermocellum*	ATCC 27405	1.47			60	6.95	delignified wood fibers	[244]
Clostridium	*thermocellum*	ATCC 27405	1.63			60	7.28	cellobiose	[244]
Clostridium	*thermocellum*	ATCC 27405	1.7			60		whatman paper	[244]
Clostridium	*thermocellum*	ATCC 27405	1.73			60	6.88	cellobiose	[244]
Clostridium	*thermocellum*	ATCC 27405	1.9			60		cellulose	[244]

Clostridium	*thermocellum*	ATCC 27405	2.32			60	7.08	delignified wood fibers	[244]
Clostridium	*thermocellum*	ATCC 27405	1.28	3.99		60	7.25	5 g/L cellulose	[245]
Clostridium	*thermocellum*	ATCC 27405	1.90	6.41		60	7.25	1 g/L cellulose	[245]
Clostridium	*thermocellum*	ATCC 27405		8.4		60	7.35	0,1 g/L cellobiose	[246]
Clostridium	*thermocellum*	ATCC 27405		12.9		60	7.35	1,1 g/L cellobiose	[246]
Clostridium	*thermocellum*	ATCC 27405		14.6		60	7.35	4.5 g/L cellobiose	[246]
Clostridium	*thermocellum*	ATCC 27405	0.14		0.23	60	7.2	cellulose	[247]
Clostridium	*thermocellum*	ATCC 27405	0.21		0.09	60	7.2	barley hulls	[247]
Clostridium	*thermocellum*	ATCC 27405	0.22		0.23	60	7.2	dried distiller grains	[247]
Clostridium	*thermocellum*	ATCC 27405	0.23		0.24	60	7.2	barley hulls (contaminated)	[247]
Clostridium	*thermocellum*	JN4	0.8			60		microcrystalline cellulose	[142]
Clostridium	*thermocellum*	LQ8	0.85			60	7.8	cellulose	[248]
Clostridium	*thermocellum*	LQ8	2.25			60	7.8	cellulose (incubation with *M. thermoautotrohicum* ΔH)	[248]
Clostridium	*thermohydrosulfuricum*	39E	0.015			65		starch	[249]
Clostridium	*thermohydrosulfuricum*	39E	0.017			65		glucose	[249]
Clostridium	*thermohydrosulfuricum*	39E	0.017			65		maltose	[249]
Clostridium	*thermohydrosulfuricum*	ATCC 33223	0.45			60		glucose (initial heaspace gas 100% N_2)	[250]
Clostridium	*thermohydrosulfuricum*	ATCC 33223	0.55			60		glucose (initial heaspace gas 100% H_2)	[250]
Clostridium	*thermolacticum*	DSM 2910	1.46			60	7-7.2	lactose	[251]
Clostridium	*thermolacticum*	DSM 2911	2.00			65	7.2-7.4	lactose	[251]
Clostridium	*thermosuccinogenes*	DSM 5809	0.10			37	6.5	inulin	[252]
Clostridium	*thermosuccinogenes*	DSM 5809	0.14			47	6.5	inulin	[252]
Clostridium	*thermosuccinogenes*	DSM 5809	0.15			58	6.5	inulin	[252]
Clostridium	*thermosuccinogenes*	DSM 5809	0.25			70	6.5	inulin	[252]
Clostridium	*thermosulfurogenes*	4B	0.104			60		glucose	[249]
Clostridium	*thermosulfurogenes*	4B	0.123			60		maltose	[249]
Clostridium	*thermosulfurogenes*	4B	0.146			60		starch	[249]

Clostridium	*thermosulfurogenes*	DSM 2229	0.95		0.96	60	5.5-6.5	glucose	[253]
Clostridium	*thermosulfurogenes*	DSM 2229			1.32	60	5.5-6.5	pectin	[253]
Clostridium	*thermosulfurogenes*	DSM 2229			1	60	5.5-6.5	polygalacturonate	[253]
Clostridium	*thermosulfurogenes*	DSM 2229			0.72	60	5.5-6.5	xylose	[253]
Clostridium	*thermosulfurogenes*	DSM 2229			0.7	60	5.5-6.5	arabinose	[253]
Clostridium	*uliginosum*	DSM 12992	2.6			20	6.9	xylose	[254]
Clostridium	*uliginosum*	DSM 12992	2.7			20	6.9	mannose	[254]
Clostridium	*uliginosum*	DSM 12992	3.6			20	6.9	glucose	[254]
Clostridium	*uliginosum*	DSM 12992	5.8			20	6.9	cellobiose	[254]
Clostridium	*ultunense*	DSM 10521	0.004			37	7.0	cysteine	[255]
Clostridium	*ultunense*	DSM 10521	0.025			37	7.0	pyruvate	[255]
Clostridium	*ultunense*	DSM 10521	0.08			37	7.0	glucose	[255]
Enterobacter	*aerogenes*	A-1	0.84		3.25	37	6.8	glucose	[256]
Enterobacter	*aerogenes*	ATCC 29007		19		38	6.13	glucose	[86]
Enterobacter	*aerogenes*	AY-2	1.17		4.23	37	6.8	glucose	[256]
Enterobacter	*aerogenes*	HO-39	0.83			35	6.5	lactose	[257]
Enterobacter	*aerogenes*	HO-39	0.88			35	6.5	fructose	[257]
Enterobacter	*aerogenes*	HO-39	0.95			35	6.5	galactose	[257]
Enterobacter	*aerogenes*	HO-39	0.98			35	6.5	mannose	[257]
Enterobacter	*aerogenes*	HO-39	1.68			35	6.5	mannitol	[257]
Enterobacter	*aerogenes*	HO-39	1.89			35	6.5	surcose	[257]
Enterobacter	*aerogenes*	HO-39	2.16			35	6.5	maltose	[257]
Enterobacter	*aerogenes*	HO-39			13.39	30	7	glucose	[257]
Enterobacter	*aerogenes*	HO-39	1		16.95	35	6.5	glucose	[257]
Enterobacter	*aerogenes*	HO-39			13.83	38	6.5	glucose	[257]
Enterobacter	*aerogenes*	HO-39				35	6.5	starch	[257]
Enterobacter	*aerogenes*	HO-39				35	6.5	cellulose	[257]

Enterobacter	*aerogenes*	HO-39				35	6.5	dextrin	[257]
Enterobacter	*aerogenes*	HO-39			8.9	37	6.5	glucose (cells immobilized in carrageenan)	[258]
Enterobacter	*aerogenes*	HO-39			8.7	37	6.5	glucose (cells immobilized in calcium alginate)	[258]
Enterobacter	*aerogenes*	HO-39			11.2	37	6.5	glucose (cells immobilized in agar)	[258]
Enterobacter	*aerogenes*	HO-39			12.5	37	6.5	glucose (cells immobilized on polyester)	[258]
Enterobacter	*aerogenes*	HO-39			16.4	37	6.5	glucose (cells immobilized in chitosan beads)	[258]
Enterobacter	*aerogenes*	HO-39			15.3	37	6.5	glucose (cells immobilized on glass beads)	[258]
Enterobacter	*aerogenes*	HO-39			12.9	37	6.5	glucose (cells immobilized on cellulose foam)	[258]
Enterobacter	*aerogenes*	HO-39			7.2	37	6.5	glucose (cells immobilized in carrageenan)	[258]
Enterobacter	*aerogenes*	HO-39			4	37	6.5	glucose (cells immobilized in calcium alginate)	[258]
Enterobacter	*aerogenes*	HO-39			7.7	37	6.5	glucose (cells immobilized in agar)	[258]
Enterobacter	*aerogenes*	HO-39			6.3	37	6.5	glucose (cells immobilized on polyester)	[258]
Enterobacter	*aerogenes*	HO-39			6.1	37	6.5	glucose (cells immobilized in chitosan beads)	[258]
Enterobacter	*aerogenes*	HO-39			11.6	37	6.5	glucose (cells immobilized on glass beads)	[258]
Enterobacter	*aerogenes*	HO-39			6.0	37	6.5	glucose (cells immobilized on cellulose foam)	[258]
Enterobacter	*aerogenes*	HU-101	0.71			37	6.8	10 g/L glycerol (from biodiesel)	[259]
Enterobacter	*aerogenes*	HU-101	0.82			37	6.8	25 g/L glycerol	[259]
Enterobacter	*aerogenes*	HU-101	0.89			37	6.8	10 g/L glycerol	[259]
Enterobacter	*aerogenes*	HU-101	0.9			37	6.8	3.3 g/L glycerol (from biodiesel)	[259]
Enterobacter	*aerogenes*	HU-101	1.05			37	6.8	5 g/L glycerol	[259]
Enterobacter	*aerogenes*	HU-101	1.12			37	6.8	1.17 g/L glycerol (from biodiesel)	[259]
Enterobacter	*aerogenes*	HU-101	0.28			37	6.8	gluconate	[260]
Enterobacter	*aerogenes*	HU-101	0.34			37	6.8	galactose	[260]

Enterobacter	*aerogenes*	HU-101	0.39			37	6.8	fructose	[260]
Enterobacter	*aerogenes*	HU-101	0.9			37	6.8	sorbitol	[260]
Enterobacter	*aerogenes*	HU-101	0.95			37	6.8	mannitol	[260]
Enterobacter	*aerogenes*	HU-101	0.56		2.16	37	6.8	20 g/L glucose	[256]
Enterobacter	*aerogenes*	HU-101	0.80			37	6.8	10 g/L glucose	[261]
Enterobacter	*aerogenes*	HZ-3	0.83		3.42	37	6.8	glucose	[256]
Enterobacter	*aerogenes*	IAM 1183			1.37	37		lactose	[262]
Enterobacter	*aerogenes*	VP-1	1.03			37	7.2	glucose	[261]
Enterobacter	*aerogenes*	VP-1	1.10			37	6.4	glucose	[261]
Enterobacter	*aerogenes*	VP-1	1.21			37	6.0	glucose	[261]
Enterobacter	*aerogenes*	VP-1	1.21			37	6.8	glucose (pH controlled)	[261]
Enterobacter	*aerogenes*	VP-1	1.76			37	6.8	glucose (pH not controlled)	[261]
Enterobacter	*aerogenes*	VP-2	1.06			37	6.8	glucose	[261]
Enterobacter	*aerogenes*				9.4	38	7	glucose	[263]
Enterobacter	*agglomerans*	C-1	1.03			30	7.0	glucose	[264]
Enterobacter	*agglomerans*	C-2	1.035			30	7.0	glucose	[264]
Enterobacter	*agglomerans*	CDC 811-74	1.014			30	7.0	glucose	[264]
Enterobacter	*cloacae*	F.P 01	2.71			36	5.0	maltose	[265]
Enterobacter	sp.	18P16	0.34			37		cellulose	[266]
Enterobacter	sp.	CN1	2.0		1.81	40	7.0	xylose	[85]
Enterobacter	sp.	CN1			1.64	40	7.0	glucose	[85]
Enterobacter	sp.	CN1			1.45	40	7.0	sucrose	[85]
Escherichia	*coli*	BL21(DE3) pFEGA			0.026	30		glycerol	[267]
Escherichia	*coli*	BL21(DE3) pFEGA pISC			0.018	30		glycerol	[267]
Escherichia	*coli*	BL21(DE3)			0.26	37		glucose/formate (hydrogenase 1 of *E. coli)*	[268]
Escherichia	*coli*	BL21(DE3)			0.11	37		glucose (hydrogenase 1 of *E. coli)*	[268]
Escherichia	*coli*	BL21(DE3)			0.18	37		glucose (NiFe hydrogenase of *M . marinus)*	[269]

Escherichia	*coli*	BL21(DE3) Δ*IscR* pFEGA			0.076	30		glycerol	[267]
Escherichia	*coli*	BL21(DE3)H	0.006			37	7.0	glucose	[270]
Escherichia	*coli*	BL21(DE3)HFdY	0.009			37	7.0	glucose	[270]
Escherichia	*coli*	BL21(DE3)HFdYg	0.014			37	7.0	glucose	[270]
Escherichia	*coli*	BL21(DE3)HFdYz	0.007			37	7.0	glucose	[270]
Escherichia	*coli*	BL21(DE3)HFdYzg	0.022			37	7.0	glucose	[270]
Escherichia	*coli*	BW25113			2.64	37		glucose	[64]
Escherichia	*coli*	BW25113			1.1	37		formate	[65]
Escherichia	*coli*	BW25113 *fhlA*			0.11	37		glucose	[64]
Escherichia	*coli*	BW25113 *fhlA*/pCA24N			0.07	37		glucose	[64]
Escherichia	*coli*	BW25113 *fhlA*/pCA24N-FhlA			3	37		glucose	[64]
Escherichia	*coli*	BW25113 *hyaB*			0.35	37		formate	[65]
Escherichia	*coli*	BW25113 *hyaB hybC*			3.52	37		glucose	[64]
Escherichia	*coli*	BW25113 *hyaB hybC*			3.52	37		formate	[65]
Escherichia	*coli*	BW25113 *hyaB hybC fhlA*			0.09	37		glucose	[64]
Escherichia	*coli*	BW25113 *hyaB hybC fhlA*/pCA24N			0.07	37		glucose	[64]
Escherichia	*coli*	BW25113 *hyaB hybC fhlA*/pCA24N-FhlA			3	37		glucose	[64]
Escherichia	*coli*	BW25113 *hyaB hybC hycA*			5.28	37		formate	[65]
Escherichia	*coli*	BW25113 *hyaB hybC hycA fdnG*			3.08	37		formate	[65]
Escherichia	*coli*	BW25113 *hyaB hybC hycA fdnG fdoG*			10.98	37		formate	[65]
Escherichia	*coli*	BW25113 *hyaB hybC hycA fdoG*			11.66	37		formate	[65]
Escherichia	*coli*	BW25113 *hyaB hybC hycA fdoG focA*			10.56	37		formate	[65]

Escherichia	*coli*	BW25113 *hyaB hybC hycA fdoG*/pCA24N			14.5	37		formate	[65]
Escherichia	*coli*	BW25113 *hyaB hybC hycA fdoG*/pCA24N-FhlA			17.8	37		formate	[65]
Escherichia	*coli*	BW25113 *hyaB hybC hycA fnr*			1.1	37		formate	[65]
Escherichia	*coli*	BW25113 *hyaB hybC hycA focA*			5.28	37		formate	[65]
Escherichia	*coli*	BW25113 *hyaB hybC hycA focA focB*			2.64	37		formate	[65]
Escherichia	*coli*	BW25113 *hyaB hybC hycA focA focB narG*			7.92	37		formate	[65]
Escherichia	*coli*	BW25113 *hyaB hybC hycA focA focB narG*/pCA24N-FhlA			7.7	37		formate	[65]
Escherichia	*coli*	BW25113 *hyaB hybC hycA focA narG*			4.18	37		formate	[65]
Escherichia	*coli*	BW25113 *hyaB hybC hycA focA*/pCA24N-FhlA			12.8	37		formate	[65]
Escherichia	*coli*	BW25113 *hyaB hybC hycA focB*			5.72	37		formate	[65]
Escherichia	*coli*	BW25113 *hyaB hybC hycA focB fdnG*			0.13	37		formate	[65]
Escherichia	*coli*	BW25113 *hyaB hybC hycA focB fdnG fdoG*			0.2	37		formate	[65]
Escherichia	*coli*	BW25113 *hyaB hybC hycA focB fdoG*			0.24	37		formate	[65]
Escherichia	*coli*	BW25113 *hyaB hybC hycA focB narG*			6.82	37		formate	[65]
Escherichia	*coli*	BW25113 *hyaB hybC hycA focB narG*/pCA24N-FhlA			10.6	37		formate	[65]

Escherichia	*coli*	BW25113 *hyaB hybC hycA focB*/pCA24N-FhlA			13	37		formate	[65]
Escherichia	*coli*	BW25113 *hyaB hybC hycA narG*			4.84	37		formate	[65]
Escherichia	*coli*	BW25113 *hyaB hybC hycA narG*/pCA24N-FhlA			12.3	37		formate	[65]
Escherichia	*coli*	BW25113 *hyaB hybC hycA*/pCA24N			6.38	37		formate	[65]
Escherichia	*coli*	BW25113 *hyaB hybC hycA*/pCA24N-FhlA			12.1	37		formate	[65]
Escherichia	*coli*	BW25113 *hyaB hybC hycE*			0.07	37		formate	[66]
Escherichia	*coli*	BW25113 *hyaB hybC hycE*			0.1	37		glucose	[64]
Escherichia	*coli*	BW25113 *hyaB hybC hycE*/pBS(Kan)			0.02	37		fructose	[64]
Escherichia	*coli*	BW25113 *hyaB hybC hycE*/pBS(Kan)HycE			0.25	37		fructose	[64]
Escherichia	*coli*	BW25113 *hyaB hybC hycE*/pCA24N			0.06	37		formate	[65]
Escherichia	*coli*	BW25113 *hyaB hybC*/pCA24N-FhlA			10.56	37		formate	[65]
Escherichia	*coli*	BW25113 *hybC*			1.54	37		formate	[65]
Escherichia	*coli*	BW25113 *hycE*			0.1	37		glucose	[64]
Escherichia	*coli*	BW25113 *hycE*/pBS(Kan)			0.02	37		fructose	[64]
Escherichia	*coli*	BW25113 *hycE*/pBS(Kan)HycE			0.17	37		fructose	[64]
Escherichia	*coli*	BW25113 *hyfG*			2.53	37		glucose	[64]
Escherichia	*coli*	BW25113 Δ*frdC*			0.07	37	6.3	glycerol	[271]
Escherichia	*coli*	BW25113/pBS(Kan)			0.49	37		fructose	[64]
Escherichia	*coli*	BW25113/pCA24N			2.42	37		glucose	[64]
Escherichia	*coli*	BW25113/pCA24N			0.66	37		formate	[65]
Escherichia	*coli*	BW25113/pCA24N-			3.12	37		formate	[65]

		FhlA							
Escherichia	*coli*	DJT135	1.95			37		glucose	[272]
Escherichia	*coli*	epHycE17			0.46	37		formate	[66]
Escherichia	*coli*	epHycE21			1.01	37		formate	[66]
Escherichia	*coli*	epHycE23-2			0.51	37		formate	[66]
Escherichia	*coli*	epHycE39			0.44	37		formate	[66]
Escherichia	*coli*	epHycE67			0.9	37		formate	[66]
Escherichia	*coli*	epHycE70			0.7	37		formate	[66]
Escherichia	*coli*	epHycE95			1.1	37		formate	[66]
Escherichia	*coli*	FJT135	1.3			37		glucose	[272]
Escherichia	*coli*	HW1			0.04	37	6.3	glycerol	[271]
Escherichia	*coli*	HW2			0.92	37	6.3	glycerol	[271]
Escherichia	*coli*	IFO 12713			1.28	37	7	glucose	[120]
Escherichia	*coli*	J62-1			5.46	28		glucose	[273]
Escherichia	*coli*	J62-1			12.5	28		formate	[273]
Escherichia	*coli*	J62-1(pNG2)			4.31	28		glucose	[273]
Escherichia	*coli*	J62-1(pNG2)			11.5	28		formate	[273]
Escherichia	*coli*	JW135	1.25			37		glucose	[272]
Escherichia	*coli*	JW2701-1(pASKA2701)			0.18	37		glucose/formate	[69]
Escherichia	*coli*	JW2701-1(pVSC1157)			0.64	37		glucose/formate	[69]
Escherichia	*coli*	JW2701-1(pVSC133)			1.54	37		glucose/formate	[69]
Escherichia	*coli*	JW2701-1(pVSC14)			0.77	37		glucose/formate	[69]
Escherichia	*coli*	JW2701-1(pVSC363)			1.1	37		glucose/formate	[69]
Escherichia	*coli*	LJT135	1.6			37		glucose	[272]
Escherichia	*coli*	MC13-4	1			37		1 g/L glucose	[274]
Escherichia	*coli*	MC13-4	1.2			37		18.02 g/L glucose	[274]
Escherichia	*coli*	MC13-4	1.25			37		18.02 g/L glucose 100 mM formate	[274]

Escherichia	*coli*	MC13-4	1.4			37		18.02 g/L glucose 1.5 mM formate	[274]
Escherichia	*coli*	MC13-4	0.38			37	6.5	18.02 g/L glucose 15 mM formate	[275]
Escherichia	*coli*	MC4100	0.26			37	6.5	glucose	[275]
Escherichia	*coli*	shufHycE12T366			2	37		formate	[66]
Escherichia	*coli*	shufHycE1-9			1.5	37		formate	[66]
Escherichia	*coli*	shufHycE19T366			1.8	37		formate	[66]
Escherichia	*coli*	TG1			0.26	37			[67]
Escherichia	*coli*	TG1 (recombinant)			0.81	37			[67]
Escherichia	*coli*	Tikka			0.56	37	7.10	glucose	[276]
Escherichia	*coli*	Tikka			3.37	37	6.38-6.40	glucose	[276]
Escherichia	*coli*	Tikka			0.22	37	7.41	glucose	[276]
Escherichia	*coli*	Tikka			0.28	37	7.60	glucose	[276]
Escherichia	*coli*	Tikka			1.23	37	6.40	pyruvate	[276]
Escherichia	*coli*	UNF3501			5.29	28		glucose	[273]
Escherichia	*coli*	UNF3501			10.5	28		formate	[273]
Escherichia	*coli*	UNF3501(pNG2)			6	28		glucose	[273]
Escherichia	*coli*	UNF3501(pNG2)			11.9	28		formate	[273]
Escherichia	*coli*	W3110	0.54	0.58		37		glucose	[277]
Escherichia	*coli*	ZF1	0.63	0.87		37		glucose	[277]
Escherichia	*coli*	ZF10		0.32		37	7.6	glucose	[277]
Escherichia	*coli*	ZF11		0.45		37	7.6	glucose	[277]
Escherichia	*coli*	ZF13		2.58		37	7.6	glucose	[277]
Escherichia	*coli*	ZF14		0.05		37	7.6	glucose	[277]
Escherichia	*coli*	ZF15		0.15		37	7.6	glucose	[277]
Escherichia	*coli*	ZF16		0.16		37	7.6	glucose	[277]
Escherichia	*coli*	ZF17		0.30		37	7.6	glucose	[277]
Escherichia	*coli*	ZF2	0.70	0.71		37		glucose	[277]
Escherichia	*coli*	ZF3	0.96	0.85		37		glucose	[277]

Escherichia	*coli*	ZF4	0.73	0.66		37		glucose	[277]
Escherichia	*coli*	ZF5		0.51		37		glucose	[277]
Escherichia	*coli*	ZF6		0.27		37	7.6	glucose	[277]
Escherichia	*coli*	ZF7		1.56		37	7.6	glucose	[277]
Escherichia	*coli*	ZF8		0.28		37	7.6	glucose	[277]
Escherichia	*coli*	ZF9		0.29		37	7.6	glucose	[277]
Ethanoligenens	*harbinense*	B49		27.7	2.83	35	6.0	10 g/L glucose	[42]
Ethanoligenens	*harbinense*	B49	0.55			37	7.0	3 g/L glucose	[278]
Ethanoligenens	*harbinense*	B49	0.76			37	7.0	18 g/L glucose	[278]
Ethanoligenens	*harbinense*	B49	1.28			37	7.0	15 g/L glucose	[278]
Ethanoligenens	*harbinense*	B49	1.36			37	7.0	6 g/L glucose	[278]
Ethanoligenens	*harbinense*	B49	1.42			37	7.0	9 g/L glucose, 50 mM phostphate	[278]
Ethanoligenens	*harbinense*	B49	1.53			37	7.0	9 g/L glucose, 40 mM phosphate	[278]
Ethanoligenens	*harbinense*	B49	1.71			37	7.0	12 g/L glucose	[278]
Ethanoligenens	*harbinense*	B49	1.72			37	7.0	9 g/L glucose, 10 mM phosphate	[278]
Ethanoligenens	*harbinense*	B49	1.80			37	7.0	9 g/L glucose, 30 mM phosphate	[278]
Ethanoligenens	*harbinense*	B49	1.83			37	7.0	9 g/L glucose	[278]
Ethanoligenens	*harbinense*	B49	1.91			37	7.0	9 g/L glucose, 20 mM phosphate	[278]
Ethanoligenens	*harbinense*	B49	2.20			35	6.0	14.5 g/L glucose	[83]
Ethanoligenens	*harbinense*	YUAN-3	1.91	4.55		35	5.0	glucose	[279]
Fervidobacterium	*islandicum*	DSM 5733	1.52					glucose	[280]
Fusibacter	*paucivorans*	DSM 12116	0.72			37	7.3	glucose, 20 mM thiosulphate	[281]
Fusibacter	*paucivorans*	DSM 12116	3.04			37	7.3	glucose	[281]
Geobacter	*sulfurreducens*	PCA			0.0008	35		acetate	[166]
Haloanaerobacter	*chitinovorans*	OGC 229			1.2	37	7.0	glucose	[282]
Haloanaerobacter	*chitinovorans*	W3C1			1.23	37	7.0	glucose	[282]
Haloanaerobacterium	*kushneri*	ATCC 700103	0.52			40	6.5-7.5	glucose	[283]

Haloanaerobacterium	*kushneri*	VS-511	0.53			35	7.0	glucose	[283]
Haloanaerobacterium	*kushneri*	VS-732	0.48			35	6.5	glucose	[283]
Haloanaerobium	*saccharolyticum*	DSM 6643	0.62			37	7.4	glycerol	[284]
Haloanaerobium	*saccharolyticum*	DSM 6643	2.44			37	7	glucose	[284]
Haloanaerobium	*saccharolyticum*	DSM 7779	1.61			37	7	glycerol	[284]
Haloanaerobium	*saccharolyticum*	DSM 7779	2.17			37	7	glucose	[284]
Haloanerobium	*alcaliphilum*	DSM 8275	0.09			37	6.7-7.0	pyruvate	[285]
Haloanerobium	*alcaliphilum*	DSM 8275	0.26			37	6.7-7.0	glucose	[285]
Klebsiella	*oxytoca*	HP1	0.12	6.76	2.7	38	7.0	200 mM glucose	[286]
Klebsiella	*oxytoca*	HP1	0.22	7.65	3.06	38	7.0	150 mM glucose	[286]
Klebsiella	*oxytoca*	HP1	0.36	8.96	3.58	38	7.0	100 mM glucose	[286]
Klebsiella	*oxytoca*	HP1	1.0	9.62	3.90	38	7.0	50 mM glucose	[286]
Klebsiella	*oxytoca*	HP1	1.5	8		38	7.0	sucrose	[286]
Klebsiella	*oxytoca*	HP1		2.6		38	7.0	maltose	[286]
Klebsiella	*oxytoca*	HP1		2		38	7.0	starch	[286]
Klebsiella	*pneumoniae*		14.8			37	6.9-7.0	citrate, 36 µM Ni	[287]
Klebsiella	*pneumoniae*		9.5			37	6.9-7.0	citrate	[287]
Klebsiella	sp.	HE1	0.2		0.45	37	7.5	xylose (static incubation)	[219]
Klebsiella	sp.	HE1	0.2		0.45	37	7.5	sucrose (static incubation)	[219]
Klebsiella	sp.	HE1	0.33		0.34	37	7.5	xylose (incubation by shaking)	[219]
Klebsiella	sp.	HE1	0.5		0.67	37	7.5	sucrose (incubation by shaking)	[219]
Peptostreptococcus	*asaccharolyticus*	ATCC 14963	0.2			37	7.5	glutamate	[207]
Propionispora	*vibrioides*	DSM 13305	0.86			37	7.5	mannitol	[288]
Propionispora	*vibrioides*	DSM 13305	0.86			37	7.5	fructose	[288]
Propionispora	*vibrioides*	DSM 13305	0.87			37	7.5	xylitol	[288]
Propionispora	*vibrioides*	DSM 13305	1			37	7.5	erythritol	[288]
Pyrococcus	*furiosus*	DSM 3638			2	98	7.0-	yeast extract, tryptone	[39]

							7.2		
Pyrococcus	*furiosus*	DSM3638			3.25	85	6.5	keratin hydrolysate	[78]
Ruminococcus	*albus*	7	0.79			37	7	glucose	[289]
Ruminococcus	*albus*	D-7			0.4	37		cellulose	[290]
Ruminococcus	*albus*	D-7			0.53	37		cellulose, 40 mg palladium/charcoal in medium	[290]
Ruminococcus	*albus*	D-7			0.09	37		cellulose, 100 mg palladium/charcoal in medium	[290]
Ruminococcus	*albus*	D-7			0.42	37		cellulose	[290]
Ruminococcus	*albus*	D-7			0.28	37		cellulose, 20 mg palladium above medium	[290]
Ruminococcus	*albus*	D-7			0.009	37		cellulose, 50 mg palladium above medium	[290]
Ruminococcus	*albus*	DSM 20455	2.11			37		15 g/L glucose	[291]
Ruminococcus	*albus*	DSM 20455	2.12			37		10 g/L glucose	[291]
Ruminococcus	*albus*	DSM 20455	2.14			37		20 g/L glucose	[291]
Ruminococcus	*albus*	DSM 20455	2.52			37		3 g/L glucose	[291]
Ruminococcus	*albus*	DSM 20455	2.59			37		sorghum residues	[291]
Ruminococcus	*albus*	DSM 20455	2.61			37		sorghum extract	[291]
Ruminococcus	*albus*	DSM 20455	3.15			37		sorghum stalks	[291]
Ruminococcus	*albus*	DSM 20455	1.44			37		arabinose	[292]
Ruminococcus	*albus*	DSM 20455	2.07			37		xylose	[292]
Ruminococcus	*albus*	DSM 20455	2.10			37		arabinose	[292]
Ruminococcus	*albus*	DSM 20455	2.09-2.76			37		5 g/L glucose	[291-293]
Ruminococcus	*albus*	DSM 20455	5.59			37		cellobiose	[292]
Ruminococcus	*albus*	DSM 20455	1.72			37		paperboard	[293]
Ruminococcus	*albus*	DSM 20455	2.18			37		paper tissue	[293]
Ruminococcus	*albus*	DSM 20455	2.27			37		office paper	[293]
Ruminococcus	*albus*	DSM 20455	2.29			37		magazine paper	[293]
Ruminococcus	*albus*	DSM 20455	2.89			37		newspaper	[293]

Ruminococcus	*albus*		2.57					glucose	[183]
Ruminococcus	*flavefaciens*	C94	0.37			37		cellulose	[294]
Ruminococcus	*flavefaciens*		0.33			37		cellulose	[183]
Ruminococcus	sp.	18P13	0.422			37		cellulose	[266]
Salmonella	*enterica*	Gärtner	1		0.3			formate	[4]
Selenomonas	*ruminantium*	GA31			0.0003	37		glucose	[182]
Selenomonas	*ruminantium*	HD1			0.0003	37		glucose	[182]
Selenomonas	*ruminantium*	HD4	2.04			37	7.0	glucose	[295]
Selenomonas	*ruminantium*	HD4			0.002	37		glucose	[182]
Selenomonas	*ruminantium*	PC18			0.002	37		glucose	[182]
Spirochaeta	*aurantia*	J3	1.025			30	7.5	glucose	[184]
Spirochaeta	*aurantia*	J4L	1.043			30	7.5	glucose	[184]
Spirochaeta	*aurantia*	J4T	1.23			30	7.5	glucose	[184]
Spirochaeta	*aurantia*	M1	0.968			30	7.5	glucose	[184]
Spirochaeta	*aurantia*	Vinzent	1.104			30	7.5	glucose	[184]
Spirochaeta	*thermophila*	DSM 6192	2.949			64-66	6.95	glucose	[296]
Sporacetigenium	*mesophilum*	DSM 16796	1.4			39	8.8	glucose	[297]
Staphylothermus	*marinus*	DSM 3639			0.026	85	6.5	yeast extract, peptone	[298]
Thermicanus	*aegypicus*	DSM 12793	0.26			55-60	6.5-7.0	cellobiose	[299]
Thermicanus	*aegypicus*	DSM 12793	2.8			55-60	6.5-7.0	glucose	[299]
Thermoanaerobacter	*brockii*	DSM 1457	0.53			65	7.5	lactose	[251]
Thermoanaerobacter	*ethanolicus*	ATCC 31550	0.1			72	7.5	glucose	[300]
Thermoanaerobacter	*ethanolicus*	DSM 2246	0.27			65	5.8-8.5	lactose	[251]
Thermoanaerobacter	*ethanolicus*	DSM 2355	0.24			65	5.8-8.5	lactose	[251]
Thermoanaerobacter	*finnii*	DSM 3389	0.59			65	6.5-6.8	lactose	[251]
Thermoanaerobacter	*finnii*	DSM 3389	0.01			60	7.0	glucose, thiosulphate	[301]
Thermoanaerobacter	*finnii*	DSM 3389	0.03			60	7.0	xylose, thiosulphate	[301]

Thermoanaerobacter	*finnii*	DSM 3389	0.12			60	7.0	glucose	[301]
Thermoanaerobacter	*finnii*	DSM 3389	0.14			60	7.0	xylose	[301]
Thermoanaerobacter	*mathranii*	DSM 11426	0.9			70-75	6.8-7.8	xylose	[302]
Thermoanaerobacter	sp.	DSM 9801	0.01			60	7.0	glucose, thiosulphate	[301]
Thermoanaerobacter	sp.	DSM 9801	0.02			60	7.0	xylose, thiosulphate	[301]
Thermoanaerobacter	sp.	DSM 9801	0.09			60	7.0	glucose	[301]
Thermoanaerobacter	sp.	DSM 9801	0.10			60	7.0	xylose	[301]
Thermoanaerobacter	*tengcongensis*	JCM 11007	0.3			75	7.5	glucose	[303]
Thermoanaerobacter	*thermohydrosulfuricus*	DSM 2247	0.34			65	6.9-7.5	lactose	[251]
Thermoanaerobacter	*thermohydrosulfuricus*	DSM 567	3.67			65	6.9-7.5	lactose	[251]
Thermoanaerobacterium	*thermosaccharolyticum*	DSM 571	7.44			60	7-8.5	lactose	[251]
Thermoanaerobacterium	*thermosaccharolyticum*	PSU-2	0.73					glucose, NH_4Cl	[304]
Thermoanaerobacterium	*thermosaccharolyticum*	PSU-2	1.3					starch, NH_4Cl	[304]
Thermoanaerobacterium	*thermosaccharolyticum*	PSU-2	1.75					sucrose, NH_4Cl	[304]
Thermoanaerobacterium	*thermosaccharolyticum*	PSU-2	2.43					glucose, peptone	[304]
Thermoanaerobacterium	*thermosaccharolyticum*	PSU-2	2.8					starch, peptone	[304]
Thermoanaerobacterium	*thermosaccharolyticum*	PSU-2	4.18		12.12	60	6.5	20 g/L sucrose	[304]
Thermoanaerobacterium	*thermosaccharolyticum*	PSU-2	4.91		5.73		6.25	10 g/L sucrose	[304]
Thermoanaerobacterium	*thermosaccharolyticum*	PSU-2	5.06					10 g/L sucrose, peptone	[304]
Thermoanaerobacterium	*thermosaccharolyticum*	PSU-2	5.45			60	6.5	5 g/L sucrose	[304]
Thermoanaerobium	*brockii*	HTD4	0.04			65		pyruvate	[192]
Thermoanaerobium	*brockii*	HTD4	0.05			65		glucose, 600 µmol acetone	[192]
Thermoanaerobium	*brockii*	HTD4	0.09			65		glucose 200 µmol acetone	[192]
Thermoanaerobium	*brockii*	HTD4	0.3			65		glucose	[192]
Thermoanaerobium	*thermosaccharolyticum*	W16	2.19	8.84	10.7	60	7.0	xylose	[305]
Thermoanaerobium	*thermosaccharolyticum*	W16	2.23	9.03	11.2	60	7.0	glucose/xylose (20/80)	[305]
Thermoanaerobium	*thermosaccharolyticum*	W16	2.32	9.62	12.5	60	7.0	glucose/xylose (50/50)	[305]
Thermoanaerobium	*thermosaccharolyticum*	W16	2.37	9.7	12.7	60	7.0	glucose/xylose (80/20)	[305]

Thermoanaerobium	*thermosaccharolyticum*	W16	2.42	9.7	12.2	60	7.0	glucose	[305]
Thermoanaerobium	*thermosaccharolyticum*	W16	2.4		12.7	60	7.0	corn stover hydrolysate	[305]
Thermoanaerovibrio	*velox*	DSM 12556	0.7			60-65	7.3	glucose	[306]
Thermobrachium	*celere*	DSM 8682	1.64			67	8.2	25 mM glucose, yeast extract 50 mM phosphate	[194]
Thermobrachium	*celere*	DSM 8682	1.78			67	8.2	25 mM glucose, yeast extract	[194]
Thermobrachium	*celere*	DSM 8682	2.04			67	8.2	25 mM glucose, yeast extract 25 mM phosphate	[194]
Thermobrachium	*celere*	DSM 8682	2.49			67	8.2	50 mM glucose, 1 mM $FeSO_4$	[194]
Thermobrachium	*celere*	DSM 8682	2.60			67	8.2	50 mM glucose, 10 mM $FeSO_4$	[194]
Thermobrachium	*celere*	DSM 8682	2.67			67	8.2	25 mM glucose, yeast extract 12.5 mM phosphate	[194]
Thermobrachium	*celere*	DSM 8682	2.83			67	8.2	50 mM glucose, 25 mM $FeSO_4$	[194]
Thermobrachium	*celere*	DSM 8682	2.87			67	8.2	50 mM glucose, 50 mM $FeSO_4$	[194]
Thermobrachium	*celere*	DSM 8682	2.93			67	8.2	50 mM glucose, 100 mM $FeSO_4$	[194]
Thermobrachium	*celere*	DSM 8682	3.08			67	8.2	50 mM glucose, 200 mM $FeSO_4$	[194]
Thermococcus	*litoralis*	DSM 5473			3.25	85	6.5	keratin hydrolysate	[78]
Thermococcus	*litoralis*	DSM 5473			4.70	85	6.5	5% (w/v) peptone	[78]
Thermococcus	*litoralis*	DSM 5473			6.75	85	6.5	10% (w/v) peptone	[78]
Thermosipho	*africanus*	DSM 3509	2.33					glucose	[280]
Thermotoga	*elfii*		2.8					glucose	[280]
Thermotoga	*maritima*	DSM 3109	3.4	0.24	1.45	80		xylose	[307]
Thermotoga	*maritima*	DSM 3109	3.8	0.21	1.24	80		glucose	[307]
Thermotoga	*maritima*	DSM 3109	3.8	0.24	0.97	80		arabinose	[307]
Thermotoga	*maritima*	DSM 3109	0.3			80		12 mM glucose, elemental sulphur (headspace to liquid ratio 11.5:1)	[308]
Thermotoga	*maritima*	DSM 3109	0.5			80		12 mM glucose, elemental sulphur (headspace to liquid ratio 2:1)	[308]

Thermotoga	*maritima*	DSM 3109	2			80		15 mM glucose (headspace to liquid ratio 2:1)	[308]
Thermotoga	*maritima*	DSM 3109	4			80		12 mM glucose (headspace to liquid ratio 11.5:1)	[308]
Thermotoga	*maritima*	DSM 3109	1.67		8.2	80	6.5-7.0	7.5% (w/v) glucose	[198]
Thermotoga	*naphthophila*	DSM13996	3.96			80	7.0	glucose	[309]
Thermotoga	*neapolitana*	DSM 4359	1.84		8.7	75	6.5-7.0	7.5% (w/v) glucose	[198]
Thermotoga	*neapolitana*	DSM 4359	2.04		0.05	60	7.5	14 mM glucose	[310]
Thermotoga	*neapolitana*	DSM 4359	3.09		0.27	65	7.5	14 mM glucose	[310]
Thermotoga	*neapolitana*	DSM 4359	3.18		0.47	70	7.5	14 mM glucose	[310]
Thermotoga	*neapolitana*	DSM 4359	3.75		0.8	85	7.5	14 mM glucose	[310]
Thermotoga	*neapolitana*	DSM 4359	4.11			77	7.5	14 mM glucose	[310]
Thermotoga	*neapolitana*	DSM 4359	2.7	1.67	0.42	80		xylose	[307]
Thermotoga	*neapolitana*	DSM 4359	3.2	4.17	0.61	80		glucose	[307]
Thermotoga	*neapolitana*	DSM 4359	3.2	2.08	0.59	80		arabinose	[307]
Thermotoga	*neapolitana*	DSM 4359	3.8			80		glucose, malonic acid	[311]
Thermotoga	*neapolitana*	DSM 4359	1.41			77	7.5	xylose	[312]
Thermotoga	*neapolitana*	DSM 4359	1.82			77	7.5	glucose	[312]
Thermotoga	*neapolitana*	DSM 4359	2.20			77	7.5	xylose (nitrogen sparging)	[312]
Thermotoga	*neapolitana*	DSM 4359	3.24			77	7.5	glucose (nitrogen sparging)	[312]
Thermotoga	*neapolitana*		2.26					glucose	[280]
Thermotoga	*neapolitana*				0.29	70	8.5	starch	[313]
Thermotoga	*neapolitana*				0.19	70	8.5	glucose	[313]
Thermotoga	*petrophila*	DSM13995	3.74			80	7.0	glucose	[309]
Thermotoga	sp.	SEBR 2665	2.76					glucose	[280]
Thermotoga	sp.	SEBR 7054	3.38					glucose	[280]
Victivallis	*vadensis*	DSM 14823	1.46			37	6.5	glucose	[314]
Clostridium<br>*Bacillus*	*butyricum*<br>*thermoamylovorans*	M1<br>I			0.2	40		brewery yeast waste	[227]
Clostridium<br>*Clostridium*	sp.<br>*butyricum*	R1	2.6			30	6.0	cellobiose	[230]

Clostridium<br>*Thermoanaerobacterium*	*thermocellum*<br>*thermosaccharolyticum*	JN4<br>GD17	1.8			60		microcystalline cellulose	[142]

[+] for reference please refer to manuscript
