# Supplementary material for: A comprehensive and quantitative review of dark fermentative biohydrogen production
Source: Microb Cell Fact. 2012 Aug 27;11:115. doi: 10.1186/1475-2859-11-115 (PMC3443015; doi:10.1186/1475-2859-11-115)
Supplement: Additional file 3 — Batch dark fermentative biohydrogen production[28,32,33,45,49,56,57,62,63,65,66,72,76,78,79,81,84,90,96,110,115,315-369]. [file 1475-2859-11-115-S3.pdf]

Additional file 3: Batch dark fermentative biohydrogen production

Genus	Species	Strain	$Y_{(H2/S)}$ [mol mol^{-1}]	qH_2 [mmol g^{-1} h^{-1}]	HER [mmol L^{-1} h^{-1}]	Temperature [°C]	pH	Main substrate and discriminative condition	Reference[+]
Alcaligenes	*eutrophus*	ATCC 17699			4.6	25	7.2	formate (free cells)	[315]
Alcaligenes	*eutrophus*	ATCC 17699			5.8	25	7.2	formate (cells immobilized in carrageenan)	[315]
Alcaligenes	*eutrophus*	DSM 1347			6.38	30	6.0	gluconate	[90]
Alcaligenes	*eutrophus*	DSM 515			14.1	30	6.0	gluconate	[90]
Alcaligenes	*eutrophus*	DSM 518			22	30	6.0	fructose	[90]
Alcaligenes	*eutrophus*	DSM 518			16.3	30	6.0	gluconate	[90]
Alcaligenes	*eutrophus*	DSM 518			7.7	30	6.0	lactate	[90]
Alcaligenes	*eutrophus*	HF08			0.44	30	6.0	gluconate	[90]
Alcaligenes	*hydrogenophilus*	M201			0.66	30	6.0	gluconate	[90]
Bacillus	*cereus*	EGU3	0.45			40	7.0	glucose	[316]
Bacillus	*cereus*	EGU41	0.60			40	7.0	glucose	[316]
Bacillus	*cereus*	EGU43	0.63			40	7.0	glucose	[316]
Bacillus	*cereus*	EGU44	0.53			40	7.0	glucose	[316]
Bacillus	*cereus*	EGU46	0.38			40	7.0	glucose	[316]
Bacillus	*cereus*	EGU48	0.50			40	7.0	glucose	[316]
Bacillus	*coagulans*	IIT-BT S1			4.95	37	6.0	glucose	[317]
Bacillus	*coagulans*	IIT-BT S1	2.5			37	6.5	xylose	[317]
Bacillus	*coagulans*	IIT-BT S1	1.9			37	6.5	galactose	[317]
Bacillus	*coagulans*	IIT-BT S1	2.55			37	6.5	1.5% (w/v) glucose	[317]
Bacillus	*coagulans*	IIT-BT S1			11.6	37	6.5	2% /w/v) glucose	[317]
Bacillus	*coagulans*	IIT-BT S1			11.82	37	6.5	1% (w/v) glucose	[317]
Bacillus	*coagulans*	IIT-BT S1	2.13			37	6.5	glycerol	[317]
Bacillus	*coagulans*	IIT-BT S1	2.6			37	6.5	lactose	[317]
Bacillus	*coagulans*	IIT-BT S1	2.75			37	6.5	maltose	[317]

Bacillus	*coagulans*	IIT-BT S1	2			37	6.5	mannose	[317]
Bacillus	*coagulans*	IIT-BT S1	2.5			37	6.5	starch	[317]
Bacillus	*coagulans*	IIT-BT S1	3.2			37	6.5	sucrose	[317]
Bacillus	*licheniformis*	EGU14	0.16			40	7.0	glucose	[316]
Bacillus	*licheniformis*	EGU90	0.44			40	7.0	glucose	[316]
Bacillus	*licheniformis*	JK1	0.58			40	7.0	glucose (free cells)	[96]
Bacillus	*licheniformis*	JK1	0.54-0.71			40	7.0	glucose (cells immobilized in sodium alginite)	[96]
Bacillus	*licheniformis*	JK1	0.76			40	7.0	glucose (cells immobilized in brick dust)	[96]
Bacillus	*licheniformis*	JK1	1.12			40	7.0	glucose	[96]
Bacillus	*licheniformis*	JK1	1.38			40	7.0	glucose	[96]
Bacillus	*pumilus*	EGU49	0.45			40	7.0	glucose	[316]
Bacillus	sp.	EGU15	0.31			40	7.0	glucose	[316]
Bacillus	sp.	EGU367	0.33			40	7.0	glucose	[316]
Bacillus	sp.	EGU444	0.35			40	7.0	glucose	[316]
Bacillus	sp.	EGU445	0.42			40	7.0	glucose	[316]
Bacillus	sp.	EGU447	0.36			40	7.0	glucose	[316]
Bacillus	sp.	EGU85	0.40			40	7.0	glucose	[316]
Bacillus	sp.	EGU91	0.60			40	7.0	glucose	[316]
Bacillus	sp.	MTCC 297	0.23			32	7.0	bagasse	[318]
Bacillus	*sphaericus*	EGU385	0.33			40	7.0	glucose	[316]
Bacillus	*sphaericus*	EGU399	0.29			40	7.0	glucose	[316]
Bacillus	*sphaericus*	EGU542	0.42			40	7.0	glucose	[316]
Bacillus	*subtilis*	EGU163	0.28			40	7.0	glucose	[316]
Bacillus	*subtilis*	EGU17	0.52			40	7.0	glucose	[316]
Bacillus	*subtilis*	EGU475	0.34			40	7.0	glucose	[316]
Bacillus	*thuringiensis*	EGU378	0.26			40	7.0	glucose	[316]
Bacillus	*thuringiensis*	EGU45	0.63			40	7.0	glucose	[316]

Bacillus	*thuringiensis*	EGU47	0.48			40	7.0	glucose	[316]
Bordetella	*avium*	EGU31	0.58			40	7.0	glucose	[316]
Caldicellulosiruptor	*kristjanssonii*	DSM 12137	2.6	15	17	70	6.5	glucose/xylose	[49]
Caldicellulosiruptor	*owensensis*	DSM 13100	2.7	21	17	70	6.5	glucose/xylose	[49]
Caldicellulosiruptor	*owensensis*	DSM 13100	3.8		15	70	6.7	glucose/yeast extract	[319]
Caldicellulosiruptor	*owensensis*	DSM 13100	4.0		17	70	6.7	glucose/vitamins	[319]
Caldicellulosiruptor	*owensensis*	DSM 13100	2.7		9	70	6.7	xylose/yeast extract	[319]
Caldicellulosiruptor	*owensensis*	DSM 13100	3.5		19	70	6.7	xylose/vitamins	[319]
Caldicellulosiruptor	*saccharolyticus*	DSM 8903	3.0	32	17	70	6.5	glucose/xylose	[49]
Caldicellulosiruptor	*saccharolyticus*	DSM 8903	6.6	11.7	8.4	70	7	sucrose	[57]
Caldicellulosiruptor	*saccharolyticus*	DSM 8903	2.7	22	12	71	6.5	glucose (CO_2 sparging)	[28]
Caldicellulosiruptor	*saccharolyticus*	DSM 8903	3.0	20.4	20	71	6.5	glucose (N_2 sparging)	[28]
Caldicellulosiruptor	*saccharolyticus*	DSM 8903	2.7		23	71	6.5	sucrose (N_2 sparging)	[28]
Caldicellulosiruptor	*saccharolyticus*	DSM 8903	3.1	14	11.8	71	6.5	sucrose (CO_2 sparging)	[28]
Caldicellulosiruptor	*saccharolyticus*	DSM 8903	2.4		9.7	72	6.8	28 g/L glucose/xylose	[320]
Caldicellulosiruptor	*saccharolyticus*	DSM 8903	3.3		13	72	6.8	14 g/L glucose/xylose	[320]
Caldicellulosiruptor	*saccharolyticus*	DSM 8903	3.4		12	72	6.8	10 g/L glucose/xylose	[320]
Caldicellulosiruptor	*saccharolyticus*	DSM 8903	2.4		6.2	80	6.8	28 g/L Miscanthus biomass	[320]
Caldicellulosiruptor	*saccharolyticus*	DSM 8903	3.3		10.4	80	6.8	14 g/L Miscanthus biomass	[320]
Caldicellulosiruptor	*saccharolyticus*	DSM 8903	3.4		12.6	80	6.8	10g/L Miscanthus biomass	[320]
Caldicellulosiruptor	*saccharolyticus*	DSM 8903	2.8	10.81	9.4	72	6.8	20 g/L glucose, xylose, sucrose	[321]
Caldicellulosiruptor	*saccharolyticus*	DSM 8903	3.2	12.02	10.7	72	6.8	10 g/L glucose, xylose, sucrose	[321]
Caldicellulosiruptor	*saccharolyticus*	DSM 8903	1.3	13.42	10.2	72	6.8	20 g/L sweet shorgum bargasse hydrolysate	[321]
Caldicellulosiruptor	*saccharolyticus*	DSM 8903	2.6	16.83	10.6	72	6.8	10 g/L sweet shorgum bargasse hydrolysate	[321]
Caldicellulosiruptor	*saccharolyticus*	DSM 8903	2.8		12.9	72	6.9	30 g/L glucose	[322]

Caldicellulosiruptor	*saccharolyticus*	DSM 8903	3.4		12	72	6.9	10 g/L glucose	[322]
Caldicellulosiruptor	*saccharolyticus*	DSM 8903	1.7		10.2	72	6.9	hydrolyzed potato steam peals (30-40 g/L glucose equivalents), one enzyme for pretreatment	[322]
Caldicellulosiruptor	*saccharolyticus*	DSM 8903	1.7		9.3	72	6.9	hydrolyzed potato steam peals (30-40 g/L glucose equivalents), two enzymes for pretreatment	[322]
Caldicellulosiruptor	*saccharolyticus*	DSM 8903	2.4		16.4	72	6.9	hydrolyzed potato steam peals (12-14 g/L glucose equivalents), one enzyme for pretreatment	[322]
Caldicellulosiruptor	*saccharolyticus*	DSM 8903	3.4		13.3	72	6.9	hydrolyzed potato steam peals (12-14 g/L glucose equivalents), two enzymes for pretreatment	[322]
Caldicellulosiruptor	*saccharolyticus*	DSM 8903	1.1		3.9	72	6.9	untreated potato steam peals (516 g wet weight)	[322]
Caldicellulosiruptor	*saccharolyticus*	DSM 8903	3.5		13.1	72	6.9	untreated potato steam peals (172 g wet weight)	[322]
Caldicellulosiruptor	*saccharolyticus*		2.53		10.7	70	6.4	glucose	[79]
Caldicellulosiruptor	*saccharolyticus*		2.32		9.2	70	6.4	glucose/xylose	[79]
Caldicellulosiruptor	*saccharolyticus*		1.96-3.56		5.3-6.0	70	6.4	paper sludge hydrolysate	[79]
Caldicellulosiruptor	*saccharolyticus*		2.01		5.3	70	6.4	paper sludge hydrolysate, yeast extract	[79]
Caldicellulosiruptor	*saccharolyticus*		2.24		11.3	70	6.4	xylose	[79]
Clostridium	*acetobutylicum*	NBRC 13948			1.83	30	6.6	glucose	[81]
Clostridium	*acetobutylicum*	NCIMB 13357	2.87			30	4.0	glucose	[323]

Clostridium	*acetobutylicum*	NCIMB 13357	2.61			30	4.5	glucose	[323]
Clostridium	*acetobutylicum*	NCIMB 13357	2.61			30	5.0	glucose	[323]
Clostridium	*acetobutylicum*	NCIMB 13357	2.44			30	5.5	glucose	[323]
Clostridium	*acetobutylicum*	NCIMB 13357	3.08			30	6.0	glucose	[323]
Clostridium	*acetobutylicum*	NCIMB 13357	2.97			30	6.5	glucose	[323]
Clostridium	*acetobutylicum*	NCIMB 13357	2.85-3.14			30	7.0	5 g/L glucose	[324]
Clostridium	*acetobutylicum*	NCIMB 13357	3.03			30	7.0	5 g/L glucose, 50 mg/L NaCl	[324]
Clostridium	*acetobutylicum*	NCIMB 13357	3.01			30	7.0	5 g/L glucose,100 mg/L NaCl	[324]
Clostridium	*acetobutylicum*	NCIMB 13357	3.04			30	7.0	5 g/L glucose, 250 mg/L NaCl	[324]
Clostridium	*acetobutylicum*	NCIMB 13357	3.07			30	7.0	5 g/L glucose, 500 mg/L NaCl	[324]
Clostridium	*acetobutylicum*	NCIMB 13357	3.10			30	7.0	5 g/L glucose, 1000 mg/L NaCl	[324]
Clostridium	*acetobutylicum*	NCIMB 13357	3.08			30	7.0	5 g/L glucose, 2500 mg/L NaCl	[324]
Clostridium	*acetobutylicum*	NCIMB 13357	3.08			30	7.0	5 g/L glucose, 5000 mg/L NaCl	[324]
Clostridium	*acetobutylicum*	NCIMB 13357	2.85			30	7.0	5 g/L glucose, 50 mg/L sodium acetate	[324]
Clostridium	*acetobutylicum*	NCIMB 13357	2.80			30	7.0	55 g/L glucose, 100 mg/L sodium acetate	[324]
Clostridium	*acetobutylicum*	NCIMB 13357	2.77			30	7.0	5 g/L glucose, 200 mg/L sodium acetate	[324]
Clostridium	*acetobutylicum*	NCIMB 13357	2.70			30	7.0	5 g/L glucose, 500 mg/L sodium acetate	[324]
Clostridium	*acetobutylicum*	NCIMB 13357	2.81			30	7.0	5 g/L glucose, 1000 mg/L sodium acetate	[324]
Clostridium	*acetobutylicum*	NCIMB 13357	2.81			30	7.0	5 g/L glucose, 1500 mg/L sodium acetate	[324]
Clostridium	*acetobutylicum*	NCIMB 13357	2.81			30	7.0	5 g/L glucose, 3000 mg/L sodium acetate	[324]
Clostridium	*acetobutylicum*	NCIMB 13357	2.93			30	7.0	1 g/L glucose	[323]
Clostridium	*acetobutylicum*	NCIMB 13357	3.00			30	7.0	3 g/L glucose, 50 mg/L NaCl	[323]
Clostridium	*acetobutylicum*	NCIMB 13357	2.69			30	7.0	7 g/L glucose, 50 mg/L NaCl	[323]

Clostridium	*acetobutylicum*	NCIMB 13357	2.60			30	7.0	10 g/L glucose, 50 mg/L NaCl	[323]
Clostridium	*acetobutylicum*	NCIMB 13357	2.89			30	8.0	glucose	[323]
Clostridium	*amygdalinum*	C9	2.5			37	7.5	xylose	[110]
Clostridium	*amygdalinum*	C9	1.78			37	8-8.5	arabinose	[110]
Clostridium	*beijerinckii*	AM21B			11.9	31		10 g/L glucose (pH not controlled)	[325]
Clostridium	*beijerinckii*	AM21B			11.2	31		10 g/L starch (pH not controlled)	[325]
Clostridium	*beijerinckii*	AM21B			17.9	36	5.0	10 g/L glucose	[325]
Clostridium	*beijerinckii*	AM21B			15	36	5.0	10g/L starch	[325]
Clostridium	*beijerinckii*	AM21B			21	36	5.5	10 g/L glucose	[325]
Clostridium	*beijerinckii*	AM21B			17.9	36	5.5	10g/L starch	[325]
Clostridium	*beijerinckii*	AM21B			21	36	6.0	10 g/L glucose	[325]
Clostridium	*beijerinckii*	AM21B			16.7	36	6.0	10g/L starch	[325]
Clostridium	*beijerinckii*	AM21B			22.3	36	6.5	10 g/L glucose	[325]
Clostridium	*beijerinckii*	AM21B			18.3	36	6.5	10g/L starch	[325]
Clostridium	*beijerinckii*	AM21B			20.3	36	7.0	10 g/L glucose	[325]
Clostridium	*beijerinckii*	AM21B			17.4	36	7.0	10g/L starch	[325]
Clostridium	*beijerinckii*	AM21B			12.1	36		3 g/L arabinose	[325]
Clostridium	*beijerinckii*	AM21B			18	36		3 g/L cellobiose	[325]
Clostridium	*beijerinckii*	AM21B			13.6	36		3 g/L fructose	[325]
Clostridium	*beijerinckii*	AM21B			14.1	36		3 g/L galactose	[325]
Clostridium	*beijerinckii*	AM21B			17	36		3 g/L glucose	[325]
Clostridium	*beijerinckii*	AM21B			15.8	36		10 g/L glucose	[325]
Clostridium	*beijerinckii*	AM21B			16.3	36		3 g/L lactose	[325]
Clostridium	*beijerinckii*	AM21B			14.1	36		3 g/L starch	[325]
Clostridium	*beijerinckii*	AM21B			15.6	36		10 g/L starch	[325]
Clostridium	*beijerinckii*	AM21B			16.1	36		sucrose	[325]
Clostridium	*beijerinckii*	AM21B			13.8	36		xylose	[325]

Clostridium	*beijerinckii*	AM21B			17.9	41		glucose	[325]
Clostridium	*beijerinckii*	AM21B			17.9	41		starch	[325]
Clostridium	*butyricum*	ATCC 859			1.82	30	6.4	glucose	[81]
Clostridium	*butyricum*	ATCC 860			1.08	30	6.5	glucose	[81]
Clostridium	*butyricum*	CGS5	1.73	6.07	6.2	37	5.5	30 gCOD/L sucrose	[115]
Clostridium	*butyricum*	CGS5	1.85	11.33	7.01	37	5.5	10 gCOD/L sucrose	[115]
Clostridium	*butyricum*	CGS5	1.94	10.44	6.87	37	5.5	5 gCOD/L sucrose	[115]
Clostridium	*butyricum*	CGS5	2.78, 2.91	9.46, 9.55	7.14, 7.27	37	5.5	20 gCOD/L sucrose	[115]
Clostridium	*butyricum*	CGS5	1.46	10.84	9.33	37	6	20 gCOD/L sucrose	[115]
Clostridium	*butyricum*	CGS5	1.16	8.61	7.76	37	6.5	20 gCOD/L sucrose	[115]
Clostridium	*butyricum*	CGS5	0.61	9.33	6.51	37	7.5	20 gCOD/L sucrose	[115]
Clostridium	*butyricum*	IFO 13949	2.2-2.4			37	5.25	starch	[328]
Clostridium	*butyricum*	M3-1	0.69		6.423	39	6.5	glucose (no sparging)	[326]
Clostridium	*butyricum*	M3-1	1.05		10.134	39	6.5	glucose (N_2 sparging)	[326]
Clostridium	*butyricum*	NBRC 13949			2.03	30	6.4	glucose	[81]
Clostridium	*butyricum*	NBRC 3315			2.14	30	6.4	glucose	[81]
Clostridium	*butyricum*	NBRC 3315			0.33	30		pyvuric acid	[81]
Clostridium	*butyricum*	W5	0.51			35	6.5	glucose, NH_4NO_3	[327]
Clostridium	*butyricum*	W5	0.61			35	6.5	glucose, yeast extract	[327]
Clostridium	*butyricum*	W5	1.22			35	6.5	molasses, yeast extract	[327]
Clostridium	*butyricum*	W5	0.74			35	6.5	20 g/L molasses	[328]
Clostridium	*butyricum*	W5	1.22			35	6.5	40 g/L molasses	[328]
Clostridium	*butyricum*	W5	1.44			35	6.5	60 g/L molasses	[328]
Clostridium	*butyricum*	W5	1.47			35	6.5	80 g/L molasses	[328]
Clostridium	*butyricum*	W5	1.62			35	6.5	120 g/L molasses	[328]
Clostridium	*butyricum*	W5	1.63			35	6.5	100 g/L molasses	[328]
Clostridium	*butyricum*	W5	1.11			35	6.5	waste potato starch	[327]
Clostridium	*butyricum*	W5	1.85		17.38	39	6.5	molasses, NH_4NO_3	[328]

Clostridium	*paraputrificum*	M-21	1			45	6.5	chitin (ball milled)	[329]
Clostridium	*paraputrificum*	M-21	1.3-2.5		34.8	45	6.5	GlcNAc	[72, 329, 330]
Clostridium	*paraputrificum*	M-21	2.3			45	5.5	GlcNAc	[330]
Clostridium	*paraputrificum*	M-21	1.2			45	5.8	chitin	[330]
Clostridium	*paraputrificum*	M-21	2.4			45	5.8	GlcNAc	[330]
Clostridium	*paraputrificum*	M-21	1.4		13.33	45	6.0	cellobiose	[330]
Clostridium	*paraputrificum*	M-21	1.5		9.3	45	6.0	chitin	[330]
Clostridium	*paraputrificum*	M-21	1.1		2.16	45	6.0	corn fiber	[330]
Clostridium	*paraputrificum*	M-21	2.2			45	6.0	GlcNAc	[330]
Clostridium	*paraputrificum*	M-21	1.1		10.5	45	6.0	glucose	[330]
Clostridium	*paraputrificum*	M-21	1.2		1.1	45	6.0	lobster shell	[330]
Clostridium	*paraputrificum*	M-21	1.3		1.9	45	6.0	lobster shell (pretreated)	[330]
Clostridium	*paraputrificum*	M-21	1.0		2.7	45	6.0	shrimp shell	[330]
Clostridium	*paraputrificum*	M-21	1.2		4.4	45	6.0	shrimp shell (pretreated)	[330]
Clostridium	*paraputrificum*	M-21	1.7			45	6.3	GlcNAc	[330]
Clostridium	*paraputrificum*	M-21	1.4			45	6.5	chitin	[330]
Clostridium	*paraputrificum*	M-21 (recombinant)	2.4			45	6.5	GlcNAc	[72]
Clostridium	sp.	AK 15	1.9			60	7.0	glucose	[331]
Clostridium	sp.	AK 15	1.1			60	7.0	xylose	[331]
Clostridium	sp.	HPB 49			4.69	35	6.0	glucose (H_2 sparging)	[76]
Clostridium	sp.	HPB 49			2.11	35	6.0	glucose (CO_2 sparging)	[76]
Clostridium	sp.	HPB 49			4.7	35	6.0	glucose (N_2 sparging), 10 mg $FeSO_4$ 200 mg MgCl	[76]
Clostridium	sp.	HPB 49			5.27	35	6.0	glucose (N_2 sparging), 80 mg $FeSO_4$, 200 mg MgCl	[76]
Clostridium	sp.	HPB 49			2.09	35	6.0	glucose (N_2 sparging), 10 mg $FeSO_4$,10 mg MgCl	[76]
Clostridium	sp.	HPB 49			4.72	35	6.0	glucose (N_2 sparging),	[76]

								10 mg $FeSO_4$,100 mg MgCl	
Clostridium	sp.	R3			4.92	37	4.0-4.5	glucose	[332]
Clostridium	sp.	strain No. 2	2.2		9.2	31		arabinose	[45]
Clostridium	sp.	strain No. 2	2.5		11.1	31		glucose	[45]
Clostridium	sp.	strain No. 2	2.0		9.6	31		xylose	[45]
Clostridium	sp.	strain No. 2	2.2		15	36	5.0	arabinose	[45]
Clostridium	sp.	strain No. 2	2.2		18.8	36	5.0	glucose	[45]
Clostridium	sp.	strain No. 2	2.2		15.2	36	5.0	xylose	[45]
Clostridium	sp.	strain No. 2	2.2		22.3	36	6.0	arabinose (pH controlled)	[45]
Clostridium	sp.	strain No. 2	1.2		26.8	36	6.0	75 g/L glucose	[45]
Clostridium	sp.	strain No. 2	1.6		25.9	36	6.0	20 g/L glucose	[45]
Clostridium	sp.	strain No. 2	1.8		22.3	36	6.0	10 g/L glucose (pH not controlled)	[45]
Clostridium	sp.	strain No. 2	2.2		23.9	36	6.0	10 g/L glucose (pH controlled)	[45]
Clostridium	sp.	strain No. 2	2.7		27.2	36	6.0	45 g/L glucose	[45]
Clostridium	sp.	strain No. 2	1.4		26.8	36	6.0	75 g/L xylose	[45]
Clostridium	sp.	strain No. 2	1.6		28.1	36	6.0	45 g/L xylose	[45]
Clostridium	sp.	strain No. 2	2.0		28.6	36	6.0	20 g/L xylose	[45]
Clostridium	sp.	strain No. 2	2.1		21.7	36	6.0	10 g/L xylose (pH not controlled)	[45]
Clostridium	sp.	strain No. 2	2.2		21.7	36	6.0	10 g/L xylose (pH controlled)	[45]
Clostridium	sp.	strain No. 2	2.2		20.5	36	7.0	arabinose	[45]
Clostridium	sp.	strain No. 2	2.2		21.9	36	7.0	glucose	[45]
Clostridium	sp.	strain No. 2	2.2		21.4	36	7.0	xylose	[45]
Clostridium	sp.	strain No. 2	2.3		16.5	36		xylose	[45]
Clostridium	sp.	strain No. 2	2.4		11.4	41		arabinose	[45]
Clostridium	sp.	strain No. 2	2.8		15.9	41		glucose	[45]
Clostridium	sp.	strain No. 2	2.4		13.8	41		xylose	[45]

Clostridium	sp.	W1	0.51			35		glucose	[333]
Clostridium	sp.	W1	1.21			40	7.5	glucose	[333]
Clostridium	sp.	W4	0.56			35		glucose	[333]
Clostridium	sp.	W4	1.34			40	7.5	glucose	[333]
Clostridium	sp.	W5	0.7			35		glucose	[333]
Clostridium	sp.	W5	1.77			40	7.5	glucose	[333]
Clostridium	*tryobutyricum*	JM1	1.59			37	6.0	111.2 mM glucose	[334]
Clostridium	*tryobutyricum*	JM1	0.99			37	6.3	27.8 mM glucose	[335]
Clostridium	*tryobutyricum*	JM1	1.35-1.75			37	6.3	111.2 mM glucose	[334, 335]
Clostridium	*tryobutyricum*	JM1	1.52			37	6.3	222.4 mM glucose	[335]
Clostridium	*tryobutyricum*	JM1	0.72			37	6.3	333.6 mM glucose	[335]
Clostridium	*tryobutyricum*	JM1	1.67			37	6.7	111.2 mM glucose	[334]
Enterobacter	*aerogenes*	ATCC 15038	0.63			30	6.0	glycerol wastes (160 mM), 2 mM thionine	[336]
Enterobacter	*aerogenes*	ATCC 15038	0.66			30	6.0	glycerol wastes (161 mM), 1 mM thionine	[336]
Enterobacter	*aerogenes*	ATCC 15038	0.74			30	6.0	glycerol wastes (107 mM), 2 mM thionine	[336]
Enterobacter	*aerogenes*	ATCC 15038	0.77			30	6.0	glycerol wastes (108 mM), 1 mM thionine	[336]
Enterobacter	*aerogenes*	E. 82005	0.82		2.96	37	5.8	glucose, 25 mM formate	[337]
Enterobacter	*aerogenes*	E. 82005	0.89		2.23	37	5.8	glucose, 24 mM formate	[337]
Enterobacter	*aerogenes*	E. 82005	0.72		1.81	37	6.3	glucose, 34 mM formate	[337]
Enterobacter	*aerogenes*	E. 82005	0.84		3	37	6.3	glucose,48 mM formate	[337]
Enterobacter	*aerogenes*	E.82005	0.75	3.57	2.41	37.5	5.0	glucose	[338]
Enterobacter	*aerogenes*	E.82005	1.02	12.23	11.78	37.5	5.5	glucose	[338]
Enterobacter	*aerogenes*	E.82005	1.07	12.14	17.31	37.5	6.0	glucose	[338]
Enterobacter	*aerogenes*	E.82005	0.99	12.23	11.78	37.5	6.25	glucose	[338]
Enterobacter	*aerogenes*	E.82005	1.10	9.15	16.69	37.5	6.5	glucose	[338]
Enterobacter	*aerogenes*	E.82005	0.38	4.33	8.12	37.5	7.0	glucose	[338]

Enterobacter	*aerogenes*	E.82005	0.19	1.56	2.63	37.5	7.5	glucose	[338]
Enterobacter	*aerogenes*	E.82005	0.52			38	6.0	molasses	[339]
Enterobacter	*aerogenes*	E.82005	1.07			38	6.0	molasses (H_2 sparging)	[339]
Enterobacter	*aerogenes*	E.82005	1.09			38	6.0	molasses (argon sparging)	[339]
Enterobacter	*aerogenes*	E.82005	1.16			38	6.0	molasses/peptone	[339]
Enterobacter	*aerogenes*	E.82005	1.58			38	6.0	molasses/peptone (argon sparging)	[339]
Enterobacter	*aerogenes*	EGU16	0.68			40	7.0	glucose	[316]
Enterobacter	*aerogenes*	IAM 1183	0.81		8.76	37		arabinose	[340]
Enterobacter	*aerogenes*	IAM 1183	0.26		11.04	37		galactose	[340]
Enterobacter	*aerogenes*	IAM 1183	1.30		20.14	37		mannose	[340]
Enterobacter	*aerogenes*	IAM 1183	0.56		9.48	37		rhamnose	[340]
Enterobacter	*aerogenes*	IAM 1183	0.79		9.48	37		xylose	[340]
Enterobacter	*aerogenes*	NBRC 12010			0.08	30	6.5	glucose	[81]
Enterobacter	*aerogenes*	NBRC 13543			0.25-0.38	30	6.5	glucose	[81]
Enterobacter	*aerogenes*	NBRC 13543			0.34	30		dextrin	[81]
Enterobacter	*aerogenes*	NBRC 13543			0.77	30		fructose	[81]
Enterobacter	*aerogenes*	NBRC 13543			0.85	30		galactose	[81]
Enterobacter	*aerogenes*	NBRC 13543			0.74	30		maltose	[81]
Enterobacter	*aerogenes*	NBRC 13543			0.94	30		mannitol	[81]
Enterobacter	*aerogenes*	NBRC 13543			0.77	30		mannose	[81]
Enterobacter	*aerogenes*	NBRC 13543			0.24	30		pyvuric acid	[81]
Enterobacter	*aerogenes*	NBRC 13543			0.55	30		sucrose	[81]
Enterobacter	*aerogenes*	NCIMB 10102	0.13	1.95	2.1	39	4.5	corn starch hydrolysate	[341]
Enterobacter	*aerogenes*	NCIMB 10102	0.44	2.88	2.99	39	5	corn starch hydrolysate	[341]
Enterobacter	*aerogenes*	NCIMB 10102	0.74	4.43	9.34	39	5.5	corn starch hydrolysate	[341]
Enterobacter	*aerogenes*	NCIMB 10102	0.93	9.89	11.67	39	6	corn starch hydrolysate	[341]

Enterobacter	*aerogenes*	NCIMB 10102	0.56			39	6.0	9 g/L glucose	[342]
Enterobacter	*aerogenes*	NCIMB 10102	0.60			39	6.0	18 g/L glucose	[342]
Enterobacter	*aerogenes*	NCIMB 10102	0.62			39	6.0	27 g/L glucose	[342]
Enterobacter	*aerogenes*	NCIMB 10102	0.65			39	6.0	36 g/L glucose	[342]
Enterobacter	*aerogenes*	NCIMB 10102	0.65			39	6.0	45 g/L glucose	[342]
Enterobacter	*aerogenes*	NCIMB 10102	0.65			39	6.0	54 g/L glucose	[342]
Enterobacter	*aerogenes*	NCIMB 10102	0.66			39	6.0	72 g/L glucose	[342]
Enterobacter	*aerogenes*	NCIMB 10102	1.09	9.68	17.39	39	6.5	corn starch hydrolysate	[341]
Enterobacter	*aerogenes*	NCIMB 10102	0.95	7.3	15.3	39	7	corn starch hydrolysate	[341]
Enterobacter	*aerogenes*	NCIMB 10102	0.23	3.55	8.07	39	7.5	corn starch hydrolysate	[341]
Enterobacter	*aerogenes*	NCIMB 10102	0.20	2.46	5.02	39	8	corn starch hydrolysate	[341]
Enterobacter	*aerogenes*	NCIMB 10102	0.28	2.9	8.2	40	5.5	54 g/L corn starch hydrolysate	[343]
Enterobacter	*aerogenes*	NCIMB 10102	0.34-0.45	3.0-3.6	10.3-11.3	40	5.5	36 g/L corn starch hydrolysate	[343]
Enterobacter	*aerogenes*	NCIMB 10102	0.54-1.1	5.3-8	10.7-14.6	40	5.5	18 g/L corn starch hydrolysate	[343]
Enterobacter	*aerogenes*	NCIMB 10102	0.63	3.31	5.3	40		18 g/L corn starch hydrolysate (pH not controlled)	[343]
Enterobacter	*aerogenes*	NCIMB 10102	1.1	4.14	5.3	40		10 g/L corn starch hydrolysate (pH not controlled)	[343]
Enterobacter	*aerogenes*	NCIMB 10102	1.8	4.73	5.2	40		5 g/L corn starch hydrolysate (pH not controlled)	[343]
Enterobacter	*aerogenes*	NCIMB 10102	0.19	2.38	2	40		18 g/L glucose (pH not controlled)	[343]
Enterobacter	*aerogenes*	NCIMB 10102	0.30	1.96	2	40		10 g/L glucose (pH not controlled)	[343]
Enterobacter	*asburiae*	SNU-1	0.43		0.8	25	6.1	formate	[344]
Enterobacter	*asburiae*	SNU-1	0.99		6.29	30	6.1	formate	[344]
Enterobacter	*asburiae*	SNU-1	0.08		2.81	37	4.0	glucose	[345]

Enterobacter	*asburiae*	SNU-1	0.32		5.76	37	5.0	glucose	[345]
Enterobacter	*asburiae*	SNU-1	0.41		5.76	37	5.5	glucose	[345]
Enterobacter	*asburiae*	SNU-1	0.92		4.95	37	5.8	formate	[344]
Enterobacter	*asburiae*	SNU-1	0.31		2.84	37	6.0	glucose	[345]
Enterobacter	*asburiae*	SNU-1	0.64		2.01	37	6.1	30 mM formate	[344]
Enterobacter	*asburiae*	SNU-1	0.94		4.06	37	6.1	50 mM formate	[344]
Enterobacter	*asburiae*	SNU-1	0.88-1.06		8.79-11.7	37	6.1	100 mM formate	[344]
Enterobacter	*asburiae*	SNU-1	1.07		18.5	37	6.1	270 mM formate	[344]
Enterobacter	*asburiae*	SNU-1	1.06		21.9	37	6.1	350 mM formate	[344]
Enterobacter	*asburiae*	SNU-1	1.10		11.3	37	6.1	500 mM formate	[344]
Enterobacter	*asburiae*	SNU-1	0.97		7.99	37	6.4	formate	[344]
Enterobacter	*asburiae*	SNU-1	0.30		5.62	37	6.5	glucose	[345]
Enterobacter	*asburiae*	SNU-1	0.83		6.83	37	6.7	formate	[344]
Enterobacter	*asburiae*	SNU-1	0.73		3.66	37	7.0	formate	[344]
Enterobacter	*asburiae*	SNU-1	0.54		7.81	37	7.0	10 g/L glucose	[345]
Enterobacter	*asburiae*	SNU-1			17.76	37	7.0	25 g/L glucose	[345]
Enterobacter	*asburiae*	SNU-1	0.26		2.59	37	7.1	glucose	[345]
Enterobacter	*asburiae*	SNU-1	0.06		1.07	37	7.5	glucose	[345]
Enterobacter	*asburiae*	SNU-1	0.95		10.9	42	6.1	formate	[344]
Enterobacter	*asburiae*	SNU-1	0.75		9.28	47	6.1	formate	[344]
Enterobacter	*cloacae*	A1	1.8			36	6.0	glucose	[346]
Enterobacter	*cloacae*	A2	1.8			36	6.0	glucose	[346]
Enterobacter	*cloacae*	A3	1.8			36	6.0	glucose	[346]
Enterobacter	*cloacae*	A3	0.65					glucose	[346]
Enterobacter	*cloacae*	DM 1	1.6					glucose	[346]
Enterobacter	*cloacae*	DM 10	2.1					glucose	[346]
Enterobacter	*cloacae*	DM 11	3.9		0.017	37	6	glucose	[33]
Enterobacter	*cloacae*	DM 11			0.54	37	6.5	0.2% (w/v) glucose	[347]
Enterobacter	*cloacae*	DM 11			0.57	37	6.5	0.4% (w/v) glucose	[347]

Enterobacter	*cloacae*	DM 11			0.83	37	6.5	0.6% (w/v) glucose	[347]
Enterobacter	*cloacae*	DM 11			0.85	37	6.5	0.8% (w/v) glucose	[347]
Enterobacter	*cloacae*	DM 11	3.31		1.22, 2.18	37	6.5	1.0% (w/v) glucose	[347-349]
Enterobacter	*cloacae*	DM 11			1.13	37	6.5	1.2% (w/v) glucose	[347]
Enterobacter	*cloacae*	DM 11			1.05	37	6.5	1.4% (w/v) glucose	[347]
Enterobacter	*cloacae*	DM 11			0.07	37	4.5	glucose	[348]
Enterobacter	*cloacae*	DM 11			0.78	37	5.0	glucose	[348]
Enterobacter	*cloacae*	DM 11			1.34	37	5.5	glucose	[348]
Enterobacter	*cloacae*	DM 11			1.29	37	6.0	0.2% (w/v) glucose	[348]
Enterobacter	*cloacae*	DM 11			1.16	37	6.0	0.4% (w/v) glucose	[348]
Enterobacter	*cloacae*	DM 11			1.44	37	6.0	0.6% (w/v) glucose	[348]
Enterobacter	*cloacae*	DM 11			1.63	37	6.0	0.8% (w/v) glucose	[348]
Enterobacter	*cloacae*	DM 11			2.03-2.09	37	6.0	1.0% (w/v) glucose	[348]
Enterobacter	*cloacae*	DM 11			1.83	37	6.0	1.2% (w/v) glucose	[348]
Enterobacter	*cloacae*	DM 11			1.55	37	6.0	1.4% (w/v) glucose	[348]
Enterobacter	*cloacae*	DM 11			2.03	37	7.0	glucose	[348]
Enterobacter	*cloacae*	DM 11			1.96	37	7.5	glucose	[348]
Enterobacter	*cloacae*	DM 11			1.89	37	8.0	glucose	[348]
Enterobacter	*cloacae*	DM 12	2.6					glucose	[346]
Enterobacter	*cloacae*	DM 13	1.8					glucose	[346]
Enterobacter	*cloacae*	DM 14	3.0					glucose	[346]
Enterobacter	*cloacae*	DM 15	2.1					glucose	[346]
Enterobacter	*cloacae*	DM 16	2.9					glucose	[346]
Enterobacter	*cloacae*	DM 17	2.9					glucose	[346]
Enterobacter	*cloacae*	DM 18	2.6					glucose	[346]
Enterobacter	*cloacae*	DM 19	2.9					glucose	[346]
Enterobacter	*cloacae*	DM 2	0.9					glucose	[346]
Enterobacter	*cloacae*	DM 20	3.0					glucose	[346]
Enterobacter	*cloacae*	DM 21	2.5					glucose	[346]

Enterobacter	*cloacae*	DM 22	2.6					glucose	[346]
Enterobacter	*cloacae*	DM 3	1.9					glucose	[346]
Enterobacter	*cloacae*	DM 4	0.1					glucose	[346]
Enterobacter	*cloacae*	DM 5	0.2					glucose	[346]
Enterobacter	*cloacae*	DM 6	1.7					glucose	[346]
Enterobacter	*cloacae*	DM 7	1.1					glucose	[346]
Enterobacter	*cloacae*	DM 8	0.4					glucose	[346]
Enterobacter	*cloacae*	DM 9	0.2					glucose	[346]
Enterobacter	*cloacae*	IIT-BT 08	2.2, 2.3			36	6.0	glucose	[346, 350]
Enterobacter	*cloacae*	IIT-BT 08	1.61		10.41	37	5.5	glucose (pH not controlled)	[32]
Enterobacter	*cloacae*	IIT-BT 08			15.92	37	5.5	glucose (pH controlled)	[32]
Enterobacter	*cloacae*	IIT-BT 08	1.82		17.11	37	6.0	glucose (pH not controlled)	[32]
Enterobacter	*cloacae*	IIT-BT 08			23.82	37	6.0	glucose (pH controlled)	[32]
Enterobacter	*cloacae*	IIT-BT 08	2.2		13.33	37	6.5	glucose (pH not controlled)	[32]
Enterobacter	*cloacae*	IIT-BT 08			17.8, 32.17	37	6.5	glucose (pH controlled)	[32]
Enterobacter	*cloacae*	IIT-BT 08			15.66	37	6.5	glucose (pH controlled), 0.1 mM sodium biocarbonate	[32]
Enterobacter	*cloacae*	IIT-BT 08			21.03	37	6.5	glucose (pH controlled), 0.25 mM sodium biocarbonate	[32]
Enterobacter	*cloacae*	IIT-BT 08			11.69	37	6.5	glucose (pH controlled), 0.5 mM sodium biocarbonate)	[32]
Enterobacter	*cloacae*	IIT-BT 08			9.28	37	6.5	glucose (pH controlled), 0.75 mM sodium biocarbonate)	[32]
Enterobacter	*cloacae*	IIT-BT 08	1.42		15.05	37	7.0	glucose (pH not controlled)	[32]
Enterobacter	*cloacae*	IIT-BT 08			7.76	37	7.0	glucose (pH controlled)	[32]
Enterobacter	*cloacae*	IIT-BT 08	0.88		4.88	37	7.5	glucose (pH not controlled)	[32]
Enterobacter	*cloacae*	IIT-BT 08			7.85	37	7.5	glucose (pH controlled)	[32]

Enterobacter	*cloacae*	IIT-BT 08	1.81	10.21	7.29	36	4.5	sucrose	[350]
Enterobacter	*cloacae*	IIT-BT 08	2.50	24.22	17.45	36	5.0	sucrose	[350]
Enterobacter	*cloacae*	IIT-BT 08	4.13	25.32	23.17	36	5.5	sucrose	[350]
Enterobacter	*cloacae*	IIT-BT 08			3.93	36	6.0	cellulose	[350]
Enterobacter	*cloacae*	IIT-BT 08	5.4		29	36	6.0	cellobiose	[350]
Enterobacter	*cloacae*	IIT-BT 08	2.2		19.94	36	6.0	dextrose	[350]
Enterobacter	*cloacae*	IIT-BT 08	0.95		15.53	36	6.0	xylose	[350]
Enterobacter	*cloacae*	IIT-BT 08	1.6		19.63	36	6.0	fructose	[350]
Enterobacter	*cloacae*	IIT-BT 08	1.5		16.06	36	6.0	arabinose	[350]
Enterobacter	*cloacae*	IIT-BT 08	1.4		8.03	36	6.0	maltose	[350]
Enterobacter	*cloacae*	IIT-BT 08			8.92	36	6.0	potato starch	[350]
Enterobacter	*cloacae*	IIT-BT 08	6.0		29.45	36	6.0	sucrose (pH not controlled)	[350]
Enterobacter	*cloacae*	IIT-BT 08	6.00	29.50	35.62	36	6.0	sucrose (pH controlled)	[350]
Enterobacter	*cloacae*	IIT-BT 08	3.10	24.61	21.43	36	6.5	sucrose	[350]
Enterobacter	*cloacae*	IIT-BT 08	1.75	17.58	18.29	36	7.0	sucrose	[350]
Enterobacter	*cloacae*	IIT-BT 08	0.8	5.43	6.87	36	7.5	sucrose	[350]
Enterobacter	*cloacae*	UW-C83	0.25-0.39			30	6.1	glucose	[351]
Enterobacter	sp.	BY-29	0.99			37	7.0	arabinose	[56]
Enterobacter	sp.	BY-29	1.05			37	7.0	fructose	[56]
Enterobacter	sp.	BY-29	1.28			37	7.0	galactose	[56]
Enterobacter	sp.	BY-29	1.1			37	7.0	glucose	[56]
Enterobacter	sp.	BY-29	1.46			37	7.0	maltose	[56]
Enterobacter	sp.	BY-29	1.17			37	7.0	mannose	[56]
Enterobacter	sp.	BY-29	1.09			37	7.0	sucrose	[56]
Enterobacter	sp.	BY-29	1.35			37	7.0	xylose	[56]
Escherichia	*aurescens*	no.219	0.71			35	6.2	glucose	[352]
Escherichia	*aurescens*	no.219	0.002			35	7.8	glucose	[352]
Escherichia	*aurescens*	no.220	0.91			35	6.2	glucose	[352]

Escherichia	*aurescens*	no.220	0.003			35	7.8	glucose	[352]
Escherichia	*coli*	ATCC 27325	2.5	65, 53-73.9		37	6.5	formate	[63, 353]
Escherichia	*coli*	ATCC 27325	1.08	9.5		37	6.0	glucose	[354]
Escherichia	*coli*	ATCC 700926	0.05-0.19					glycerol	[355]
Escherichia	*coli*	Ba	0.76			35	6.2	glucose	[352]
Escherichia	*coli*	Ba	0.006			35	7.8	glucose	[352]
Escherichia	*coli*	BL-21 (recombinant)	3.12			37	6	glucose	[356]
Escherichia	*coli*	BW25113 *hyaB hybC hycA fdoG*/pCA24N-FhlA			24.9	37		formate	[65]
Escherichia	*coli*	BW25113 *hyaB hybC hycA focB*/pCA24N-FhlA			13.4	37		formate	[65]
Escherichia	*coli*	BW25113 *hyaB hybC hycA*/pCA24N-FhlA			14.1	37		formate	[65]
Escherichia	*coli*	BW25113 *hyaB hybC hycE*	0.09		0.13	37		formate	[66]
Escherichia	*coli*	BW25113 *hyaB hybC hycE*/pCA24N			0.07	37		formate	[65]
Escherichia	*coli*	BW25113 *hyaB hybC*/pCA24N-FhlA			12.5	37		formate	[65]
Escherichia	*coli*	BW25113/pCA24N			0.2	37		formate	[65]
Escherichia	*coli*	BW25113/pCA24N-FhlA			1.5	37		formate	[65]
Escherichia	*coli*	DJT135	1.68			35	6.5	glucose	[84]
Escherichia	*coli*	epHycE17	0.4		0.53	37		formate	[66]
Escherichia	*coli*	epHycE21	0.558		1.03	37		formate	[66]
Escherichia	*coli*	epHycE23-2	0.33		0.51	37		formate	[66]
Escherichia	*coli*	epHycE39	0.23		0.32	37		formate	[66]
Escherichia	*coli*	epHycE67	0.44		0.88	37		formate	[66]
Escherichia	*coli*	epHycE70	0.6		1.1	37		formate	[66]
Escherichia	*coli*	epHycE95	0.7		2.27	37		formate	[66]
Escherichia	*coli*	FTD22	0.8			30	6.8	glucose	[62]

Escherichia	*coli*	FTD67	1.024			30	6.8	glucose	[62]
Escherichia	*coli*	FTD701		0.36		30	7.3	sucrose	[357]
Escherichia	*coli*	FTD701 (recombinant)		6.16		30	7.3	sucrose	[357]
Escherichia	*coli*	FTD89	1.043			30	6.8	glucose	[62]
Escherichia	*coli*	HD701		0.13		30	7.3	sucrose	[357]
Escherichia	*coli*	HD701	0.375, 0.737		2.32	30	6.8	glucose	[62, 358]
Escherichia	*coli*	HD701 (recombinant)		5.67		30	7.3	sucrose	[357]
Escherichia	*coli*	IC007 (recombinant)	0.76			30	5.5-5.7	glucose	[359]
Escherichia	*coli*	K-12			88.9	30	6.0	gluconate	[90]
Escherichia	*coli*	MC4100	0.85			30	5.5-5.7	glucose	[359]
Escherichia	*coli*	MC4100	0.764			30	6.8	glucose	[62]
Escherichia	*coli*	MG1655	0.935			37	6.3	glycerol	[360]
Escherichia	*coli*	NCIMB 11943	0.26	1.52	1.9	37		18 g/L corn starch hydrolysate	[343]
Escherichia	*coli*	NCIMB 11943	0.36	1.75	2.1	37		10 g/L corn starch hydrolysate	[343]
Escherichia	*coli*	NCIMB 11943	0.16	0.81	2.4	37		18 g/L glucose	[343]
Escherichia	*coli*	NCIMB 11943	0.25	0.80	1.8	37		10 g/L glucose	[343]
Escherichia	*coli*	NCIMB 11943	0.40	1.25	1.5	37		5 g/L glucose	[343]
Escherichia	*coli*	NCIMB 11943	0.43	0.59	1.3	37		2 g/L glucose	[343]
Escherichia	*coli*	R2	0.75			35	6.2	glucose	[352]
Escherichia	*coli*	R2	0.003			35	7.8	glucose	[352]
Escherichia	*coli*	shufHycE1-9	0.84		3	37		formate	[66]
Escherichia	*coli*	SR13		28		37	6.5	formate	[353]
Escherichia	*coli*	SR13 (recombinant)		125		37	6.5	formate	[63]
Escherichia	*coli*	SR13 (recombinant)	0.95	9.1		37	6.0	glucose	[354]
Escherichia	*coli*	SR14		37.4		37	6.0	formate	[353]
Escherichia	*coli*	SR14 (recombinant)	1.87	13		37	6.0	glucose	[354]

Escherichia	*coli*	SR15 (recombinant)	1.82	13.4		37	6.0	glucose	[354]
Proteus	*mirabilis*	EGU21	0.58			40	7.0	glucose	[316]
Proteus	*mirabilis*	EGU30	0.57			40	7.0	glucose	[316]
Proteus	*mirabilis*	EGU32	0.52			40	7.0	glucose	[316]
Proteus	*mirabilis*	EGU34	0.31			40	7.0	glucose	[316]
Pseudomonas	sp.	EGU448	0.26			40	7.0	glucose	[316]
Pseudomonas	*stutzeri*	EGU394	0.47			40	7.0	glucose	[316]
Pseudomonas	*stutzeri*	EGU396	0.26			40	7.0	glucose	[316]
Pyrococcus	*furiosus*	DSM 3638			0.008	90	7	yeast extract/peptone, elemental sulphur	[361]
Pyrococcus	*furiosus*	DSM 3638			0.016	90	7	yeast extract/peptone	[361]
Thermoanaerobacter	*thermosaccharolyticum*	W16	2.24		4.92	60	7.0	corn stover hydrolysate	[362]
Thermoanaerobacterium	sp.	AK 17	1.2			58	6.0	glucose	[331]
Thermoanaerobacterium	sp.	AK 17	1.0			58	6.0	xylose	[331]
Thermoanaerobacterium	*thermosaccharolyticum*	KU001	1.42		0.95	60	5-6	glucose	[363]
Thermoanaerobacterium	*thermosaccharolyticum*	KU001	2.39		2.19	60	5-6	glucose	[363]
Thermococcus	*litoralis*	DSM5473			0.07	85	6.5	42 mg keratin hydrolysate	[78]
Thermococcus	*litoralis*	DSM5473			0.07	85	6.5	84 mg keratin hydrolysate	[78]
Thermococcus	*litoralis*	DSM5473			0.21	85	6.5	84 mg pepton	[78]
Thermococcus	*litoralis*	DSM5473			0.14	85	6.5	42 mg peptone	[78]
Thermohydrogenium	*kirishi*		0.53					glucose	[364]
Thermotoga	*elfii*	DSM 9442	2.8-3.3	5.1-8.9	2.7-4.5	65	7.4	glucose	[57]
Thermotoga	*maritima*	DSM 3109	0.09			80	6.5	glucose	[365]
Thermotoga	*neapolitana*	DSM 4359	2.6			77	7.7	pretreated wheat straw	[307]
Thermotoga	*neapolitana*	DSM 4359	2.0		5.4	80	6.8	28 g/L Miscanthus biomass	[320]
Thermotoga	*neapolitana*	DSM 4359	3.2		12.3	80	6.8	14 g/L Miscanthus biomass	[320]
Thermotoga	*neapolitana*	DSM 4359	2.9		13.1	80	6.8	10 g/L Miscanthus biomass	[320]

Thermotoga	*neapolitana*	DSM 4359	2.5		6.1	72	6.8	28 g/L glucose/xylose	[320]
Thermotoga	*neapolitana*	DSM 4359	3.2		11.9	72	6.8	14 g/L glucose/xylose	[320]
Thermotoga	*neapolitana*	DSM 4359	3.3		14.5	72	6.8	10 g/L glucose/xylose	[320]
Thermotoga	*neapolitana*	DSM 4359	3.8					glucose	[307]
Thermotoga	*neapolitana*	DSM 4359	2.9		12.3	75	6.9	10 g/L glucose	[322]
Thermotoga	*neapolitana*	DSM 4359	3.0		12.4	75	6.9	30 g/L glucose	[322]
Thermotoga	*neapolitana*	DSM 4359	2.6		10.6	75	6.9	hydrolyzed and potato steam peals (one enzyme pretreatment)	[322]
Thermotoga	*neapolitana*	DSM 4359	3.3		8.9	75	6.9	hydrolyzed and potato steam peals (two enzyme pretreatment)	[322]
Thermotoga	*neapolitana*	DSM 4359	3.8		12.5	75	6.9	untreated potato steam peals	[322]
		B2			0.16	37		carboxymethyl cellulose	[366]
		B2			0.18	37		microcrystalline cellulose	[366]
		C3			0.61	37		carboxymethyl cellulose	[366]
		C3			0.66	37		microcrystalline cellulose	[366]
		EGU409	0.39			40	7.0	glucose	[316]
		X9			1.15	37		carboxymethyl cellulose	[366]
		X9			1.65	37		microcrystalline cellulose	[366]
Clostridium<br>*Enterobacter*	*butyricum*<br>*aerogenes*	IFO 13949<br>HO-39	1.7- 2.4			37	5.25	0.5% (w/v) starch	[367]
Clostridium<br>*Enterobacter*	*butyricum*<br>*aerogenes*	IFO 13949<br>HO-39	1.5-2.4			37	5.25	0.75% (w/v) starch	[367]
Clostridium<br>*Enterobacter*	*butyricum*<br>*aerogenes*	IFO 13949<br>HO-39	2.3			37	5.25	1% (w/v) starch	[367]
Clostridium<br>*Enterobacter*	*butyricum*<br>*aerogenes*	IFO 13949<br>HO-39	2.3			37	5.25	1.5% (w/v) starch	[367]
Clostridium<br>*Enterobacter*	*butyricum*<br>*aerogenes*	IFO 13949<br>HO-39	2.4			37	5.25	2% (w/v) starch	[367]
Clostridium<br>*Enterobacter*	*butyricum*<br>*aerogenes*	IFO 13949<br>HO-39	2.7			37	5.25	1% (w/v) starch, corn steep liquor	[368]

Ethanoligenes	*harbinense*	B49<br>B2			2.8	37	7.0	microcrystalline cellulose	[366]
Ethanoligenes	*harbinense*	B49<br>C3			2.19	37	7.0	microcrystalline cellulose	[366]
		B2<br>C3			2.57	37	7.0	microcrystalline cellulose	[366]
Ethanoligenes	*harbinense*	B49<br>X9			5.38	37	7.0	microcrystalline cellulose	[366]
		X2<br>B2			5	37	7.0	microcrystalline cellulose	[366]
		X9<br>C3			3.84	37	7.0	microcrystalline cellulose	[366]
Ethanoligenes	*harbinense*	B49<br>B2			2.35	37	7.0	carboxymethyl cellulose	[366]
Ethanoligenes	*harbinense*	B49<br>C3			2.09	37	7.0	carboxymethyl cellulose	[366]
		B2<br>C3			2.77	37	7.0	carboxymethyl cellulose	[366]
Ethanoligenes	*harbinense*	B49<br>X9			5.21	37	7.0	carboxymethyl cellulose	[366]
		X2<br>B2			4.31	37	7.0	carboxymethyl cellulose	[366]
		X9<br>C3			4.31	37	7.0	carboxymethyl cellulose	[366]
Caldicellulosiruptor<br>*Caldicellulosiruptor*	*saccharolyticus*<br>*ownsensis*	DSM 8903<br>DSM 13100	3.3	38	16	70	6.5	glucose/xylose	[49]
Caldicellulosiruptor<br>*Caldicellulosiruptor*	*saccharolyticus*<br>*ownsensis*	DSM 8903<br>DSM 13100	3.8	19	17	70	6.5	glucose/xylose	[49]
Clostridium<br>*Etanoligenes*	*acetobutylicum*<br>*harbinense*	X9<br>B49		55.4		37	4.8	microcrystalline cellulose	[369]

[+] for reference please refer to manuscript
