# Supplementary material for: A comprehensive and quantitative review of dark fermentative biohydrogen production
Source: Microb Cell Fact. 2012 Aug 27;11:115. doi: 10.1186/1475-2859-11-115 (PMC3443015; doi:10.1186/1475-2859-11-115)
Supplement: Additional file 4 — Chemostat culture dark fermentative biohydrogen production[29,34,46,47,56,75,77,96,118,122,124,125,228,257-260,279,286,291,292,331,353,356,370-400]. [file 1475-2859-11-115-S4.pdf]

Additional file 4: Chemostat culture dark fermentative biohydrogen production

Genus	Species	Strain	$Y_{(H2/S)}$ [mol mol^{-1}]	qH_2 [mmol g^{-1} h^{-1}]	HER [mmol L^{-1} h^{-1}]	Temperature [°C]	pH	Dilution rate [h^{-1}]	Main substrate and discriminative condition	Reference+
Bacillus	*licheniformis*	JK1	0.58			40	7.0		glucose, free cells	[96]
Bacillus	*licheniformis*	JK1	0.67			40	7.0		glucose, immobilized cells	[96]
Caldicellulosiruptor	*kristjanssonii*	DSM 12137	3.5	17.8	4.8	70	6.7	0.06	glucose	[34]
Caldicellulosiruptor	*kristjanssonii*	DSM 12137	3.0	34.6	10.3	70	6.7	0.15	glucose	[34]
Caldicellulosiruptor	*saccharolyticus*	DSM 8903	3.5	13.2	4.9	70	6.7	0.06	glucose	[34]
Caldicellulosiruptor	*saccharolyticus*	DSM 8903	3.1	24.4	7	70	6.7	0.15	glucose	[34]
Caldicellulosiruptor	*saccharolyticus*	DSM 8903	3.3	9.3	4.2	72	6.7	0.05	4.4 g/L glucose	[46]
Caldicellulosiruptor	*saccharolyticus*	DSM 8903	4.0	13.3	4	72	6.7	0.09	1.9 g/L glucose	[46]
Caldicellulosiruptor	*saccharolyticus*	DSM 8903	3.5	14.5	7.7	72	6.7	0.10	1.9 g/L glucose	[46]
Caldicellulosiruptor	*saccharolyticus*	DSM 8903	3.6	14.5	8.9	72	6.7	0.10	4.4 g/L glucose	[46]
Caldicellulosiruptor	*saccharolyticus*	DSM 8903	2.9	16.4	9.5	72	6.7	0.15	4.4 g/L glucose	[46]
Caldicellulosiruptor	*saccharolyticus*	DSM 8903	2.9	20.2	9.1	72	6.7	0.20	4.4 g/L glucose	[46]
Caldicellulosiruptor	*saccharolyticus*	DSM 8903	3.1	27.0	11	72	6.7	0.30	4.4 g/L glucose	[46]
Caldicellulosiruptor	*saccharolyticus*	DSM 8903	3.1-3.3	25.6-26.5	9.9-11.6	72	6.7	0.30	4.1 g/L glucose	[46]
Caldicellulosiruptor	*saccharolyticus*	DSM 8903	3.0	29.8	12.4	72	6.7	0.35	4.4 g/L glucose	[46]
Caldicellulosiruptor	*saccharolyticus*	DSM 8903	2.8		22	73	6.5		sucrose	[75]
Clostridium	*acetobutylicum*	ATCC 824			3.3	30	6.2		1.0 g/L glucose	[370]
Clostridium	*acetobutylicum*	ATCC 824			6.5	30	6.2		3.3 g/L glucose	[370]
Clostridium	*acetobutylicum*	ATCC 824			8.1	30	6.2		4.5 g/L glucose	[370]
Clostridium	*acetobutylicum*	ATCC 824			8.9	30	6.2		10.5 g/L glucose	[370]
Clostridium	*acetobutylicum*	ATCC 824		19	19	35	6.5	0.05	glucose	[371]
Clostridium	*acetobutylicum*	ATCC 824		8	8	35	6.5	0.05	glycerol/glucose (molar ratio 1:1)	[371]
Clostridium	*acetobutylicum*	ATCC 824		6	6	35	6.5	0.05	glycerol/glucose (molar ratio 2:1)	[371]
Clostridium	*acetobutylicum*	ATCC 824		6	6	35	6.5	0.05	glycerol/glucose (molar ratio 3.7:1)	[371]

Clostridium	*acetobutylicum*	ATCC 824	1.9		14.5	37	6		glucose	[372]
Clostridium	*butyricum*	CGS5	2.73		11.55	37	6.5	0.083	Sucrose, Endo medium	[29]
Clostridium	*butyricum*	CGS5	3.33		13.05	37	6.5	0.083	Sucrose, Endo medium 1	[29]
Clostridium	*butyricum*	CGS5	6.56		29.78	37	6.5	0.083	Sucrose, Endo medium 2	[29]
Clostridium	*butyricum*	DSM 5431		36.5		33	7.0		glycerol	[118]
Clostridium	*butyricum*	IFO 13949	2.3		31.9	36	4.7	0.5	glucose	[373]
Clostridium	*butyricum*	IFO 13949	1.9		51.31	36	4.7	1	glucose	[373]
Clostridium	*butyricum*	IFO 13949	1.9		20.52	36	5.5	0.2	starch	[122]
Clostridium	*butyricum*	IFO 13949			18.29	36	6.5	0.33	glucose, free cells	[373]
Clostridium	*butyricum*	IFO 13949			24.4	36	6.5	0.33	glucose, cells immobilized on poruous glass beads	[373]
Clostridium	*butyricum*	IFO 13949			16.64	36	6.5	0.33	glucose, cells immobilized in agar gel	[373]
Clostridium	*butyricum*	IFO 13949			13.83	36	6.5	0.2	glucose, free cells	[373]
Clostridium	*butyricum*	IFO 13949			15	36	6.5	0.2	glucose, cells immobilized on poruous glass beads	[373]
Clostridium	*butyricum*	IFO 13949			10.13	36	6.5	0.2	glucose, cells immobilized in agar gel	[373]
Clostridium	*butyricum*	LMG 1212	0.05				6.0	0.084	glycerol, 10 mM acetate	[124]
Clostridium	*butyricum*	LMG 1212	0.15				6.0	0.084	glycerol, 20 mM acetate	[124]
Clostridium	*butyricum*	LMG 1212	0.27				6.0	0.084	glycerol, 40 mM acetate	[124]
Clostridium	*butyricum*	LMG 1212	0.38				6.0	0.084	glycerol, 80 mM acetate	[124]
Clostridium	*butyricum*	LMG 1213	1.78				5.0	0.061	glucose, phosphate limitation (0.1 mM KH_2PO_4)	[374]
Clostridium	*butyricum*	LMG 1213	0.004				5.0	0.081	glucose, ammonium limitation	[374]
Clostridium	*butyricum*	LMG 1213	2.02				6.0	0.061	glucose, phosphate limitation (0.1 mM KH_2PO_4)	[374]
Clostridium	*butyricum*	LMG 1213	1.79				6.0	0.081	glucose, ammonium limitation	[374]
Clostridium	*butyricum*	LMG 1213	1.79				7.0	0.061	glucose, phosphate limitation (0.1 mM KH_2PO_4)	[374]

Clostridium	*butyricum*	LMG 1213	2.22				7.0	0.061	glucose, ammonium limitation	[374]
Clostridium	*butyricum*	LMG 1213	1.56				7.0	0.081	glucose, phosphate limitation (0.5 mM KH_2PO_4)	[374]
Clostridium	*butyricum*	LMG 1213	2.07				5.5-7.0	0.079	xylose	[375]
Clostridium	*butyricum*	LMG 1213	2.31				5.5-7.0	0.079	glucose	[375]
Clostridium	*butyricum*	LMG 1213	1.62				6.0	0.035	10g/L xylose	[375]
Clostridium	*butyricum*	LMG 1213	1.87				6.0	0.081	40 g/L xylose	[375]
Clostridium	*butyricum*	LMG 1213	1.81				6.0	0.087	xylose/glucose (ratio: 0.33:1)	[375]
Clostridium	*butyricum*	LMG 1213	1.81				6.0	0.087	xylose/glucose (ratio: 1:1)	[375]
Clostridium	*butyricum*	LMG 1213	1.81				6.0	0.087	xylose/glucose (ratio: 3:1)	[375]
Clostridium	*butyricum*	LMG 1213	1.99				6.0	0.087	mannitol, acetate 0.42 mmol h^{-1}	[125]
Clostridium	*butyricum*	LMG 1213	2.23				6.0	0.087	mannitol, acetate 0.78 mmol h^{-1}	[125]
Clostridium	*butyricum*	LMG 1213	2.43				6.0	0.087	mannitol, acetate 1.63 mmol h^{-1}	[125]
Clostridium	*butyricum*	LMG 1213	2.54				6.0	0.087	mannitol, acetate 2.71 mmol h^{-1}	[125]
Clostridium	*butyricum*	LMG 1213	2.04				6.0	0.087	mannitol (5.23 mmol h^{-1}), glucose (0.64 mmol h^{-1})	[125]
Clostridium	*butyricum*	LMG 1213	2.23				6.0	0.087	mannitol (3.88 mmol h^{-1}), glucose (1.96 mmol h^{-1})	[125]
Clostridium	*butyricum*	LMG 1213	2.37				6.0	0.087	mannitol (7.25 mmol h^{-1}), glucose (1.83 mmol h^{-1})	[125]
Clostridium	*butyricum*	LMG 1213	1.63				6.0	0.093	xylose	[375]
Clostridium	*butyricum*	LMG 1213	1.64				6.0	0.108	xylose	[375]
Clostridium	*butyricum*	LMG 1213	1.69				6.0	0.133	xylose	[375]
Clostridium	*butyricum*	LMG 1213	1.83				6.0	0.156	xylose	[375]
Clostridium	*butyricum*	LMG 1213	1.85				6.0	0.195	xylose	[375]
Clostridium	*butyricum*	SC-E1	2.0			30	6.7	0.125	0,5% (w/v) glucose	[228]
Clostridium	*butyricum*	SC-E1	2.2			30	6.7	0.125	0,5% (w/v) glucose, vakuum condition	[228]

Clostridium	*butyricum*	SC-E1	2.3			30	6.7	0.125	1% (w/v) glucose	[228]
Clostridium	*butyricum*	SC-E1	2.3			30	6.7	0.125	1% (w/v) glucose, vakuum condition	[228]
Clostridium	*butyricum*	VPI 3266		11.3		35	6.5	0.05	glucose	[376]
Clostridium	*butyricum*	VPI 3266		0.39		35	6.5	0.05	glucose/glycerol	[376]
Clostridium	*pasteurianum*	LMG 3285	2.16				5.0	0.081	glucose	[374]
Clostridium	*pasteurianum*	LMG 3285	1.90				5.5	0.061	glucose	[374]
Clostridium	*pasteurianum*	LMG 3285	1.82				6.0	0.061	glucose	[374]
Clostridium	*pasteurianum*	LMG 3285	2.04				6.0	0.081	glucose	[374]
Clostridium	*pasteurianum*	LMG 3285	1.41				7.0	0.061	glucose	[374]
Clostridium	*pasteurianum*	LMG 3285	2.03				7.0	0.081	glucose	[374]
Clostridium	*pasteurianum*	LMG 3285	1.50				7.5	0.061	glucose	[374]
Clostridium	*pasteurianum*	LMG 3285	2.10				7.5	0.081	glucose	[374]
Clostridium	*pasteurianum*	LMG 8285	0.87				6.0	0.096	glycerol	[124]
Clostridium	*pasteurianum*	LMG 8285	0.85				6.0	0.096	glycerol, 10 mM acetate	[124]
Clostridium	*pasteurianum*	LMG 8285	1.05				6.0	0.096	glycerol, 20 mM acetate	[124]
Clostridium	sp.	No.2	4.46		4.1	36	6.0	0.17	avicel hydrolysate	[377]
Clostridium	sp.	No.2	2.14		1.78	36	6.0	0.17	glucose	[377]
Clostridium	sp.	No.2	2.36	13.9	7.07	36	4.81	0.18	xylose	[378]
Clostridium	sp.	No.2	2.04	16.8	11.9	36	4.84	0.40	glucose	[378]
Clostridium	sp.	No.2	1.72	22.6	14.23	36	4.92	0.63	glucose	[378]
Clostridium	sp.	No.2	1.65	15.6	11.67	36	5.00	0.42	xylose	[378]
Clostridium	sp.	No.2	1.82	9.7	7.63	36	5.03	0.21	xylose	[378]
Clostridium	sp.	No.2	1.61	27.4	15.33	36	5.03	0.82	glucose	[378]
Clostridium	sp.	No.2	1.64	32.7	16	36	5.09	1.04	glucose	[378]
Clostridium	sp.	No.2	1.50	21	13.67	36	5.11	0.59	xylose	[378]
Clostridium	sp.	No.2	1.47	39	16	36	5.16	1.03	xylose	[378]
Clostridium	sp.	No.2	1.69	31.2	13.4	36	5.21	1.20	glucose	[378]
Clostridium	sp.	No.2	1.36	30.3	15.17	36	5.31	0.82	xylose	[378]

Clostridium	sp.	No.2	1.68	37.2	11.9	36	5.64	1.22	xylose	[378]
Clostridium	sp.	No.2	2.15	7.8	6.8	36	6.0	0.19	glucose	[378]
Clostridium	sp.	No.2	2.06	10.4	8.57	36	6.0	0.21	xylose	[378]
Clostridium	sp.	No.2	2.06	16.7	13.7	36	6.0	0.40	glucose	[378]
Clostridium	sp.	No.2	2.01	19.2	13.47	36	6.0	0.42	xylose	[378]
Clostridium	sp.	No.2	1.55	19	15.94	36	6.0	0.62	glucose	[378]
Clostridium	sp.	No.2	1.65	25.4	17.5	36	6.0	0.63	xylose	[378]
Clostridium	sp.	No.2	1.78	29.5	18.3	36	6.0	0.76	xylose	[378]
Clostridium	sp.	No.2	1.33	22.2	18.23	36	6.0	0.84	glucose	[378]
Clostridium	sp.	No.2	1.39	28.9	19.33	36	6.0	0.90	glucose	[378]
Clostridium	sp.	No.2	1.74	36.3	21.03	36	6.0	0.96	xylose	[378]
Clostridium	sp.	No.2	1.45	34	20.4	36	6.0	1.16	glucose	[378]
Clostridium	sp.	No.2	1.37	37.6	17.3	36	6.0	1.16	xylose	[378]
Clostridium	sp.	No.2	1.35	32.2	15.43	36	6.0	1.28	glucose	[378]
Clostridium	sp.	No.2	1.30	41.9	15.07	36	6.0	1.30	xylose	[378]
Clostridium	*stercorarium*	DSM 2910	4.4			58	6.8	0.024	lactose, co-culture with *M. thermoautotrophicus*	[77]
Clostridium	*stercorarium*	DSM 2910	4.8			58	6.8	0.024	lactose, co-culture with *M. thermoautotrophicus* and *M. thermoautotrophica*	[77]
Clostridium	*stercorarium*	DSM 2910	4.8			58	6.8	0.029	lactose, co-culture with *M. thermoautotrophicus* and *M. thermoautotrophica*	[77]
Clostridium	*stercorarium*	DSM 2910	6.4			58	6.8	0.029	lactose, co-culture with *M. thermoautotrophicus*	[77]
Clostridium	*stercorarium*	DSM 2910	4.8			58	6.8	0.040	lactose, co-culture with *M. thermoautotrophicus* and *M. thermoautotrophica*	[77]
Clostridium	*stercorarium*	DSM 2910	5.6			58	6.8	0.040	lactose, co-culture with *M. thermoautotrophicus*	[77]
Clostridium	*stercorarium*	DSM 2910	5.6			58	6.8	0.055	lactose, co-culture with *M. thermoautotrophicus*	[77]
Clostridium	*stercorarium*	DSM 2910	6			58	6.8	0.055	lactose, co-culture with *M. thermoautotrophicus* and *M. thermoautotrophica*	[77]

Clostridium	*stercorarium*	DSM 2910	5.2			58	6.8	0.067	lactose, co-culture with *M. thermoautotrophicus*	[77]
Clostridium	*stercorarium*	DSM 2910	6			58	6.8	0.067	lactose, co-culture with *M. thermoautotrophicus* and *M. thermoautotrophica*	[77]
Clostridium	*stercorarium*	DSM 2910	5.2			58	6.8	0.072	lactose, co-culture with *M. thermoautotrophicus*	[77]
Clostridium	*stercorarium*	DSM 2910	5.2			58	6.8	0.072	lactose, co-culture with *M. thermoautotrophicus* and *M. thermoautotrophica*	[77]
Clostridium	*stercorarium*	DSM 2910	4.4			58	6.8	0.100	lactose, co-culture with *M. thermoautotrophicus* and *M. thermoautotrophica*	[77]
Clostridium	*stercorarium*	DSM 2910	4.8			58	6.8	0.100	lactose, co-culture with *M. thermoautotrophicus*	[77]
Clostridium	*stercorarium*	DSM 2910	2.3	1.4	0.85	58	7.0	0.013	lactose	[379, 380]
Clostridium	*stercorarium*	DSM 2910	3	3.2	2.18	58	7.0	0.028	lactose	[380]
Clostridium	*stercorarium*	DSM 2910	2.8	3.7	2.44	58	7.0	0.040	lactose	[380]
Clostridium	*stercorarium*	DSM 2910	2.7	4.4	2.55	58	7.0	0.058	lactose	[380]
Clostridium	*stercorarium*	DSM 2910	2.4	5.1	2.46	58	7.0	0.082	lactose	[380]
Clostridium	*stercorarium*	DSM 2910	2.1	4.9	2.31	58	7.0	0.105	lactose	[380]
Clostridium	*stercorarium*	DSM 2910	2.1	5.3	2.21	58	7.0	0.130	lactose	[380]
Clostridium	*stercorarium*	DSM 2910	2.3	5.4	2.1	58	7.0	0.150	lactose	[380]
Clostridium	*stercorarium*	DSM 2910	2.5	5.6	1.52	58	7.0	0.190	lactose	[380]
Clostridium	*thermocellum*	ATCC 27405	0.98		0.29	60	7.0	0.04	1.5 g/L cellulose	[381]
Clostridium	*thermocellum*	ATCC 27405	1.29		1.01	60	7.0	0.04	4 g/L cellulose	[381]
Clostridium	*thermocellum*	ATCC 27405	1.53		0.75	60	7.0	0.04	3 g/L cellulose	[381]
Clostridium	*thermocellum*	ATCC 27405	1.65		0.58	60	7.0	0.04	2 g/L cellulose	[381]
Clostridium	*thermosaccharolyticum*	LMG 6564	1.72			55	5.4	0.10	glucose	[382]
Clostridium	*thermosaccharolyticum*	LMG 6564	1.56			55	6.0	0.09	glucose	[382]
Clostridium	*thermosaccharolyticum*	LMG 6564	1.36			55	6.0	0.10	glucose	[382]
Clostridium	*thermosaccharolyticum*	LMG 6564	1.04			55	6.8	0.08	glucose	[382]
Clostridium	*thermosaccharolyticum*	LMG 6564	1.22			55	7.0	0.03	glucose	[382]
Clostridium	*thermosaccharolyticum*	LMG 6564	1.00			55	7.0	0.05	glucose	[382]

Clostridium	*thermosaccharolyticum*	LMG 6564	1.04			55	7.0	0.08	glucose	[382]
Clostridium	*thermosaccharolyticum*	LMG 6564	0.74			55	7.0	0.09	glucose	[382]
Clostridium	*tryobutyricum*	JM1			2.05	37	6.7	0.042	glucose	[383]
Clostridium	*tryobutyricum*	JM1			2.6	37	6.7	0.056	glucose	[383]
Clostridium	*tryobutyricum*	JM1			3.81	37	6.7	0.083	glucose	[383]
Clostridium	*tryobutyricum*	JM1			4.65	37	6.7	0.11	glucose	[383]
Clostridium	*tryobutyricum*	JM1			7.44	37	6.7	0.25	glucose	[383]
Clostridium	*tryobutyricum*	JM1			11.2	37	6.7	0.33	glucose	[383]
Clostridium	*tryobutyricum*	JM1			13.4	37	6.7	0.5	glucose	[383]
Clostridium	*tryobutyricum*	JM1			3.9	37	6.7	1	glucose	[383]
Clostridium	*tyrobutyricum*	ATCC 25755	2.97			37	5.7	0.073	glucose	[384]
Clostridium	*tyrobutyricum*	ATCC 25755	2.21			37	5.7	0.083	glucose	[384]
Clostridium	*tyrobutyricum*	ATCC 25755	2.62			37	5.7	0.125	glucose	[384]
Clostridium	*tyrobutyricum*	FYa102	0.61			35	6.0	0.056	glucose, peptone	[385]
Clostridium	*tyrobutyricum*	FYa102	1.42			35	6.0	0.056	glucose, peptone, NH_4Cl	[385]
Clostridium	*tyrobutyricum*	FYa102	0.11			35	6.0	0.067	glucose, peptone	[385]
Clostridium	*tyrobutyricum*	FYa102	0.29			35	6.0	0.083	glucose, peptone, NH_4Cl	[385]
Clostridium	*tyrobutyricum*	FYa102	0.97			35	6.0	0.083	glucose, peptone	[385]
Clostridium	*tyrobutyricum*	FYa102	0.58			35	6.0	0.125	glucose, peptone, NH_4Cl	[385]
Clostridium	*tyrobutyricum*	FYa102	0.8			35	6.0	0.125	glucose, peptone	[385]
Clostridium	*tyrobutyricum*	FYa102	0.48			35	6.0	0.167	glucose, peptone, NH_4Cl	[385]
Clostridium	*tyrobutyricum*	FYa102	1			35	6.0	0.167	glucose, peptone	[385]
Clostridium	*tyrobutyricum*	FYa102	0.34		17.4	35	6.0	0.25	glucose, peptone, NH_4Cl	[385]
Clostridium	*tyrobutyricum*	FYa102	1.06			35	6.0	0.25	glucose, peptone	[385]
Clostridium	*tyrobutyricum*	FYa102	0.1			35	6.0	0.33	glucose, peptone, NH_4Cl	[385]
Clostridium	*tyrobutyricum*	FYa102	0.55			35	6.0	0.33	glucose, peptone	[385]
Clostridium	*tyrobutyricum*	FYa102	0.18			35	6.0	0.4	glucose, peptone, NH_4Cl	[385]
Clostridium	*tyrobutyricum*	FYa102	0.16			35	6.0	0.5	glucose, peptone	[385]
Clostridium	*tyrobutyricum*	FYa102	0.72			35	6.0	0.5	glucose, peptone, NH_4Cl	[385]

Enterobacter	*aerogenes*	AY-2	1.4			37	6.3	0.2	glucose	[260]
Enterobacter	*aerogenes*	AY-2	1.1		58	37	7	0.67	glucose	[386]
Enterobacter	*aerogenes*	E.82005	1.8-3.5		10-13	37.5	6.0		molasses	[387]
Enterobacter	*aerogenes*	E.82005	1.5-2.5		20-36	38	6	0.267	diluted molasses	[388]
Enterobacter	*aerogenes*	HO-39			6.24	37	5.8		glucose	[257]
Enterobacter	*aerogenes*	HO-39	0.56		20.08	37	6.5	0.067	glucose	[258]
Enterobacter	*aerogenes*	HO-39	0.81		10.71	37	6.5	0.25	glucose	[258]
Enterobacter	*aerogenes*	HO-39	0.73		37.92	37	6.5	1	glucose	[258]
Enterobacter	*aerogenes*	HU 101	0.55		30	37	7	0.67	glucose	[386]
Enterobacter	*aerogenes*	HU-101	0.8			37	6.3	0.2	glucose	[260]
Enterobacter	*aerogenes*	HU-101			30	37	6.8	0.8	glycerol (from biodiesel)	[259]
Enterobacter	*aerogenes*	HU-101			63	37	6.8	1.2	glycerol (from biodiesel)	[259]
Enterobacter	*aerogenes*	HU-101			80	37	6.8	1.3	glycerol	[259]
Enterobacter	*aerogenes*	NCIMB 10102	1.36	2.11	8.85	40	5.5		corn starch hydrolysate, feed flow rate: 32 cm^3 h^{-1}	[389]
Enterobacter	*aerogenes*	NCIMB 10102	1.54	3.36	10.15	40	5.5		corn starch hydrolysate, feed flow rate: 40 cm^3 h^{-1}	[389]
Enterobacter	*aerogenes*	NCIMB 10102	2.31	0.75	5.85	40	5.5		corn starch hydrolysate, feed flow rate: 12 cm^3 h^{-1}	[389]
Enterobacter	*aerogenes*	NCIMB 10102	2.55	1.76	9.8	40	5.5		corn starch hydrolysate, feed flow rate: 24 cm^3 h^{-1}	[389]
Enterobacter	*aerogenes*	NCIMB 10102	3.02	0.4	3.25	40	5.5		corn starch hydrolysate, feed flow rate: 4 cm^3 h^{-1}	[389]
Enterobacter	*aerogenes*	NCIMB 10102		0.33-2.83		40	5.5		corn starch hydrolysate, inlet concentration 20 mg cm^{-3}	[390]
Enterobacter	*aerogenes*	NCIMB 10102		3.2-3.9		40	5.5		corn starch hydrolysate, inlet concentration 30 mg cm^{-3}	[390]
Enterobacter	*aerogenes*	NCIMB 10102		8.3-9.8		40	5.5		corn starch hydrolysate, inlet concentration 40 mg cm^{-3}	[390]
Enterobacter	*aerogenes*	NCIMB 10102		14.5-15.8		40	5.5		corn starch hydrolysate, inlet concentration 50 mg cm^{-3}	[390]
Enterobacter	*cloacae*	IIT-BT-08			66.7	37		0.93	0.5% (w/v) glucose	[47]

Enterobacter	*cloacae*	IIT-BT-08			77.3	37		0.93	1% (w/v) glucose	[47]
Enterobacter	*cloacae*	IIT-BT-08			72.9	37		0.93	1.5% (w/v) glucose	[47]
Enterobacter	*cloacae*	IIT-BT-08			66.8	37		0.93	2% (w/v) glucose	[47]
Enterobacter	*cloacae*	IIT-BT-08			62.2	37		0.93	2.5% (w/v) glucose	[47]
Enterobacter	*cloacae*	IIT-BT-08			62.6	37		0.93	3% (w/v) glucose	[47]
Enterobacter	*cloacae*	IIT-BT-08			60.8	37		0.93	3.5% (w/v) glucose	[47]
Enterobacter	*cloacae*	IIT-BT-08			62.5	37		0.93	glucose, tubular bioreactor, packing material coir	[47]
Enterobacter	*cloacae*	IIT-BT-08			65.2	37		0.93	glucose, tampered bioreactor, packing material coir	[47]
Enterobacter	*cloacae*	IIT-BT-08			71	37		0.93	glucose, rhomboid bioreactor, packing material coir	[47]
Enterobacter	*cloacae*	IIT-BT-08			44	37		0.93	glucose, rhomboid bioreactor, packing material rice straw	[47]
Enterobacter	*cloacae*	IIT-BT-08			52	37		0.93	glucose, rhomboid bioreactor, packing material bagasse	[47]
Enterobacter	sp.	BY-29	0.73		0.2	37	7.0	0.2	glucose, cells immobilized, glass column bioreactor	[56]
Enterobacter	sp.	BY-29	0.76		0.2	37	7.0	0.2	glucose, cells immobilized, continuously stirred tank bioreactor	[56]
Enterobacter	sp.	BY-29	0.79		0.2	37	7.0	0.2	glucose, free cells, continuously stirred tank bioreactor	[56]
Enterobacter	sp.	BY-29	0.625		0.25	37	7.0	0.33	glucose, free cells, continuously stirred tank bioreactor	[56]
Enterobacter	sp.	BY-29	0.67		0.275	37	7.0	0.33	glucose, cells immobilized, continuously stirred tank bioreactor	[56]
Enterobacter	sp.	BY-29	0.71		0.3	37	7.0	0.33	glucose, cells immobilized, glass column bioreactor	[56]

Enterobacter	sp.	BY-29	0.41		0.42	37	7.0	1	glucose, free cells, continuously stirred tank bioreactor	[56]
Enterobacter	sp.	BY-29	0.5		0.585	37	7.0	1	glucose, cells immobilized, continuously stirred tank bioreactor	[56]
Enterobacter	sp.	BY-29	0.625		0.775	37	7.0	1	glucose, cells immobilized, glass column bioreactor	[56]
Escherichia	*coli*	BL-21 recombinant			54	37	6	0.25	glucose	[356]
Escherichia	*coli*	BL-21 recombinant			86.84	37	6	0.25	molasse	[356]
Escherichia	*coli*	BL-21 recombinant			52.8	37	6	0.31	glucose	[356]
Escherichia	*coli*	BL-21 recombinant			97.04	37	6	0.37	molasse	[356]
Escherichia	*coli*	BL-21 recombinant			46.6	37	6	0.50	glucose	[356]
Escherichia	*coli*	BL-21 recombinant			59.7	37	6	0.50	molasse	[356]
Escherichia	*coli*	BL-21 recombinant			66	37	6	0.55	glucose	[356]
Escherichia	*coli*	BL-21 recombinant			48.8	37	6	0.62	glucose	[356]
Escherichia	*coli*	BL-21 recombinant			51.79	37	6	0.66	molasse	[356]
Escherichia	*coli*	BL-21 recombinant			32.67	37	6	0.80	molasse	[356]
Escherichia	*coli*	BL-21 recombinant			43.3	37	6	0.81	glucose	[356]
Escherichia	*coli*	DJT135	2.36			37	6.4	0.01	glucose, sulphur limitation	[391]
Escherichia	*coli*	DJT135	2.39			37	6.4	0.01	glucose, phosphate limitation	[391]
Escherichia	*coli*	DJT135	0.45			37	6.4	0.1	glucose, sulphur limitation	[391]
Escherichia	*coli*	DJT135	2.55			37	6.4	0.1	glucose, phosphate limitation	[391]
Escherichia	*coli*	DJT135	0.26			37	6.4	0.3	glucose, sulphur limitation	[391]
Escherichia	*coli*	DJT135	0.78			37	6.4	0.3	glucose, phosphate limitation	[391]
Escherichia	*coli*	JW135	0.24			37	6.4	0.1	glucose, glucose limitation	[391]

Escherichia	*coli*	JW135	0.31			37	6.4	0.1	glucose, sulphur limitation	[391]
Escherichia	*coli*	JW135	0.36			37	6.4	0.1	glucose, phosphate limitation	[391]
Escherichia	*coli*	JW135	0.46			37	6.4	0.1	glucose, nitrogen limitation	[391]
Escherichia	*coli*	JW135	0.12			37	6.4	0.3	glucose, glucose limitation	[391]
Escherichia	*coli*	JW135	0.137			37	6.4	0.3	glucose, phosphate limitation	[391]
Escherichia	*coli*	JW135	0.139			37	6.4	0.3	glucose, nitrogen limitation	[391]
Escherichia	*coli*	JW135	0.16			37	6.4	0.3	glucose, sulphur limitation	[391]
Escherichia	*coli*	SR14		105		37	6.0	0.5	formate	[353]
Escherichia	*coli*	SR14		125		37	6.0	1	formate	[353]
Escherichia	*coli*	SR14		144.2		37	6.0	2	formate	[353]
Ethanoligenens	*harbinense*	YUAN-3	1.92		32.2	35	5.0	0.4	glucose	[279]
Klebsiella	*oxytoca*	HP1	3.6	15.2	15.62	38	6.5		sucrose	[286]
Klebsiella	*pneumoniae*	DSM 2026		12.2		37	6.8	0.16	glycerol	[392]
Klebsiella	*pneumoniae*	DSM 2026		16		37	7.0	0.2	glycerol	[392]
Klebsiella	*pneumoniae*	DSM 2026		15		37	7.0	0.4	glycerol	[392]
Klebsiella	*pneumoniae*	DSM 2026		29.6					glycerol	[118]
Pyrococcus	*furiosus*	DSM 3638	3.8		6.24	90		0.45	cellobiose	[393]
Pyrococcus	*furiosus*	DSM 3638			3.66	90		0.45	cellobiose, sodium sulphide	[393]
Pyrococcus	*furiosus*	DSM 3638	2.6		2.35	90		0.45	maltose	[393]
Pyrococcus	*furiosus*	DSM 3638		69			6.5		maltose, no sulphur source used	[394]
Pyrococcus	*furiosus*	DSM 3638		102			6.5		maltose, sulphur source used	[394]
Ruminococcus	*albus*	DSM 20455	1			37		0.024	sorghum extract	[291]
Ruminococcus	*albus*	DSM 20455	1.07			37	6.4-6.5	0.024	glucose	[292]
Ruminococcus	*albus*	DSM 20455	0.47			37	6.4-6.5	0.0278	glucose	[292]
Ruminococcus	*albus*	DSM 20455	0.54			37	6.4-6.5	0.042	glucose	[292]

Ruminococcus	*albus*	strain 7	2.11-2.49				6.7-6.8	0.17	glucose	[395]
Ruminococcus	*albus*	strain 7	1.76-2.75				6.7-6.8	0.25	glucose	[395]
Ruminococcus	*albus*	strain 7	2.78-3.1				6.7-6.8	0.33	glucose	[395]
Ruminococcus	*albus*	strain 7	1.70				6.7-6.8	0.49	glucose	[395]
Ruminococcus	*albus*	strain 7	1.72-2.64				6.7-6.8	0.59	glucose	[395]
Ruminococcus	*flavefaciens*	FD-1	0.376-0.590				6.56		cellulose	[396]
Thermococcus	*kodakaraensis*	JCM 12380	1.09	24.9	3.88	85	6.8	0.2	pyruvate	[397]
Thermococcus	*kodakaraensis*	JCM 12380	3.33	14.0	3.16	85	6.8	0.2	starch	[397]
Thermococcus	*kodakaraensis*	JCM 12380		59.6		85	6.8	0.8	pyruvate	[397]
Thermotoga	*elfii*		4			65			biomass	[398]
Enterobacter<br>*Enterobacter*<br>*Enterobacter*	*aerogenes*<br>*cloacae*<br>*sakazakii*	IAM 12348<br>IAM 12349<br>IAM 12660	0.62		1.56	35	5.5	0.167	3000 ppm bean curd waste	[399]
Enterobacter<br>*Enterobacter*<br>*Enterobacter*	*aerogenes*<br>*cloacae*<br>*sakazakii*	IAM 12348<br>IAM 12349<br>IAM 12660	0.16		0.54	35	5.5	0.167	4000 ppm bean curd waste	[399]
Enterobacter<br>*Enterobacter*<br>*Enterobacter*	*aerogenes*<br>*cloacae*<br>*sakazakii*	IAM 12348<br>IAM 12349<br>IAM 12660	0.52		2.23	35	5.5	0.167	5000 ppm bean curd waste	[399]
Clostridium<br>*Enterobacter*	*butyricum*<br>*aerogenes*	IFO 13949<br>HO-39	2.1		22.3	36	5.2	0.2	starch	[122]
Clostridium<br>*Enterobacter*	*butyricum*<br>*aerogenes*	IFO 13949<br>HO-39	2.5		35.7	36	5.2	0.5	starch	[122]
Clostridium<br>*Enterobacter*	*butyricum*<br>*aerogenes*	IFO 13949<br>HO-39	2.6		58	36	5.2	1	starch	[122]
Citrobacter<br>*Enterobacter*	*freundii*<br>*cloacae*	Cf1<br>Ecl	2.33		35	37		0.11	sucrose	[400]
Citrobacter<br>*Enterobacter*	*freundii*<br>*cloacae*	Cf1<br>Ecl	2.33		180	37		0.22	sucrose	[400]
Ruminococcus<br>*Vibrio*	*albus*<br>*succinogenes*	strain 7	3.76				6.7-6.8	0.11	glucose	[395]
Ruminococcus<br>*Vibrio*	*albus*<br>*succinogenes*	strain 7	3.59				6.7-6.8	0.12	glucose	[395]
Ruminococcus<br>*Vibrio*	*albus*<br>*succinogenes*	strain 7	3.91-3.98				6.7-6.8	0.19	glucose	[395]

Ruminococcus<br>*Vibrio*	*albus*<br>*succinogenes*	strain 7	3.83				6.7-6.8	0.33	glucose	[395]
Ruminococcus<br>*Vibrio*	*albus*<br>*succinogenes*	strain 7	4.59				6.7-6.8	0.35	glucose	[395]
Ruminococcus<br>*Vibrio*	*albus*<br>*succinogenes*	strain 7	3.13-3.89				6.7-6.8	0.65	glucose	[395]
Caldicellulosiruptor<br>*Caldicellulosiruptor*	*saccharolyticus*<br>*kristjanssonii*	DSM 8903<br>DSM 12137	3.5	12.7	3.8	70	6.7	0.04	5 g/L glucose, 5 g/L xylose	[34]
Caldicellulosiruptor<br>*Caldicellulosiruptor*	*saccharolyticus*<br>*kristjanssonii*	DSM 8903<br>DSM 12137	3.6	14.2	4.3	70	6.7	0.06	5 g/L glucose, 5 g/L xylose	[34]
Caldicellulosiruptor<br>*Caldicellulosiruptor*	*saccharolyticus*<br>*kristjanssonii*	DSM 8903<br>DSM 12137	3.5	15.5	6.2	70	6.7	0.08	5 g/L glucose, 5 g/L xylose	[34]
Caldicellulosiruptor<br>*Caldicellulosiruptor*	*saccharolyticus*<br>*kristjanssonii*	DSM 8903<br>DSM 12137	3.1	15	8.3	70	6.7	0.12	5 g/L glucose, 5 g/L xylose	[34]
Caldicellulosiruptor<br>*Caldicellulosiruptor*	*saccharolyticus*<br>*kristjanssonii*	DSM 8903<br>DSM 12137	2.9	18.4	10.3	70	6.7	0.15	5 g/L glucose, 5 g/L xylose	[34]
Caldicellulosiruptor<br>*Caldicellulosiruptor*	*saccharolyticus*<br>*kristjanssonii*	DSM 8903<br>DSM 12137	2.8	18.3	11	70	6.7	0.2	5 g/L glucose, 5 g/L xylose	[34]
Caldicellulosiruptor<br>*Caldicellulosiruptor*	*saccharolyticus*<br>*kristjanssonii*	DSM 8903<br>DSM 12137	2.9	21	11.6	70	6.7	0.25	5 g/L glucose, 5 g/L xylose	[34]
Caldicellulosiruptor<br>*Caldicellulosiruptor*	*saccharolyticus*<br>*kristjanssonii*	DSM 8903<br>DSM 12137	2.5	21	11.6	70	6.7	0.3	5 g/L glucose, 5 g/L xylose	[34]
Caldicellulosiruptor<br>*Caldicellulosiruptor*	*saccharolyticus*<br>*kristjanssonii*	DSM 8903<br>DSM 12137	3.6	10.2	4.5	70	6.7	0.06	4 g/L glucose, 4 g/L xylose	[34]
Caldicellulosiruptor<br>*Caldicellulosiruptor*	*saccharolyticus*<br>*kristjanssonii*	DSM 8903<br>DSM 12137	3.2	24.7	8.6	70	6.7	0.15	4 g/L glucose, 4 g/L xylose	[34]
Caldicellulosiruptor<br>*Caldicellulosiruptor*	*saccharolyticus*<br>*kristjanssonii*	DSM 8903<br>DSM 12137	3.7	14.8	4.8	70	6.7	0.06	glucose	[34]
Caldicellulosiruptor<br>*Caldicellulosiruptor*	*saccharolyticus*<br>*kristjanssonii*	DSM 8903<br>DSM 12137	3.5	21.4	10.4	70	6.7	0.15	glucose	[34]
Caldicellulosiruptor<br>*Caldicellulosiruptor*	*saccharolyticus*<br>*kristjanssonii*	DSM 8903<br>DSM 12137	2.7	22.6	3	70	6.7	0.06	xylose	[34]
Caldicellulosiruptor<br>*Caldicellulosiruptor*	*saccharolyticus*<br>*kristjanssonii*	DSM 8903<br>DSM 12137	2.7	33	8.5	70	6.7	0.06	xylose	[34]
Clostridium<br>*Thermoanaerobacterium*	sp.<br>sp.	AK 15<br>AK 17	0.39		0.78	60	6.0	0.16	2.0 mmol glucose L^{-1} h^{-1}	[331]
Clostridium<br>*Thermoanaerobacterium*	sp.<br>sp.	AK 15<br>AK 17	0.53		1.5	60	6.0	0.16	2.8 mmol glucose L^{-1} h^{-1}	[331]
Clostridium<br>*Thermoanaerobacterium*	sp.<br>sp.	AK 15<br>AK 17	0.55		2.18	60	6.0	0.16	4.0 mmol glucose L^{-1} h^{-1}	[331]
Clostridium<br>*Thermoanaerobacterium*	sp.<br>sp.	AK 15<br>AK 17	0.8		4.43	60	6.0	0.24	glucose	[331]

Clostridium<br>*Thermoanaerobacterium*	sp.<br>sp.	AK 15<br>AK 17	0.8		6.1	60	6.0	0.32	glucose	[331]
Clostridium<br>*Thermoanaerobacterium*	sp.<br>sp.	AK 15<br>AK 17	0.63		5.31	60	6.0	0.36	glucose	[331]

+ for reference please refer to manuscript
