# Supplementary material for: A comprehensive and quantitative review of dark fermentative biohydrogen production
Source: Microb Cell Fact. 2012 Aug 27;11:115. doi: 10.1186/1475-2859-11-115 (PMC3443015; doi:10.1186/1475-2859-11-115)
Supplement: Additional file 5 — Fed-batch dark fermentative biohydrogen production[38]. [file 1475-2859-11-115-S5.pdf]

Additional file 5: Fed-batch dark fermentative biohydrogen production

Genus	Species	Strain	$Y_{(H2/S)}$ [mol mol^{-1}]	Temperature [°C]	pH	Substrate and discriminative condition	Reference+
Clostridium	*tyrobutyricum*	ATCC 25755	1.43	37	6.0	glucose, free cells	[38]
Clostridium	*tyrobutyricum*	ATCC 25755 recombinant	1.56	37	5.0	xylose, cells immobilized	[38]
Clostridium	*tyrobutyricum*	ATCC 25755 recombinant	2.06	37	6.0	glucose, cells immobilized	[38]
Clostridium	*tyrobutyricum*	ATCC 25755 recombinant	2.06	37	5.0	glucose, cells immobilized	[38]
Clostridium	*tyrobutyricum*	ATCC 25755 recombinant	2.15	37	6.0	glucose, free cells	[38]

+ for reference please refer to manuscript
